# Endothelial ERα promotes glucose tolerance by enhancing endothelial insulin transport to skeletal muscle

**DOI:** 10.1038/s41467-023-40562-w

**Published:** 2023-08-17

**Authors:** Anastasia Sacharidou, Ken Chambliss, Jun Peng, Jose Barrera, Keiji Tanigaki, Katherine Luby-Phelps, İpek Özdemir, Sohaib Khan, Shashank R. Sirsi, Sung Hoon Kim, Benita S. Katzenellenbogen, John A. Katzenellenbogen, Mohammed Kanchwala, Adwait A. Sathe, Andrew Lemoff, Chao Xing, Kenneth Hoyt, Chieko Mineo, Philip W. Shaul

**Affiliations:** 1 Center for Pulmonary and Vascular Biology, Department of Pediatrics, University of Texas Southwestern Medical Center, Dallas, TX 75390 USA; 2 Department of Cell Biology, University of Texas Southwestern Medical Center, Dallas, TX 75390 USA; 3 Department of Bioengineering, University of Texas at Dallas, Richardson, TX 75080 USA; 4 University of Cincinnati Cancer Institute, Department of Cancer and Cell Biology, University of Cincinnati College of Medicine, Cincinnati, OH 45219 USA; 5 Department of Chemistry, University of Illinois at Urbana-Champaign, Urbana, IL 61801 USA; 6 Departments of Physiology and Cell Biology, University of Illinois at Urbana-Champaign, Urbana, IL 61801 USA; 7 Eugene McDermott Center for Human Growth and Development, University of Texas Southwestern Medical Center, Dallas, TX 75390 USA; 8 Department of Biochemistry, University of Texas Southwestern Medical Center, Dallas, TX 75390 USA; 9 Lyda Hill Department of Bioinformatics, University of Texas Southwestern Medical Center, Dallas, TX 75390 USA

**Keywords:** Nuclear receptors, Type 2 diabetes, Type 2 diabetes

## Abstract

The estrogen receptor (ER) designated ERα has actions in many cell and tissue types that impact glucose homeostasis. It is unknown if these include mechanisms in endothelial cells, which have the potential to influence relative obesity, and processes in adipose tissue and skeletal muscle that impact glucose control. Here we show that independent of impact on events in adipose tissue, endothelial ERα promotes glucose tolerance by enhancing endothelial insulin transport to skeletal muscle. Endothelial ERα-deficient male mice are glucose intolerant and insulin resistant, and in females the antidiabetogenic actions of estradiol (E2) are absent. The glucose dysregulation is due to impaired skeletal muscle glucose disposal that results from attenuated muscle insulin delivery. Endothelial ERα activation stimulates insulin transcytosis by skeletal muscle microvascular endothelial cells. Mechanistically this involves nuclear ERα-dependent upregulation of vesicular trafficking regulator sorting nexin 5 (SNX5) expression, and PI3 kinase activation that drives plasma membrane recruitment of SNX5. Thus, coupled nuclear and non-nuclear actions of ERα promote endothelial insulin transport to skeletal muscle to foster normal glucose homeostasis.

## Abstract (web summary)

Estrogen has anti-diabetic effects via estrogen receptor alpha (ERα). Here, authors show that via coupled nuclear and non-nuclear actions, ERα in endothelial cells promotes insulin transport to skeletal muscle to foster normal glucose homeostasis.

## Introduction

In addition to their classic roles in reproduction, estrogens have a potent impact on glucose homeostasis^1,2^. In female rodents and primates, ovariectomy leads to glucose intolerance and insulin resistance particularly in the setting of diet-induced obesity, and these effects are reversed by estrogens^2–5^. Estrogen receptors (ER) are members of the steroid hormone receptor superfamily that traditionally serve as transcription factors^6^, and the ER designated ERα modulates multiple aspects of glucose regulation^2–5^. The known relevant sites of action include the CNS, liver, skeletal muscle myocytes, pancreas, macrophages and adipose tissue^7–12^. In women, surgical or natural menopause increases the age-adjusted odds ratio of developing diabetes to 1.59 (CI 1.07-2.37) compared to the rate in premenopausal women^13^. In postmenopausal women, changes in glucose homeostasis also occur^14^, and hormone replacement therapy (HRT) with estrogens lowers the risk of developing diabetes^15^. In men, individuals with disruptive mutations in the ERα gene or the gene encoding aromatase, which produces estrogens by aromatizing androgens, have decreased glucose tolerance, and in the latter category 17β-estradiol (E2) administration improves glucose tolerance^16–18^. In parallel, male aromatase^-/-^ mice have insulin resistance that is reversed by E2^19^. Thus, estrogens and ERα have important impact on glucose homeostasis in both males and females.

Whereas the metabolic effects of estrogens partnering with ERα are well known in many cell types and tissues including adipocytes and skeletal muscle myocytes, the participation of ERα in endothelium in glucose control is unknown. Interestingly, in addition to predictably regulating endothelial gene expression, in endothelial cells there is a plasma membrane-associated subpopulation of ERα which uniquely mediates numerous non-nuclear processes, including E2 activation of endothelial NO synthase (eNOS)^20–23^. Mechanisms in endothelial cells influence both relative obesity and other processes in adipose that impact glucose homeostasis. Effective vascularization is essential to the healthy expansion of adipose tissue^24^, the endothelium governs adipose tissue inflammation and thereby influences adipokine and cytokine production^25^, and endothelial ERα actions are both proangiogenic and anti-inflammatory^26–28^. Based on these rationales, in the current work, experiments were designed in mice to determine if and how ERα in endothelium influences glucose homeostasis. They revealed that endothelial ERα excision in both male and female mice leads to glucose intolerance and insulin resistance, and surprisingly this was not due to changes in adiposity or processes in adipose that influence glucose control. Efforts then turned to the interrogation of endothelial mechanisms in other tissues involved in glucose control, and they uncovered a defect in skeletal muscle glucose disposal related to an impairment in muscle insulin delivery. Further work ultimately identified coupled nuclear and non-nuclear actions of ERα which promote endothelial insulin transport by upregulating expression of the vesicular trafficking regulator sorting nexin 5 (SNX5) and by activating PI3 kinase to drive plasma membrane recruitment of SNX5. These findings reveal a mode of action of estrogens and ERα that may represent a set of targets to leverage to improve glucose tolerance and insulin sensitivity.

## Results

### Endothelial ERα promotes glucose control by enhancing muscle insulin delivery

**Fig. 1:**
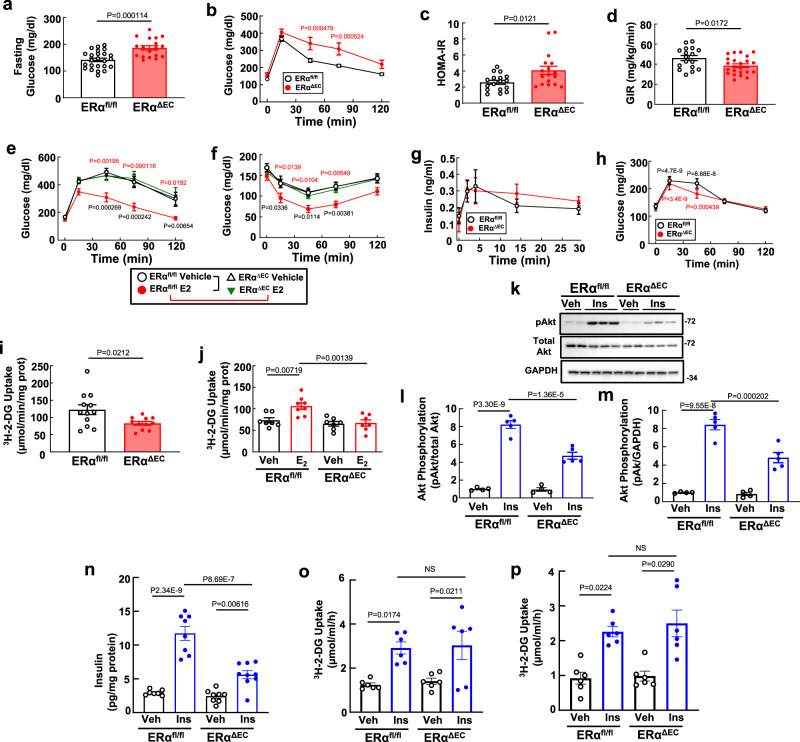
Endothelial ERα promotes glucose tolerance and insulin sensitivity by enhancing insulin delivery to skeletal muscle. **a**–**c** Fasting glucose (**a**, *n* = 24 and 18 mice) and glucose tolerance tests (**b**, GTT, *n* = 14 and 9 mice) were evaluated in male ERα^fl/fl^ and ERα^ΔEC^ mice, and HOMA-IR was determined (**c**, *n* = 17 and 17). **d** Additional males underwent hyperinsulinemic, euglycemic clamps and glucose infusion rate (GIR) was calculated (*n* = 17 and 23). **e**, **f** Ovariectomized high fat diet-fed female mice were administered vehicle or E2 for 12 weeks, and GTT (**e**) and insulin tolerance tests (**f**) were performed. In ERα^fl/fl^ given vehicle versus E2, and ERα^ΔEC^ given vehicle versus E2, *n* = 9, 10, 8 and 9 mice (**e**), and 11, 11, 10, and 10 mice (**f**). **g** In standard chow-fed males, plasma insulin levels were measured before and after IP D-glucose (*n* = 9 and 6 in ERα^fl/fl^ and ERα^ΔEC^). **h** Males also underwent pyruvate tolerance tests (*n* = 7 and 8 mice in ERα^fl/fl^ and ERα^ΔEC^). **i**, **j** Skeletal muscle glucose uptake was determined in males (**i**, *n* = 12 and 11 mice), and in ovariectomized, high fat diet-fed females following receipt of vehicle or E2 for 12 weeks (**j**, *n* = 7,8,7 and 7 mice). **k**–**n** Standard chow-fed males were injected with vehicle (Veh, saline) or insulin (ins, 1 unit/kg), 5 min later skeletal muscle was isolated, and lysates prepared for immunoblotting for phosphorylated Akt (Ser 473, pAkt), total Akt and GAPDH. **k** Example findings for 2 to 3 samples/group, and quantification for *n* = 4,5, 4 and 5 mice using total Akt or GAPDH as denominator (**l**, **m**). In muscle samples from mice studied as in (**k**), insulin content was quantified (**n**, *n* = 7,8,8 and 9 mice). **o**, **p** Insulin-stimulated glucose uptake was assessed ex vivo in soleus (**o**) and EDL (**p**) from male ERα^fl/fl^ and ERα^ΔEC^ mice (*n* = 6 mice/group). Data are mean ± SEM. *P* values by two-sided Student’s *t* test (**a**, **d**, **i**), Mann–Whitney testing (**c**), two-way ANOVA with Sidak’s post-hoc testing (**b**, **e**, **f**, **h**), one-way ANOVA with Tukey’s post-hoc testing (**j**,**l**–**o**), or Krusakal–Wallis with Dunn’s post-hoc testing (**p**) are shown. Source data are provided as a Source Data file.

To begin determining how ERα in endothelium impact glucose homeostasis, fasting glucose and glucose tolerance tests (GTT) were performed in control floxed ERα mice (ERα^fl/fl^) and mice selectively deficient in the receptor in endothelial cells (ERα^ΔEC^, Supplementary Fig. 1a, b)^29^. Male ERα^ΔEC^ mice displayed fasting hyperglycemia and abnormal glucose tolerance tests (GTT), and they also had elevated HOMA-IR, and decreased glucose infusion rates (GIR) during a hyperinsulinemic, euglycemic clamp (Fig. 1a–d). In HFD-fed, ovariectomized females whereas E2 caused a dramatic normalization of GTT in ERα^fl/fl^ controls, the capacity of E2 to prevent HFD-induced glucose intolerance was completely absent in ERα^ΔEC^ (Fig. 1e). This finding is consistent with a prior study limited to GTTs that demonstrated glucose intolerance in HFD-fed intact female mice deficient in endothelial ERα^30^. Insulin tolerance tests (ITT) further revealed that the previously recognized capacity for E2 to improve insulin sensitivity is entirely absent in ERα^ΔEC^ females (Fig. 1f).

In light of numerous mechanisms by which endothelial cells in adipose tissue influence relative adiposity and glucose control, body composition was next evaluated. Standard chow-fed male ERα^fl/fl^ and ERα^ΔEC^ mice had similar body weights, percent body fat and lean body mass (Supplementary Fig. 1c–e), and subcutaneous (inguinal) and visceral (gonadal) white adipose tissue (WAT) depots were similar in size (Supplementary Fig. 1f, g). In ovariectomized high-fat diet (HFD)-fed females, there was a partial blunting of E2-related body weight loss in ERα^ΔEC^, but E2-induced decreases in fat mass and increases in lean body mass were unaffected by endothelial ERα deficiency (Supplementary Fig. 1h–j). Thus, endothelial ERα does not influence adiposity in male mice or mediate the well-recognized protection from adiposity by E2 in female mice^5^.

The basis by which endothelial cell ERα influences glucose homeostasis was then further pursued in male mice. Recognizing that adipose tissue vascularity impacts insulin sensitivity^24^, transcript abundance for the endothelial markers Flk1 and PECAM1 was evaluated in subcutaneous and visceral WAT, and it was similar in ERα^fl/fl^ and ERα^ΔEC^ mice (Supplementary Fig. 2a–d). Since the endothelium within fat depots governs the degree of inflammation^25^, inflammatory cell abundance and cytokine expression were evaluated (Supplementary Fig. 2e–l), and levels of the macrophage marker F4/80, the leukocyte marker CD11, and IL-6 and TNFα transcripts were similar in the WAT depots of the two genotype groups. As such, endothelial ERα does not influence adipose tissue vascularity or inflammation, and the impact of endothelial ERα on glucose homeostasis entails processes other than those in adipose microvasculature that impact insulin sensitivity^24,25^.

Since the endothelium in the pancreas influences insulin secretion from beta cells^31^, pancreatic insulin production was evaluated, and increases in plasma insulin levels in response to an acute glucose load were similar in ERα^fl/fl^ versus ERα^ΔEC^ mice (Fig. 1g). Despite this finding, the insulin response of the pancreas may not be fully preserved in the ERα^ΔEC^ mice because they display relative fasting hyperglycemia that is not compensated by an increase in insulin secretion. Noting that processes in hepatic endothelium influence insulin action in the liver^32^, possible changes in hepatic insulin sensitivity were studied using pyruvate tolerance tests, and they were similar in ERα^ΔEC^ and ERα^fl/fl^ mice (Fig. 1h). Thus, ERα in endothelium does not impact hepatic insulin sensitivity, and if there is an impact on pancreatic insulin secretion, it is quite modest.

Next, we interrogated processes in the skeletal muscle, where up to 80% of whole-body glucose disposal occurs in both humans and rodents^33,34^. Glucose uptake in soleus and gastrocnemius was decreased by 33% in ERα^ΔEC^ males (Fig. 1i), and in HFD-fed ovariectomized females, whereas E2 caused a 47% increase in glucose uptake in ERα^fl/fl^ controls, the increase in uptake with E2 was completely absent in ERα^ΔEC^ (Fig. 1j). Insulin action in skeletal muscle was then queried in males by quantifying the activation of Akt kinase^35^, and compared to ERα^fl/fl^ controls, Akt Ser473 phosphorylation in response to insulin was attenuated in ERα^ΔEC^ employing either total Akt or GAPDH as the denominator (Fig. 1k–m, Supplementary Fig. 3a, b). Recognizing that mechanisms in the endothelium are critically involved in insulin delivery to the skeletal muscle myocytes^36–39^, muscle insulin delivery was tested. Whereas there was a 2.7- to 3-fold increase in skeletal muscle insulin content 5 min after bovine insulin injection in ERα^fl/fl^ controls, the increase was blunted by 60 to 64% in ERα^ΔEC^ mice (Fig. 1n, Supplementary Fig. 3c). To determine if endothelial ERα impacts a process specifically in endothelial cells and not in myocytes to alter muscle glucose uptake, we evaluated insulin-induced glucose uptake in isolated skeletal muscles ex vivo (Fig. 1o, p). In both soleus and EDL insulin-induced glucose uptake ex vivo was identical in muscle from ERα^fl/fl^ and ERα^ΔEC^ mice. This indicates that there are no changes in skeletal muscle myocyte processes regulating glucose homeostasis with endothelial ERα silencing independent of the effects on endothelial insulin transport. Thus, through endothelium-based mechanisms, ERα in endothelium supports skeletal muscle glucose disposal by promoting insulin delivery to muscle.

### Endothelial ERα promotes endothelial insulin transport via both nuclear and non-nuclear mechanisms

**Fig. 2:**
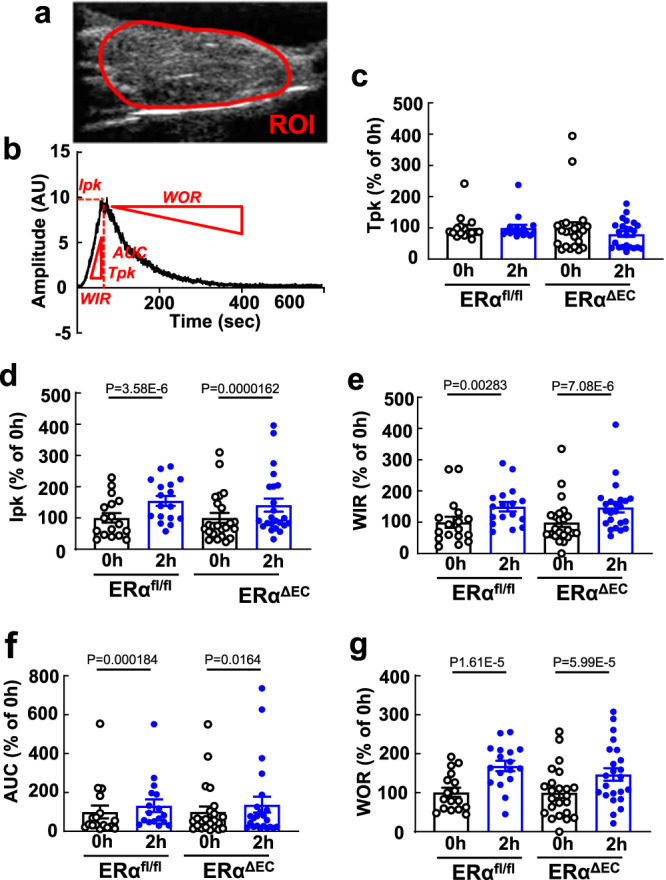
Endothelial ERα does not influence insulin-induced capillary recruitment in skeletal muscle. To evaluate insulin-stimulated increases in skeletal muscle capillary recruitment, contrast-enhanced ultrasound (CEUS) was performed in male ERα^fl/fl^ and ERα^ΔEC^ mice at baseline (0 h) and at 2 h of hyperinsulinemic-euglycemic clamp. *N* = 17 for ERα^fl/fl^ mice and *n* = 23 for ERα^ΔEC^ mice. **a** Representative CEUS still image of the hindlimb adductor muscle group. ROI= region of interest. **b** Representative CEUS time-intensity curve. Ipk=peak intensity, Tpk=time to peak intensity, WIR wash-in rate, AUC area under the curve, and WOR wash-out rate. **c**–**g** Findings for Tpk (**c**), Ipk (**d**), WIR (**e**), AUC (**f**), and WOR (**g**). Data are mean ± SEM. P values by Wilcoxon matched-pairs signed rank test (**c**–**f**), two-sided paired Student’s *t* test (**g**) are shown. Source data are provided as a Source Data file.

How endothelial ERα positively influences skeletal muscle insulin delivery was then determined. Since muscle insulin delivery and resulting glucose disposal are enhanced by insulin stimulation of skeletal muscle microvascular recruitment and blood flow^40–42^, the impact of endothelial ERα on muscle microvascular functional responses to insulin was evaluated. This was accomplished using contrast-enhanced ultrasound (CEUS) with a lipid-shelled perfluorocarbon-based microbubble (MB) contrast agent to image the muscle microvasculature before and during a hyperinsulinemic-euglycemic clamp. A representative still image of a CEUS region-of-interest (ROI) is shown in Fig. 2a, and a sample time-intensity curve is in Fig. 2b. Analysis of the curve yields parametric measures of capillary blood volume (CBV: peak intensity, *Ipk*), microvascular blood flow (MBF: time to peak intensity, *Tpk*; and wash-in rate, *WIR*), and both CBV and MBF (area under the curve, *AUC*; and wash-out rate, *WOR*)^43–48^. Supplementary Movies 1 and 2 show real-time CEUS imaging of MB in the skeletal muscle microvasculature of an ERα^fl/fl^ control mouse at baseline and during a clamp, respectively. The greater MB signal seen in the clamp movie, particularly early in the movie, reveals how insulin increases capillary recruitment and blood flow in the skeletal muscle. In the ERα^fl/fl^ control mice whereas there was no change in Tpk in response to insulin (Fig. 2c), Ipk, WIR, AUC and WOR increased (Fig. 2d–g), indicative of insulin-induced increases in CBV and MBF. Equal increases in Ipk, WIR, AUC and WOR occurred in ERα^ΔEC^, revealing that the promotion of muscle insulin delivery by endothelial ERα is not related to effects on insulin-induced microvascular recruitment and blood flow.

Since changes in intracellular vesicles have been previously observed in skeletal muscle capillary endothelium in mice with obesity-related insulin resistance^49^, such endothelial vesicles were then compared in ERα^fl/fl^ and ERα^ΔEC^ mice (Supplementary Fig. 4a). Between genotype groups, the total number of vesicles per unit area of endothelium was similar, as was the number of vesicles associated with the luminal plasma membrane or the abluminal plasma membrane, or localized intracellularly (Supplementary Fig. 4b, c). Interestingly, independent of genotype group, the number of vesicles associated with the abluminal plasma membrane was greater than those on the luminal plasma membrane or those localized intracellularly. Parallel findings were obtained regarding vesicle area relative to the endothelial area (Supplementary Fig. 4d, e). In addition, vesicle size was similar in ERα^fl/fl^ and ERα^ΔEC^ mice, and it was comparable in the three categories of vesicles (Supplementary Fig. 4f, g). Thus, changes in the frequency or size of capillary endothelial vesicles do not explain the observed differences in skeletal muscle insulin delivery in ERα^fl/fl^ and ERα^ΔEC^ mice.

**Fig. 3:**
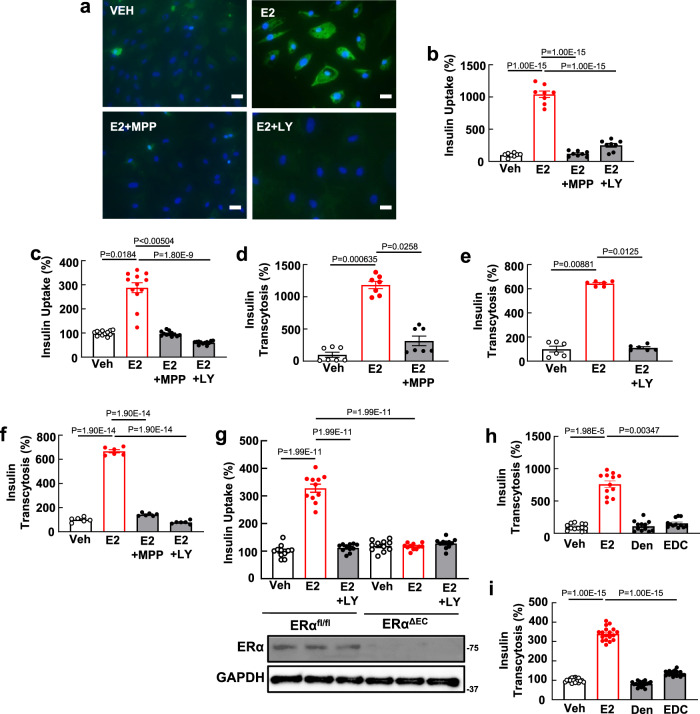
Endothelial ERα promotes insulin transport by skeletal muscle microvascular endothelial cells via nuclear and non-nuclear actions of the receptor, with the latter entailing activation of PI3 kinase. **a**–**c**. HAEC (**a**, **b**) or HSMEC (**c**) were incubated with FITC-insulin (5 × 10^−5^ M) and vehicle or E2 (10^−8^ M), in the absence or presence of the selective ERα antagonist methyl-piperidino-pyrazone (MPP, 1 × 10^−5^M) or the PI3 kinase inhibitor LY294002 (5 × 10^−5^M) for 30 min, and insulin uptake was evaluated by fluorescence microscopy (**a**; HAEC, bar=50um) or by FITC-insulin uptake assay (**b**, **c**; HAEC and HSMEC, *n* = 8 and 12 wells of cells per treatment, respectively). **d** HAEC were grown in transwells to confluency, FITC-insulin was added to the upper chamber along with vehicle, E2, or E2 plus MPP, and the amount of insulin transcytosed to the lower chamber was evaluated after 30 min, *n* = 7 transwells per treatment. **e** The effect of LY294002 on E2-induced insulin transcytosis was also determined in HAEC, *n* = 6. **f** Parallel experiments were performed evaluating insulin transcytosis in HSMEC, evaluating the effect of E2 in the absence versus presence of MPP or LY294002, *n* = 6 transwells per treatment. **g** Skeletal muscle microvascular endothelial cells from male ERα^fl/fl^ and ERα^ΔEC^ mice were incubated with FITC-insulin and vehicle or E2 in the absence or presence of LY294002, and insulin uptake was evaluated by FITC-insulin uptake assay (upper panel, *n* = 11 wells of cells per treatment). ERα abundance was evaluated by immunoblotting (lower panel). **h**, **i** To determine whether non-nuclear ER activation is sufficient to activate insulin transcytosis in HAEC (**h**, *n* = 12 transwells per treatment) and HSMEC (**i**, n = 18 transwells per treatment), the effect of E2 was compared to that of the estrogen dendrimer conjugate (EDC) at equimolar concentrations of E2, and empty dendrimer (Den) served as control. Data are mean ± SEM. *P* values by one-way ANOVA with Tukey’s post-hoc testing (**b**, **f**, **g**, **i**), and Kruskal–Wallis with Dunn’s posthoc testing (**c**–**e**, **h**) are shown. Source data are provided as a Source Data file.

Along with insulin-related changes in CBV and MBF, insulin delivery to skeletal muscle is controlled by processes governing its transcytosis across the endothelial monolayer, and there is evidence that the latter is rate-limiting for peripheral insulin action^36–39^. We therefore determined if ERα influences insulin transport by endothelial cells, first interrogating insulin uptake, the initial step in transcytosis, in human aortic endothelial cells (HAEC). As visualized in Fig. 3a and quantified in Fig. 3b, E2 caused a dramatic increase in the uptake of FITC-conjugated insulin, and the response was negated by the selective ERα antagonist methyl-piperidino-pyrazone (MPP). Knowing that PI3 kinase mediates many of the processes governed by the subpopulation of non-nuclear ERα in endothelial cells^50^, the effect of the PI3 kinase inhibitor LY294002 was tested, and it too fully prevented the stimulation of insulin uptake by E2 (Fig. 3a, b). Since the in vivo observations pertain to skeletal muscle microvascular endothelium, we then studied human skeletal muscle microvascular endothelial cells (HSMEC), and found that E2 causes an ERα- and PI3 kinase-dependent increase in insulin uptake in HSMEC (Fig. 3c). Endothelial insulin transcytosis was then evaluated, and E2 caused a more than 10-fold ERα- and PI3 kinase-dependent increase in insulin transcytosis in HAEC (Fig. 3d, e). Importantly, parallel findings for insulin transcytosis were made in HSMEC (Fig. 3f). In the transwell assays transendothelial electric resistance (TEER) was in the range of 350-450 Ωcm^2^ and similar in the various study groups within each experiment (Supplemental Fig. 5a–c), indicating that comparable monolayer barriers were present, and strengthening the evidence that the insulin transport being interrogated is transcellular^51^. In primary mouse skeletal muscle endothelial cells, in which studies of insulin uptake are feasible, E2 caused a 3.2-fold increase in uptake in endothelium from ERα^fl/fl^ mice which was prevented by PI3 kinase inhibition, and uptake was not stimulated by E2 in endothelium from ERα^ΔEC^ mice (Fig. 3g). These findings strengthen the evidence that ERα and PI3 kinase are required, and they help link the in vivo and cell culture observations.

Demonstrating a requirement for PI3 kinase activation, we then determined if non-nuclear actions of endothelial ERα are sufficient to promote endothelial insulin transcytosis employing the estrogen dendrimer conjugate (EDC), which selectively activates non-nuclear ER^26^. In contrast to E2, EDC did not stimulate insulin transcytosis in either HAEC or HSMEC (Fig. 3h, i, Supplementary Fig. 5d, e). This observation is consistent with our prior demonstration in ovariectomized, HFD-fed female mice that EDC does not afford the protection from insulin resistance observed with E2 treatment^52^. Thus, both the initial step of insulin uptake and the capacity of endothelial cells to transcytose insulin are potently stimulated by endothelial ERα activation, and these processes require both nuclear and non-nuclear actions of the receptor, with the latter entailing PI3 kinase activation.

### Interrogating the endothelial ERα interactome and transcriptome

**Fig. 4:**
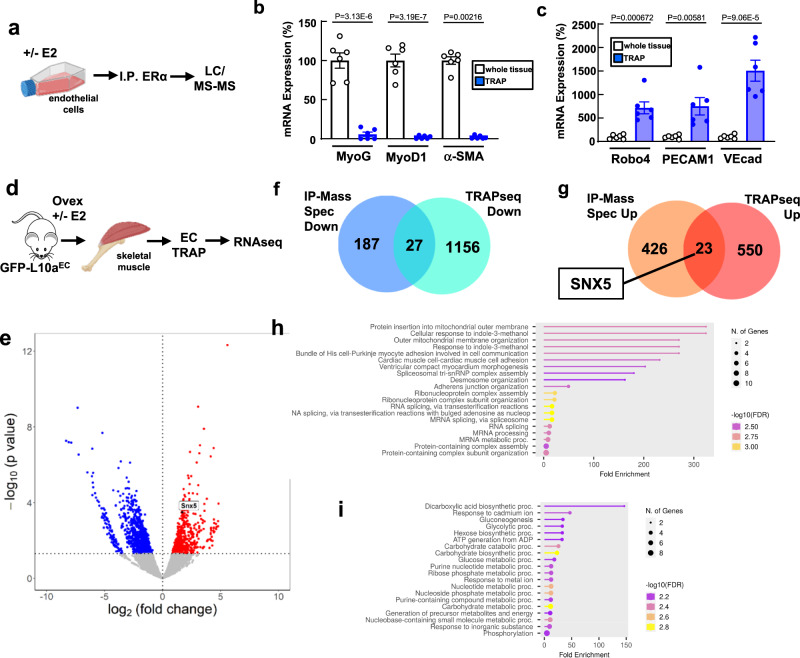
Interrogating non-nuclear and nuclear ERα functions in endothelial cells. **a** Schematic for IP-mass spec experiment in cultured endothelial cells. Created by BioRender.com. **b**, **c** Establishment of TRAPseq strategy in skeletal muscle microvascular endothelium. Skeletal muscle was harvested from GFP-L10a^EC^ mice, total RNA was isolated (whole tissue), ribosome-bound RNA was isolated from endothelium (TRAP), and Q-RT-PCR was done to detect (**b**) the skeletal muscle-specific genes myogenin (MyoG), myosin D1 (MyoD1) and α-smooth muscle myosin (α-SMA), and (**c**) the endothelium-specific genes roundabout (Robo), PECAM-1 and VECad. In (**b**, **c**) values are mean ± SEM, *n* = 6 mice, and *p* values by two-sided Student’s *t* test are shown. **d** TRAPseq was performed on skeletal muscle endothelial cells from ovariectomized female mice treated with vehicle versus E2 for 4 weeks. Created by BioRender.com. **e** Volcano plot for TRAPseq findings for E2 downregulation and upregulation of gene translation in skeletal muscle microvascular endothelial cells in vivo in mice. **f** Venn diagram of proteins which disassociate from ERα (IP-mass spec) in cultured endothelial cells in response to E2 and genes whose translation is downregulated by E2 in skeletal muscle endothelial cells in vivo (TRAPseq, *p* ≤ 0.05). **g** Venn diagram of proteins recruited to ERα (IP-mass spec) in cultured endothelial cells in response to E2 and genes whose translation is upregulated by E2 in skeletal muscle microvascular endothelial cells in vivo (TRAPseq, *p* ≤ 0.05). **h**, **i** Pathway analyses of the intersection of proteins disassociated from ERα and genes whose translation is downregulated by E2 (**h**), and of the intersection of proteins recruited to ERα and genes whose translation is upregulated by E2 (**i**). Gene ontology (biological processes) gene sets are listed and fold enrichment ratios are shown for the top 20 pathways based on the false discovery rate ≤0.05. Source data are provided as a Source Data file.

To further investigate the basis by which ERα in endothelial cells promotes insulin transport, we leveraged the observation that non-nuclear and nuclear actions of the receptor are required, and queried both sets of processes using non-biased approaches. Since non-nuclear ERα function in endothelial cells requires dynamic receptor interaction with other proteins^50^, we employed immunoprecipitation (IP) and liquid chromatography/tandem mass spectrometry (LC/MS-MS) to interrogate the ERα interactome in endothelial cells and how it is influenced by E2 (Fig. 4a, Supplementary Fig 6a). E2 treatment for 30 min caused both the recruitment and the dissociation of numerous proteins from the receptor. Supplementary Table 1 lists the 449 proteins recruited to ERα and the 214 proteins dissociated from the receptor upon E2 liganding. Pathway analysis using biological process terms from Gene Ontology analysis^53^ revealed that a number of the proteins disassociated from the receptor mediate processes in leukocytes, whereas a number of the proteins recruited to ERα are involved in cell export (Supplementary Fig. 6b, c). The findings regarding the ERα interactome in endothelial cells were compared to 3 available reports for ERα interacting proteins in MCF-7 breast cancer cells (Supplementary Table 2). They interrogated the ERα interactomes in MCF-7 cells involving extra-nuclear proteins^54,55^ or nuclear proteins^56^. When the combined findings in these reports are compared to the present observations for proteins disassociated from ERα in response to E2 in endothelial cells, the commonality includes proteins involved in membrane localization (Supplementary Fig. 6d, e); for proteins recruited to the receptor with E2 treatment in endothelial cells, the common proteins are involved in cytoskeletal organization (Supplementary Fig. 6f, g).

To query the nuclear actions of the E2/ERα tandem that promote microvascular endothelial insulin transport in skeletal muscle, we interrogated the translatome in mouse skeletal muscle microvascular endothelial cells in vivo. Translating ribosome affinity purification (TRAP) followed by RNA-seq (TRAPseq) was performed in mice expressing a GFP-tagged version of the ribosomal protein L10a selectively in endothelial cells (GFP-L10a^EC^). To establish the approach, skeletal muscle (gastrocnemius and soleus) was harvested from the GFP-L10a^EC^ mice, total RNA was isolated from the muscle, and TRAP RNA was purified from the muscle endothelium by anti-GFP immunoprecipitation. Q-RT-PCR revealed that whereas RNAs for the myocyte-specific genes myogenin (MyoG), myosin D1 (MyoD1) and alpha-smooth muscle actin (α-SMA) were enriched in the whole tissue and not in the TRAP RNA (Fig. 4b), RNAs for the endothelium-specific genes roundabout (Robo), PECAM-1, and VECad were highly enriched in the TRAP RNA (Fig. 4c). E2 modulation of gene translation in the skeletal muscle microvascular endothelium was then interrogated by TRAPseq in the skeletal muscle of ovariectomized female GFP-L10a^EC^ mice treated with E2 for 4 weeks (Fig. 4d). As is shown in the volcano plot in Fig. 4e, the translation of a large number of genes was downregulated or upregulated in the skeletal muscle microvascular endothelium in response to E2 treatment. Genes demonstrating upregulated and downregulated translation are listed in Supplementary Table 3, along with their human homologs. TRAPseq on muscle microvascular endothelium will also be useful in other paradigms in mice in which muscle endothelial insulin transport is altered, such as in the setting of insulin resistance caused by diet-induced obesity^48^.

Next, we pursued the concept that genes upregulated by E2 whose gene products are recruited to E2-liganded ERα in endothelial cells may reveal those involved in the promotion of endothelial insulin transport, and merged the findings by IP-LC/MS-MS and TRAPseq. The overlap of E2-downregulated ERα-interacting proteins and transcripts identified 27 genes (Fig. 4f, h and Table 1), some of which are involved in mRNA processing. Conversely, with E2 treatment there were 23 proteins recruited to ERα whose gene translation was also increased by E2 (Fig. 4g, i, and Table 1). Interestingly these included a number involved in metabolic processes.

**Table 1:** Proteins disassociated from ERα whose gene translation is decreased, and proteins recruited to ERα whose gene translation is upregulated in response to E2

Disassociated proteins and downregulated genes	Recruited proteins and upregulated genes
Acsl3	Actg1
Asph	Arcn1
Col3a1	Arpc1a
Col4a2	C3
Ctnnb1	Eif2s3x
Cyb5r3	Gapdh
Dsp	Got1
Epha4	Gstp2
Gna11	Gyg
Hmgn5	Hspb1
Hsp90aa1	Mapk1
Immt	Pcna
Jup	Pebp1
Lmo7	Pgk1
Luc7l3	Prps1l1
Myh9	Psma5
Pex14	Rac2
Ppm1g	Slc25a12
Prpf31	Slc3a2
Prpf6	Snx5
Rbm39	Ssr3
Samm50	Tpi1
Snrnp200	Uqcrc2
Stt3b	
Sub1	
Tra2b	
Upf2	

### ERa promotes endothelial insulin transport via coupled nuclear and non-nuclear actions on SNX5

**Fig. 5:**
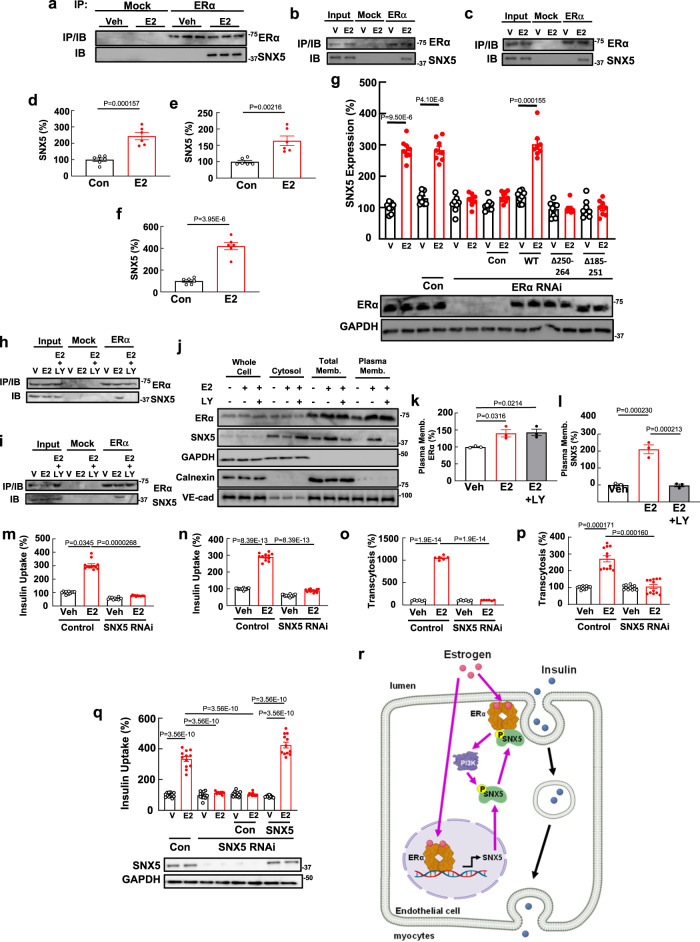
ERα promotion of endothelial insulin transport requires sorting nexin 5 (SNX5). **a** HAEC were treated with vehicle (Veh) or E2 and co-immunoprecipitation was performed with mock IgG or anti-ERα antibody to yield samples subjected to LC/MS-MS (*n* = 3). Immunoblots for ERα and SNX5 are shown. **b**, **c** E2 upregulation of ERα-SNX5 interaction in HAEC (**b**) and HSMEC (**c**). **d**, **e** E2 upregulation of SNX5 expression in HAEC (**d**) and HSMEC (**e**), with *n* = 6 wells/treatment. **f** E2 increases SNX5 gene translation in skeletal muscle endothelial cells in vivo in mice. *N* = 6 mice per treatment. **g** E2/ERα upregulation of SNX5 requires nuclear ERα action. E2 effect on SNX5 expression in HAEC was determined before and after ERα knockdown and reconstitution with empty vector control (Con), wild-type ERα (WT), ERαΔ250-274 or ERαΔ185-251. *N* = 8 wells/ group. Lower panel displays ERα knockdown and reconstitution by immunoblotting. **h**, **i** ERα interaction with SNX5 in HAEC (**h**) and HSMEC (**i**), and effect of E2 in the absence or presence of LY294002. **j**–**l** E2-stimulated, PI3 kinase-dependent recruitment of SNX5 to the plasma membrane. Representative immunoblots are shown (**j**), and plasma membrane-associated ERα and SNX5 abundance is expressed relative to VE-cadherin (**k** and **l**, respectively, *n* = 3 wells/treatment). **m**–**p** E2 stimulation of insulin uptake (**m**, **n**, *n* = 12 wells/group) and transcytosis (**o**, **p**, *n* = 6 and 12 wells/group, respectively) by HAEC (**m**, **o**) and HSMEC (**n**, **p**), in cells expressing versus lacking SNX5. **q** Effect of E2 on insulin uptake in HAEC following SNX5 knockdown and reconstitution with empty vector control (Con) or wild-type SNX5. *N* = 12 wells/group. Lower panel displays SNX5 knockdown and reconstitution by immunoblotting. Data are mean ± SEM, and *p* values by two-sided Student’s *t* test (**d**, **f**, **g**), Mann–Whitney testing (**e**, **g**), one-way ANOVA with Tukey’s post-hoc testing (**k**, **l**, **q**), and Kruskal–Wallis testing with FDR method of Benjamini and Hochberg (**m**) or Dunn’s post hoc testing (**n**–**p**) are shown. **r** E2 and ERα promote skeletal muscle microvascular endothelial cell insulin transcytosis through coupled nuclear and non-nuclear receptor actions on SNX5. The non-nuclear processes entail PI3 kinase-dependent recruitment of SNX5 to the plasma membrane and to ERα. Created by BioRender.com. Source data are provided as a Source Data file.

As highlighted in Fig. 4g, one of the 23 endothelial genes with increased translation by TRAPseq and increased gene product recruitment to ERα by LC/MS-MS in response to E2 was sorting nexin 5 (SNX5). SNX5 is a member of the SNX protein family of cytoplasmic and membrane-associated proteins that mediate vesicular trafficking of plasma membrane proteins^57–59^. Immunoblotting of the anti-ERα coimmunoprecipitated proteins from HAEC subjected to LC/MS-MS displayed SNX5 following cell treatment with E2 (Fig. 5a). Independent co-immunoprecipitation studies demonstrated parallel E2 promotion of ERα-SNX5 interaction in HAEC and HSMEC (Fig. 5b, c), and E2-induced upregulation of SNX5 expression was directly demonstrated in both HAEC and HSMEC (Fig. 5d, e). In addition, E2 enhancement of SNX5 gene translation in vivo in the skeletal muscle microvascular endothelium was shown by QPCR on TRAP RNA (Fig. 5f). To determine if nuclear actions of ERα mediate the upregulation of SNX5 expression by E2, experiments were performed in HAEC in which endogenous ERα was replaced with either wild-type ERα (WT) or two mutant forms of ERα with altered nuclear function (Fig. 5g). Whereas reconstitution with WT ERα caused a return of SNX5 upregulation in response to E2, upregulation did not occur in cells harboring mutant ERα lacking nuclear localization signals 2 and 3 (ERαΔ250-274) or the DNA binding domain (ERαΔ185-251)^60,61^. These findings are consistent with the observations made using the nonbiased approaches, confirming that the activation of ERα increases both SNX5 expression through nuclear actions of the receptor and SNX5 interaction with the receptor in endothelial cells.

SNX5 and related SNX family members contain a phosphoinositide-binding Phox (or PX) domain which binds PIP3 to mediate SNX5 localization to membranes^62^. Recognizing that E2 liganding of ERα in endothelial cells activates PI3 kinase^50^, and having found that E2 stimulation of endothelial cell insulin transport is PI3 kinase-dependent (Fig. 3b, c, e–g), we determined if PI3 kinase participates in the modulation of SNX5 in endothelium by the E2-ERα tandem. LY294002 treatment prevented E2 promotion of ERα-SNX5 interaction in both HAEC and HSMEC (Fig. 5h, i), in response to E2 both ERα and SNX5 were recruited to the plasma membrane, and the SNX5 trafficking required PI3 kinase (Fig. 5j–l). As such, the requirement for PI3 kinase stimulation in E2-related enhancement of endothelial insulin transport likely reflects the role of the kinase in SNX5 recruitment to the plasma membrane and to ERα. In addition, RNAi was employed to determine if there is a requirement for SNX5 in E2 stimulation of endothelial cell insulin transport in HAEC and HSMEC. Importantly, the knockdown of SNX5 did not alter insulin receptor or insulin receptor substrate-1 expression in endothelial cells, as was previously observed in renal proximal tubule cells (Supplementary Fig. 7a–c, e–g)^63^. Since insulin receptor substrate-2 in endothelial cells is critical to insulin transport^64^, its expression was also assessed and it was unchanged by SNX5 knockdown (Supplementary Fig. 7a, d, e, h). However, the loss of SNX5 fully attenuated E2-stimulated insulin uptake and transcytosis in both HAEC and HSMEC (Fig. 5m–p). Since insulin transcytosis requires its endocytosis, the combined findings likely indicate that the primary process modulated by the coupling of ERα to SNX5 is insulin endocytosis. Finally, SNX5 reconstitution studies demonstrated that the loss of E2-stimulated insulin uptake with SNX5 silencing is not due to off-target effects of SNX5 RNAi (Fig. 5q). Thus, SNX5 is a linchpin that couples ERα to endothelial cell insulin transport (Fig. 5r), providing the molecular underpinnings of the first demonstration of a steroid hormone receptor promoting a transcytotic process.

## Discussion

Serving as both a transcription factor and an initiator of non-nuclear signaling in response to estrogens^20,65^, it is well recognized that ERα in endothelial cells modulates angiogenesis and inflammation^26–28^. Since angiogenesis and inflammation greatly influence mechanisms in adipose tissue and adipose impact on glucose homeostasis^24,25^, in our interrogation of endothelial ERα impact on metabolism, it was surprising to find a lack of effect of endothelial ERα on adipose tissue. Instead, we discovered that endothelial ERα promotes glucose tolerance by enhancing insulin delivery to skeletal muscle and thereby increasing glucose disposal. We further found that this is not related to impact on insulin-induced capillary recruitment or blood flow in muscle, or changes in skeletal muscle capillary endothelium vesicle structure. Alternatively, the transcytosis of insulin across skeletal muscle endothelial cells is potently stimulated by E2 activation of endothelial ERα. The contribution of this process to the anti-diabetic properties of E2 is substantial, as endothelial ERα deletion fully negated the antidiabetic actions of the hormone in HFD-fed ovariectomized female mice.

Springboarding from the findings of non-biased interrogation of the ERα interactome and translatome in the endothelium, it was further revealed in cell culture that ERα governance of endothelial insulin transport requires coupled nuclear and non-nuclear actions on sorting nexin 5 (SNX5). SNX5 is a member of the SNX protein family of cytoplasmic and membrane-associated proteins that mediate vesicular trafficking of plasma membrane proteins^57–59^. We show that in order for the E2-ERα tandem to enhance endothelial insulin transport, ERα must serve as a transcription factor upregulating SNX5 expression and also as a means of PI3 kinase activation, which targets SNX5 to join ERα on the plasma membrane. We have determined that SNX5 knockdown attenuates both E2-stimulated insulin uptake and transcytosis. Since the process of transcytosis requires insulin uptake, these combined findings likely indicate that the SNX5-ERα complex regulates insulin endocytosis.

Similar to the SNX5-related mechanisms revealed in cell culture, there are now two sets of observations that begin to address the participation of both nuclear and non-nuclear processes involving ERα promotion of insulin sensitivity in vivo. In prior work, we demonstrated that non-nuclear ER activation alone with EDC in ovariectomized, HFD-fed female mice does not afford the protection from insulin resistance observed with E2 treatment^52^. In the present work, we demonstrate that the translation of the SNX5 gene is increased by E2 in the skeletal muscle microvascular endothelium in vivo (Fig. 5f). Thus, the nuclear ERα action found to be critical to endothelial insulin transport in culture is relevant in vivo, and non-nuclear ERα action is insufficient in vivo.

Along with deepening our understanding of the metabolic actions of estrogens, the present work identifies a physiologic process that promotes endothelial insulin transport to skeletal muscle to foster normal glucose homeostasis. The present discoveries further raise the possibility that therapeutic strategies can be developed which will combat type 2 diabetes by enhancing endothelial cell insulin transport, thereby increasing insulin delivery and ultimately glucose disposal in skeletal muscle.

## Methods

### Mouse models

Mice with endothelial cell-specific deletion of ERα (ERα^ΔEC^) were generated by crossing ERα floxed mice (ERα^fl/fl^)^66^ with vascular endothelial cadherin promoter-driven Cre mice (VECad-Cre)^29,67^. Cre-negative ERα^fl/fl^ mice served as controls. To assess the efficiency and cell-specificity of ERα deletion from endothelial cells in vivo, primary endothelial cells were cultured from mouse aorta as previously described^68^, and then further purified using rat anti-mouse CD31 (1:500, ThermoFisher cat. No. RM5200) and covalently bound sheep anti-rat IgG on magnetic beads (Dynabeads, ThermoFisher). Myeloid lineage cells were purified from bone marrow using biotinylated rat anti-mouse Mac-1 antibody (1:200, BD Biosciences cat. No. 557395), streptavidin particles (BD Biosciences), and Easysep kits (STEMCELL Technologies)^68^. RNA was isolated from the endothelial cells or myeloid lineage cells, and quantitative RT-PCR for mouse ERα was performed, expressing steady-state ERα transcript levels relative to mouse hypoxanthine guanine phosphoribosyl transferase (HPRT) endogenous control^52^, and then normalized to abundance in Cre negative ERα^fl/fl^ mice. Quantitative RT-PCR TaqMan assay information is provided in Supplementary Table 4.

Experiments were performed in both male and female mice. The mice were housed at 23 °C with light cycles of 12 h of light beginning at 6:00am and 12 h of dark beginning at 6:00 pm, humidity was 30-70%, and food and H_2_O were provided ad libitum. Beginning at 5 weeks of age, the males received a standard chow (Harlan Teklad 2016, 12% calories from fat) and the females received a high-fat diet (HFD; Harlan Teklad TD88137, 42% calories from fat, 0.2% cholesterol) for 12 weeks. The females also underwent ovariectomy at 6 weeks of age, at which time treatment was initiated with vehicle or E2 at 6 ug/d using subcutaneously implanted pellets (Innovative Research of America). Effective loss of endogenous estrogen action versus successful E2 delivery was confirmed at the time of euthanasia by measurement of uterine wet weights.

Additional experiments were performed in female mice to evaluate the effects of E2 on actively translated genes in the skeletal muscle microvascular endothelium. This was accomplished by crossing mice in which the Rosa26 locus has been targeted with a construct containing a floxed stop codon and GFP-*Rpl10a*, which encodes the ribosomal protein L10a with a GFP tag (Rosa26^fsTRAP^)^69^, with VECad-Cre mice to enact endothelial cell-specific expression of GFP-L10a (designated GFP-L10a^EC^). The female GFP-L10a^EC^ mice were ovariectomized at 6 weeks of age and treated with vehicle versus E2 pellets as described above for 4 weeks. All animals were treated and cared for in accordance with the Guide for the Care and Use of Laboratory Animals [National Institutes of Health (NIH), Revised 2011], and the Institutional Animal Care and Use Committee of the University of Texas Southwestern Medical Center approved all experiments.

### Evaluation of adiposity

Fat mass and lean body mass were determined by NMR (Minispec NMR Analyzer; Bruker). In select experiments, at the time of euthanasia subcutaneous white adipose tissue (WAT) was quantified by determining the weight of the inguinal fat pad, and visceral WAT was quantified by determining the weight of the gonadal fat pad^70^.

### Evaluation of glucose homeostasis

Following fasting for 4–6 h, glucose tolerance tests (GTT) and insulin tolerance tests (ITT) were performed in response to an IP injection with D-glucose (1 g/kg BW) or insulin (1unit/kg BW), respectively^71^. Tail vein blood samples were obtained at the indicated times for plasma glucose measurement by glucometer (ONE TOUCH Ultra2, Johnson & Johnson). In select studies, homeostasis model assessment of insulin resistance (HOMA-IR) was determined as previously described to assess relative insulin sensitivity^70^, or hyperinsulinemic-euglycemic clamps were performed using our established approach^48,71^. In brief, in the clamps hyperinsulinemia was initiated with a primed continuous infusion of insulin (20 mU/kg/min), while a variable infusion of 50% dextrose and monitoring of blood glucose every 5 min by glucometer allowed for the achievement of a targeted blood glucose level between 115-125 mg/dl. The glucose infusion rate was calculated. To evaluate pancreatic insulin secretion, mice fasted for 16 h were administered an IP injection of D-glucose (3 g/kg BW), and plasma samples were obtained at baseline and 2, 5, 15, and 30 min post-injection. Plasma insulin concentrations were determined by ELISA (Crystal Chem Inc. #90080 Ver. 15)^71^. To evaluate relative hepatic insulin sensitivity by assessing hepatic gluconeogenesis, pyruvate tolerance tests (PTT) were performed. Mice were fasted for 4 h, and changes in plasma glucose were evaluated over 120 min following an IP injection of pyruvate (2 g/kg body weight)^70^.

### Quantitative real-time PCR for adipose genes

RNA from inguinal (subcutaneous WAT) and gonadal (visceral WAT) fat pads was isolated using RNAzol RT reagent (Sigma) according to the manufacturer’s instructions, and cDNA was generated from 2 µg total RNA using the High Capacity RNA-to-cDNA Kit (Applied Biosystems). Flk1, PECAM-1, F4/80, CD11, IL-6, and TNFα gene expression was evaluated by quantitative RT-PCR as previously described (Supplementary Table 4)^72^. HPRT expression was assessed in parallel to indicate total RNA abundance, and relative expression was determined by the 2^−∆∆CT^ method and is reported normalized to the abundance of the transcript in the control group samples.

### Skeletal muscle glucose uptake, insulin signaling, and insulin delivery

Skeletal muscle glucose uptake was measured in vivo as previously reported^73,74^. Briefly, fasted mice were injected IP with 2-deoxy-[^3^H]glucose ([^3^H]-2-DOG, Amersham; 2 g/kg; 10 µCi/mouse) mixed with dextrose (20%), and blood glucose was measured at 0–90 min. The glucose-specific activity (disintegrations/min/µmol) was calculated by dividing the radioactivity by the glucose concentration, and the area under the curve (AUC) was integrated for the duration of the experiment (90 min). Skeletal muscle (soleus and gastrocnemius) was harvested at 90 min, and the specific accumulation of [^3^H]-2-DOG was calculated by dividing the radioactive counts (disintegrations/min) by the integrated glucose-specific activity (AUC) and the sample protein content (Bradford assay, Bio Rad Laboratories).

To evaluate skeletal muscle insulin delivery and signaling to Akt, fasted mice received tail vein injections with vehicle (saline) or bovine insulin (1 unit/kg BW), 5 min later the soleus and gastrocnemius were harvested and homogenized in PBS, and protein content was quantified (Bradford, BioRad). Phosphorylated Akt and total Akt were detected by immunoblotting using anti-phospho-Akt (S473,1:1000, Cell Signaling cat. No. 9271), anti-Akt (1:1000, Cell Signaling cat. No. 9272) and anti-GAPDH antibodies (1:2500, Santa Cruz Biotech cat. No. 47724). Insulin abundance was measured by ELISA (Crystal Chem Inc. cat. No. 90095). The ELISA detects both mouse and bovine insulin, with 2.1-fold greater sensitivity for bovine versus mouse insulin^48^.

To determine if the impact of endothelial ERα deletion on skeletal muscle glucose uptake entails processes limited to the endothelium, ex vivo studies of insulin-stimulated glucose uptake were performed in isolated soleus and extensor digitorum longus muscle using our previously established methods^71^. Briefly, the muscles were excised and incubated at 30 °C in the absence or presence of 2 nmol/L insulin for 40 min in Krebs-Henseleit buffer supplemented with 5 mmol/L Hepes, 0.1% BSA, and 2 mmol/L pyruvate. Muscles were further incubated for an additional 20 min in Krebs-Henseleit buffer containing 1 mmol/L [^3^H]2-DOG (2.5 mCi/mL) and 19 mmol/L [^14^C]mannitol (0.7 mCi/mL) at 30 °C. After incubation, muscles were digested in 0.5 mol/L NaOH, and extracellular space and intracellular 2-deoxyglucose concentrations were determined.

### Contrast-enhanced ultrasound (CEUS) of skeletal muscle microvasculature

CEUS was performed on the proximal hind limb adductor muscle group (adductor magnus and semimembranosus) to determine the microvascular response to a 2-hour hyperinsulinemic-euglycemic clamp entailing an insulin infusion at 20 mU/Kg/min and maintenance of blood glucose at 115–125 mg/dl as previously described^46,48^. In brief, under isoflurane anesthesia a right jugular vein catheter was placed, and following stabilization the muscle was imaged using a Siemens Sequoia ultrasound system equipped with a high-frequency 15L8 transducer and a microbubble (MB) contrast agent–sensitive imaging mode (cadence pulse sequencing, CPS) with a transmit frequency of 10 MHz. CEUS imaging was performed at baseline and at the end of the clamp, immediately before and for 10 min after a 1 min, constant rate-controlled i.v. infusion of a lipid-shelled perfluorocarbon-based MB contrast agent (Advanced Microbubbles Laboratories). The 1-min constant infusion avoids issues with microbubble floatation in the infusion syringe and catheter tubing during a longer infusion. Data were recorded and processed offline using prior methods^48^^.^ An automated region-of-interest (ROI) selection was performed which includes only the vessels detected by MB signal in the skeletal muscle depicted in the CEUS image after deleting the tissue scatter signal^47^. The ultrasound transducer was fixed for the entire study including the repeat in vivo ultrasound imaging sessions. After the selection of the initial ROI from the baseline imaging session, the same area was superimposed and maintained for all repeat quantifications. With this approach any increases in tissue perfusion are due to microvascular recruitment in response to the insulin clamp as a signal from the larger vessels is the same for each imaging session/time point. Average time-intensity curves were generated and analyzed to extract parametric measurements of capillary blood volume or CBV (peak intensity, *Ipk*), microvascular blood flow or MBF (time to peak intensity, *Tpk*; and wash-in rate, *WIR*), and both CBV and MBF (area under the curve, *AUC*; and wash-out rate, *WOR*)^43–48^.

### Electron microscopy of skeletal muscle capillary endothelium

Male mice (*n* = 6/group) were fed standard chow beginning at weaning, and at 17 weeks of age the gastrocnemius was harvested for electron microscopic analysis. Tissues were fixed in glutaraldehyde (2.5% in cacodylate buffer pH7.4) followed by osmium tetroxide (1% in cacodylate buffer). Six capillaries were imaged per mouse using a JEOL 1400 Plus electron micrograph. Vesicles were designated as associated with the luminal plasma membrane or the abluminal plasma membrane, or localized intracellularly. The number of vesicles was manually counted and divided by the entire area of the endothelium. For the assessment of vesicle area, three randomly chosen regions were selected per capillary (6 capillaries/mouse, a total of 18 areas/mouse), and the area of each vesicle was measured manually using Image J.

### Endothelial cell insulin transport and gene expression

Human aortic endothelial cells (HAEC) were purchased from Lonza and maintained in EGM-2 and Endothelial Growth Medium with added growth factors (Lonza). Human skeletal muscle endothelial cells (HSMEC) were obtained from Cell Biologics and were maintained in Complete Human Endothelial Cell Medium with added growth factors (Cell Biologics). Cells were used within 3 to 6 passages. To strengthen the parallel observations in HSMEC and in vivo in mice, select experiments were performed using primary microvascular endothelial cells isolated from the skeletal muscles (soleus and EDL) of 5-6 week-old male ERα^fl/fl^ and ERα^ΔEC^ mice. Briefly, following the dissociation of the tissue with Collagenase IV (0.2% (w/v) and Dispase (1.25 U/ml), the cell pellet was treated to lyse red blood cells, CD31 MicroBeads (Miltenyi Biotech) were added to resuspended cells, and the CD31+ fraction was isolated using a magnetic separator^75^.

Prior to the study of insulin transport the cells were placed in media lacking growth factors with 1% FBS added for 18 h. To assess insulin uptake the cells were placed in media containing vehicle or E2 (10^−8^ M) and FITC-conjugated insulin (5 × 10^−5^ M, Sigma-Aldrich) for 30 min at 37 °C and immunofluoresence was detected by microscopy (NIKON Eclipse TE2000, ×20 magnification) or by POLARstar Omega plate reader (BMG LABTECH). To evaluate the participation of ERα or PI3 kinase, the cells were treated with vehicle or MPP (10^−5^ M) or LY294002 (5 × 10^−5^ M) during a 120 min preincubation at 37 °C and during the incubations with FITC-insulin. In select experiments, the requirement for SNX5 in E2-stimulated insulin uptake was interrogated in HAEC and HSMEC. Cells were transfected with 5 nM control siRNA (Dharmacon, cat. No. D-001810-02-20) or siRNA targeting SX5 (Silencer Select, ThermoFisher cat. No. 439240), and insulin uptake was studied 24 h later. Effective knockdown of SX5 was confirmed by immunoblotting (1:1000, Abcam cat. No. ab5983). To assess effects of SNX5 on other key participants in endothelial insulin transport, additional experiments in both HMEC and HSMEC evaluated how SNX5 knockdown impacted IR, IRS-1 and IRS-2 expression using immunoblotting (1:1000, Cell Signaling, cat. No. 234135, 3407T, and 4502, respectively). To exclude off-target effects of SNX5 RNAi, SNX5 expression was reconstituted in HAEC in which siRNA knockdown was previously performed. The siRNA knockdown proceeded for 24 h and the next day the cells were transfected with concentrated lentiviral particles (Lenti-X Concentrator TAKARA, cat#631232) encoding either eGFP control or SNX5. E2-stimulated insulin uptake was tested 18 h later.

Insulin transcytosis was studied as previously described^68^. Cells were seeded onto Transwell inserts (6.5 mm diameter, 3 µm pore size, polycarbonate membrane inserts, Sigma Aldrich) treated with 100ug/ml collagen I (BD Bioscience), and transendothelial electrical resistance (TEER) was monitored daily for 4-6d until studies were performed using an epithelial volt-ohmmeter (EVOM, World Precision Instruments) to confirm the establishment of a confluent monolayer^51^. FITC-insulin (5 × 10^−8^ M, Sigma-Aldrich) was introduced into the upper chamber along with vehicle or E2(10^−8^ M), and the cells were incubated for 30 min at 37 °C. At the end of the incubation, the FITC-insulin in the lower chamber was quantified using a fluorimeter (POLARstar Omega, BMG LABTECH), and the percentage of insulin initially placed in the upper chamber transported to the bottom chamber was calculated. Incubations with FITC-dextran (M.W. 4000, Sigma Aldrich, 60 min) were then performed, and less than 5% of FITC-dextran added to the upper chamber was detected in the lower chamber indicating negligible paracellular transport. The stimulation of FITC-insulin transcytosis by E2 was decreased by 84% by the addition of excess unlabeled insulin (5 × 10^−4^ M), revealing insulin receptor dependence and confirming that the process entails transcytosis. The participation of ERα or PI3 kinase in insulin transcytosis was evaluated using preincubation and incubation with vehicle or MPP or LY294002 as described for the insulin uptake studies. In select insulin transcytosis studies the effects of E2 were compared to those of the estrogen dendrimer conjugate (EDC), which selectively activates non-nuclear estrogen receptors^26^. Cells were treated with vehicle, E2 (10^−8^ M), dendrimer control at a concentration equivalent to EDC, or EDC providing tethered E2 at a concentration equivalent to 10^−8^ M free E2. In additional experiments insulin transcytosis was evaluated in cells infected with a control lentiviral construct versus a lentivirus harboring shRNA targeting SNX5. Effective shRNA-based knockdown of SNX5 was confirmed by immunoblotting.

To investigate the mechanism by which ERα modulates SNX5 expression, the receptor was silenced in HAEC by RNAi (Silencer Select, ThermoFisher cat. No. 4392420) confirming effective knockdown by immunoblotting, and in select samples ERα expression was reconstituted with either wild-type ERα (WT), mutant ERα lacking nuclear localization signals 2 and 3 (ERαΔ250-274) or mutant ERα lacking the DNA binding domain (ERαΔ185-251)^60,61^. Similar to what is described above for SNX5 reconstitution, following siRNA knockdown of ERα the cells were transfected with lentiviruses encoding eGFP control, WT ERα, ERαΔ250-274 or ERαΔ185-251, and 18 h later E2 stimulation of SNX5 expression uptake was tested. Effective ERα knockdown and reconstitution were confirmed by immunoblotting (1:1000, SantaCruz Biotech. cat. No. sc-8002).

### Preparation of estrogen dendrimer conjugate (EDC)

EDC was prepared according to prior methods^76–78^. Briefly, a benzaldehyde derivative of 17α-ethinylestradiol was attached onto the primary amine groups of a G-6 poly(amido)amine (PAMAM) dendrimer by reductive amination with sodium borohydride in methanol. Small molecule reactants and products were removed by repeated (4 times) ultrafiltration through an Amicon centrifugal filter (Ultra-15, cutoff 30 K) using methanol. The composition of the EDC (average number of estradiol units per PAMAM dendrimer) was determined by Matrix-assisted laser desorption ionization time of flight (Maldi-TOF) mass spectrometry. For the preparation used in this study, there were an average of 20 estradiol molecules per molecule of dendrimer, with a polydispersity index of 1.02. The PAMAM dendrimer G6 control (DEN) was simply the G-6 PAMAM without conjugated estradiol molecules.

### Co-immunoprecipitation, plasma membrane recruitment, immunoblot analyses

Following previously described methods^79^, in co-immunoprecipitation experiments cells were treated with vehicle versus E2 (10^−8^M) for 30 min and then lysed with ice-cold buffer containing 1% Triton X-100, 100 mM NaCl_2_, 150 mM Tris-HCl, 1 mM CaCl_2_, 1 mM MgCl_2_ and protease inhibitors (Roche Diagnostics) at pH 8.0. Cell lysates were placed on ice for 15 min and then centrifuged at 18,000 × *g* for 10 min at 4 °C, and supernatants were incubated with protein A beads coated with anti-ERα antibody (F-10, Santa Cruz Biotech) or matching IgG subclass mock for 3 h at 4 °C with continued rotation. Beads were washed three times and immunoprecipitated complexes were extracted and analyzed by immunoblot analysis or by mass spectrometry. The immunoblot analyses, which included samples of cell lysates representing the co-immunoprecipitation inputs, were performed for ERα and SNX5.

In studies of plasma membrane targeting, HAEC were treated 5 min with vehicle, E2 (10^−8^M), or E2 preceded by pretreatment with LY294002 (5 × 10^−5^ M, 60 min), and plasma membranes were isolated from total cellular membranes by phase separation (Abcam plasma membrane extraction kit; ab65400). Following treatment, the cells were washed with ice cold PBS and pelleted twice, and then homogenized with an ice-cold Dounce apparatus. The resulting whole cell lysate was centrifuged at 700 × *g* for 10 min at 4 °C to pellet nuclei and cell debris, and the supernatant was centrifuged at 10,000 × *g* for 30 min at 4 °C to yield a cytosol fraction (supernatant) and total cellular membranes (pellet). The total membrane pellet was subjected to phase separation twice with centrifugation at 1000 × *g* for 5 min at 4 °C, the yield of plasma membrane was increased by sample dilution with 5 volumes of water and overnight incubation at 4 °C, and the final plasma membrane pellet was obtained by centrifugation at 21,000 × *g* for 10 min at 4 °C. The resulting cellular sub-fractions were immunoblotted for GAPDH (marker for cytosol), calnexin (marker for total membranes; 1:1000, Santa Cruz Biotech. cat. No. sc-23954), VE-cadherin (marker for plasma membrane; 1:1000, Santa Cruz Biotech. cat. No. sc-9989), ERα and SNX5.

### Liquid chromatography/tandem mass spectrometry (LC/MS-MS)

To evaluate changes in the ERα interactome in endothelial cells (HAEC) treated with vehicle versus E2 (10^−8^ M for 30 min), ERα was immunoprecipitated including mock IgG controls, and the associated proteins were evaluated by liquid chromatography/tandem mass spectrometry (LC/MS-MS)^79^. Three biological replicates were used for each condition. Following protein separation by sodium dodecyl sulfate polyacrylamide gel electrophoresis, gel samples were digested overnight with trypsin (Pierce) followed by reduction and alkylation with dithiothreitol and iodoacetamide (Sigma-Aldrich). After solid-phase extraction cleanup with Oasis HLB plates (Waters), the samples were analyzed by LC/MS-MS using an Orbitrap Fusion Lumos mass spectrometer (Thermo Electron) coupled to an Ultimate 3000 RSLC-Nano liquid chromatography system (Dionex). Samples were injected onto a 75 um i.d., 75-cm long EasySpray column (Thermo) and eluted with a gradient from 0 to 28% buffer B over 90 min. Buffer A contained 2% (v/v) ACN and 0.1% formic acid in water, and buffer B contained 80% (v/v) ACN, 10% (v/v) trifluoroethanol, and 0.1% formic acid in water. The mass spectrometer operated in positive ion mode with a source voltage of 1.5 kV and an ion transfer tube temperature of 275 °C. MS scans were acquired at 120,000 resolution in the Orbitrap and up to 10 MS/MS spectra were obtained in the ion trap for each full spectrum acquired using higher-energy collisional dissociation (HCD) for ions with charges 2-7. Dynamic exclusion was set for 25 s after an ion was selected for fragmentation. Raw MS data files were analyzed using Proteome Discoverer v2.4 SP1 (Thermo), with peptide identification performed using Sequest HT searching against the human-reviewed protein database from UniProt (downloaded April 8, 2022, 20361 entries). Fragment and precursor tolerances of 10 ppm and 0.6 Da were specified, and three missed cleavages were allowed. Carbamidomethylation of Cys was set as a fixed modification, with oxidation of Met set as a variable modification. The false-discovery rate (FDR) cutoff was 1% for all peptides.

The statistical analysis of the mass spectrometry data was performed using the Differential Enrichment analysis of Proteomics data (DEP)^80^ analysis workflow package (version 1.12.0), R version 4.0.2. Proteins found in 2 out of 3 replicates per condition were filtered for further downstream analysis. Variance Stabilizing Normalization was performed to normalize the intensities of the filtered proteins followed by “MinProb” based missing value imputation. Differential expression testing of conditions was performed using limma empirical Bayes statistics by constructing linear models for proteins. FDR values were higher than expected, primarily due to variability between samples. Therefore, for significance (*P* < 0.05) in proteomics results we employed non-adjusted *P* values. To further identify the proteins of interest, we only filtered proteins significantly changed with anti-ERα + E2 treatment compared to anti-ERα + vehicle treatment but not significantly changed in the comparison between Mock IgG + E2 treatment vs Mock IgG + vehicle treatment.

### In vivo endothelial cell TRAPseq

Active gene translation was evaluated in the skeletal muscle microvascular endothelial cells of female GFP-L10a^EC^ mice treated with vehicle versus E2 subcutaneous pellets post-ovariectomy for 4 weeks. Skeletal muscle (gastrocnemius and soleus) was harvested and ribosome-bound RNA from the muscle endothelium (TRAP RNA) was obtained by anti-GFP immunoprecipitation for 3 h at 4 °C using methods modified from those previously described^69^. There were 6 biological replicates per study group. RNAseq was then performed employing the TRAP RNA by the UT Southwestern McDermott Center Sequencing Core. Briefly, using the ARTseq/TruSeq Ribo Profile Library Preparation Kit (Illumina), RNA-seq libraries were prepared from 500 ng of ribosome-associated RNA. Effective library preparation was validated on a 2100 Bioanalyzer (Agilent Technologies), and after normalization, 30-40 million reads per library was sequenced on an Illumina NextSeq 500 using 150 nucleotide paired-end chemistry. Image intensities were processed using NextSeq 500 Control Software (Illumina) with default settings. Raw data were de-multiplexed and converted to fastq files using bcl2fastq (v2.17). The fastq files were checked for quality using fastqc (v0.11.2) (http://www.bioinformatics.babraham.ac.uk/projects/fastqc) and fastq_screen (v0.4.4) (http://www.bioinformatics.babraham.ac.uk/projects/fastq_screen). Sequencing reads were mapped to mm10 reference genome (from igenomes) using STAR^81^, counted using featureCounts^82^ and normalized using trimmed mean of M values (TMM) methods. Differential expression analysis was performed using edgeR (version 3.12)^83^.

Functional pathway analysis was performed using the ShinyGo application at ShinyGO 0.77 (sdstate.edu)^84^ with biological process terms from Biological Processes of Gene Ontology^53^. For the overlap with proteomics data with TRAPseq data, we first utilized the databases of Homologene (https://www.ncbi.nlm.nih.gov/homologene) and MGI homology (http://www.informatics.jax.org/homology.shtml) to convert the TRAPSeq mouse data to human homologs. Venn diagrams were generated for the overlapping genes between LC-MS/MS (FDR < 0.01) and human homologs of the TRAPseq differentially expressed genes (*p* $\le$ 0.05 and log_2_(counts per million) $\ge$ 0) (http://bioinformatics.psb.ugent.be/webtools/Venn/).

### Quanitative RT-PCR for TRAPseq

To evaluate the effective isolation of endothelial cell ribosomes from mouse skeletal muscle in the TRAPseq protocol, Q-RT-PCR was performed using established techniques^85^ on whole tissue RNA and endothelial TRAP RNA to detect the myocyte-specific genes myogenin (MyoG), -myosin D1 (MyoD1), and alpha-smooth muscle actin (alphaSMA) (α-SMA), and the endothelium-specific genes roundabout 4 (Robo4), PECAM-1, and VE-Cadherin (Supplementary Table 4). In both tissues and TRAP RNA the transcript abundance was determined relative to the housekeeping gene hypoxanthine-guanine phosphoribosyltransferase (HPRT). In experiments in cultured endothelial cells similar methods were employed to evaluate relative SNX5 transcript abundance relative to HPRT mRNA abundance.

### Statistical analysis and reproducibility

Following normality testing by Shapiro–Wilk test, for normally distributed datasets comparisons between two groups were performed by two-sided Student’s *t* tests, and differences between more than two groups were evaluated by one-way analysis of variance (ANOVA) with Tukey’s post-hoc testing, or by two-way ANOVA with Sidak’s post-hoc testing. In the instances in which normality testing failed, non-parametric analyses were performed between two non-paired groups using Mann–Whitney, between two paired groups using Wilcoxon matched-pairs signed rank test, and between more than two groups by Kruskal–Wallis with Dunn’s post-hoc testing. Findings in cell culture experiments were replicated in two independent experiments. Values shown are mean ± SEM. Significance was accepted at the 0.05 level of probability.

### Reporting summary

Further information on research design is available in the Nature Portfolio Reporting Summary linked to this article.

### Supplementary information

Supplementary Information

Description of Additional Supplementary Files

Supplementary Movie 1

Supplementary Movie 2

Reporting Summary

### Source data

Source Data

## Data Availability

The raw LC/MS-MS data in this study has been uploaded to the MassIVE data repository with accession number MSV000091095. The raw TRAPseq data in this study were deposited in GEO under record GSE179737. There are no restrictions on data availability. All differential proteomics data and integrated LC/ MS-MS and TRAPSeq analysis results are included in the Tables provided. Source data are provided with this paper.
