# Supplementary material for: Endothelial ERα promotes glucose tolerance by enhancing endothelial insulin transport to skeletal muscle
Source: Nat Commun. 2023 Aug 17;14:4989. doi: 10.1038/s41467-023-40562-w (PMC10435471; doi:10.1038/s41467-023-40562-w)
Supplement: Supplementary file 1 — Supplementary Information [file 41467_2023_40562_MOESM1_ESM.pdf]

Supplementary Table 1: Proteins dissociated from ERα and proteins recruited to the receptor upon E2 liganding.

Disassociated Proteins	Recruited Proteins
ABCA1	A1BG
ABCD3	A2M
ABLIM3	AARS1
ACAA2	ABHD5
ACADVL	ACTB
ACOT9	ACTC1
ACSL1	ACTG1
ACSL3	ACTN1
ADPGK	ACTN4
ALDH18A1	ACTR2
ARG1	ACTR3
ASAP2	ADK
ASPH	ADSS2
ATF6B	AFM
ATP13A1	AHSA1
ATP2A2	AHSG
ATP2C1	AIMP2
ATP5F1A	AK1
AZGP1	AKR1B1
BAZ1B	AKR1C3
BICC1	ALAD
BOD1L1	ALCAM
CAB39	ALDOC
CASP14	AMBP
CAVIN2	ANP32A
CCDC137	ANP32B
CCDC68	ANPEP
CDSN	ANXA2
CFAP46	ANXA3
CKAP4	ANXA5
CLTC	ANXA6
COL1A1	AOC3
COL2A1	AP1M1
COL3A1	AP2A2
COL4A2	APOA1
COL6A2	APOH
COL8A1	ARCN1
CSNK2B	ARF3
CTNNB1	ARF5
CYB5R3	ARHGDIB
DCD	ARL3
DDX41	ARMT1

DECR2	ARPC1A
DHCR7	ARPC1B
DHX29	ARPC2
DLAT	ARPC3
DLST	ARPC5
DSC1	ARPC5L
DSG1	ASNS
DSP	ATP1B3
DYSF	ATP5F1D
ECHS1	ATP5MF
EFEMP1	ATP5PD
EPHA4	ATP6V1B2
ERLIN2	B2M
ESYT1	BCAT1
ESYT2	BLVRA
EXD2	BSG
F11	BST1
F13A1	BTF3
FBN2	C3
FGB	C4A
FGG	C7
FKBP8	CACNA2D1
FLG	CACYBP
FLG2	CALM3
FN1	CALR
GATD3B	CALU
GIMAP5	CAND1
GNA11	CAP1
GPD2	CAPG
GPRIN1	CAPZB
GRN	CASP3
HADHA	CBR1
HADHB	CD47
HMGN5	CD59
HRNR	CDC37
HSD17B12	CDH13
HSD17B4	CDH2
HSP90AA1	CDV3
HSPA9	CFAP100
HSPG2	CFH
IDH3B	CFI
IGHG4	CFL1
IGHV1-45	CFL2
IGHV3-21	CHTOP
IGHV3-23	CLEC3B
IGHV6-1	CLIC1

IGHV8-51-1	CLIC3
IGKC	CLIC4
IGKV2-29	CMPK1
IGKV3-7	COPS2
IGKV4-1	CORO1B
IGKV6D-41	CORO1C
IGLL5	CORO7
IMMT	COTL1
ING3	COX4I1
JAGN1	CP
JUP	CPO
KDSR	CPPED1
KIF13A	CRIP2
KIFBP	CRK
KLHL22	CSTB
KPRP	CTSB
KRT1	CTSD
KRT10	CUL4B
KRT14	CYBRD1
KRT16	CYCS
KRT17	DARS1
KRT2	DBNL
KRT27	DDAH1
KRT5	DDB1
KRT6A	DDX58
KRT6B	DNAJB11
KRT71	DPP3
KRT77	DPYSL2
KRT78	DYNC1I2
KRT80	DYNC1LI1
KRT83	DYNC1LI2
KRT87P	DYNLL1
KRT9	ECH1
LETM1	EEF1D
LMF2	EEF1G
LMO7	EEF2
LRPPRC	EFL1
LUC7L3	EHD1
LYZ	EIF2AK2
MAML1	EIF2S1
METAP1	EIF2S2
MIDN	EIF2S3
MMRN1	EIF4A2
MOGS	EIF5
MYH9	EMILIN1
MYO1C	ENO1

NCCRP1	ESD
NDUFA6	F9
NIFK	FAH
OGDH	FAM114A1
OSBPL8	FAM74A7
PACS1	FASN
PAIP1	FBLN1
PANK1	FIS1
PCDH20	FKBP10
PCMTD1	FKBP11
PDE4C	FKBP1A
PDE8B	FSCN1
PEX14	FSTL1
PHYH	FTH1
PIKFYVE	G3BP1
PIP	GALNT2
PITRM1	GAPDH
POLR3F	GART
POR	GBE1
PPM1G	GC
PRPF3	GDI2
PRPF31	GFI1
PRPF40A	GFPT1
PRPF4B	GLO1
PRPF6	GLOD4
PRPF8	GLRX3
PTCD3	GMPS
PUF60	GNS
RAB32	GOLT1B
RAB5B	GORASP2
RAD23B	GOT1
RASGEF1A	GSN
RB1	GSS
RBM39	GSTM3
RDH11	GSTO1
RMDN3	GSTP1
RPL34	GYG1
RPL36A	HAUS4
RRP12	HCLS1
S100A11	HEBP1
S100A9	HEXB
SACM1L	HGFAC
SAMM50	HNRNPA1
SEC24B	HNRNPA2B1
SERPINB12	HNRNPAB
SERPINB3	HPCAL1

SESTD1	HPX
SGPL1	HSP90AB1
SLC12A4	HSPA4
SMARCA5	HSPB1
SNAP47	HSPE1
SNRNP200	HSPH1
SPG11	IDH1
SPRR1B	IFITM2
SPRR2D	IGF2
SRRM2	IGFBP7
SRSF7	IGHG1
SSRP1	IMPA1
STAT2	IPO5
STAU1	IPO7
STT3B	IPO9
STX18	ITGA3
SUB1	ITGA5
TACC1	ITGB1
TADA2B	ITIH1
TCAF1	ITIH2
TECR	ITIH3
TGFB1	ITIH4
TGM1	KARS1
TGM3	KCTD12
TMEM30A	KIF5B
TMX2	KNG1
TRA2B	KPNA2
TRPV2	KPNA3
UPF2	LAMP2
USH2A	LAP3
WDR18	LILRB4
WDR91	LIMS2
XP32	LIN54
ZW10	LPXN
	LRRC47
	LSS
	LTF
	LUC7L2
	LUM
	LXN
	MAGOH
	MAPK1
	MAT2A
	MAT2B
	MCAM
	MCTS1

	MDH1
	MDH2
	MGLL
	MIF
	MSN
	MT2A
	MT-CO2
	MTDH
	MTPN
	MYDGF
	NAA15
	NACA
	NADK2
	NAE1
	NAGA
	NAGK
	NANS
	NAP1L4
	NARS1
	NDUFB9
	NEDD8
	NIBAN2
	NIPSNAP3A
	NME1
	NME2
	NNMT
	NPEPPS
	NQO1
	NSA2
	NSF
	NSFL1C
	NUCB2
	NUDC
	NUP43
	OTUB1
	OXSR1
	PA2G4
	PABPN1
	PAPSS2
	PARVA
	PCNA
	PCYOX1
	PDIA3
	PDIA4
	PDIA6
	PEA15

	PEBP1
	PECAM1
	PEPD
	PGAM1
	PGK1
	PGLS
	PGM1
	PGM2
	PGM3
	PHB2
	PIR
	PKD1
	PKM
	PLS3
	PMM2
	PNP
	PODXL
	POLR2H
	PON1
	PPA1
	PPA2
	PPIA
	PPIB
	PPP2CB
	PRCP
	PRDX3
	PRDX6
	PRKAR1A
	PRKCSH
	PROS1
	PRPS1L1
	PSAP
	PSAT1
	PSMA2
	PSMA3
	PSMA5
	PSMA6
	PSMB2
	PSMB3
	PSMB4
	PSMB6
	PSMB7
	PSMD4
	PSMD8
	PSMD9
	PSME1

	PTGES3
	PTGR1
	PTTG1IP
	PXN
	PZP
	RAC1
	RAC2
	RAD23A
	RAE1
	RALA
	RAN
	RANBP1
	RBBP7
	RBM3
	RBP4
	RCN1
	RCN3
	REEP5
	RHEB
	RHOA
	RNH1
	RNPEP
	RO60
	RPS12
	RPS21
	RTCA
	RTN3
	S100A10
	S100A6
	SARS1
	SBF1
	SCARB2
	SCRN1
	SDCBP
	SEC11A
	SERBP1
	SERPINA7
	SERPINB6
	SERPINB9
	SERPINC1
	SERPINF1
	SERPINF2
	SERPING1
	SFXN1
	SFXN3
	SH3BGRL

	SH3BGRL3
	SKIV2L
	SKP1
	SLC16A3
	SLC1A5
	SLC25A12
	SLC3A2
	SNX5
	SOD1
	SOD2
	SPARC
	SRM
	SRP14
	SRP9
	SRPK1
	SSR3
	STIP1
	STRAP
	SULT1A1
	SULT1B1
	SWAP70
	TAGLN
	TAGLN2
	TARS1
	TBCB
	TDRD15
	TECPR2
	TES
	TF
	TFRC
	TMED10
	TMED2
	TMED9
	TMOD3
	TMSB4X
	TP53I3
	TPI1
	TPM1
	TPT1
	TRIM66
	TUBB
	TUBB1
	TUBB3
	TUBB6
	TWF1
	TWF2

	TXNDC12
	TXNDC17
	TXNDC5
	TXNL1
	TXNRD1
	UAP1
	UBA1
	UBA3
	UBE2D3
	UBE2N
	UBE2V1
	UBXN1
	UCHL1
	UFD1
	UFM1
	UGDH
	UGGT1
	UGP2
	UNC45A
	UQCRC1
	UQCRC2
	UROD
	USP14
	USP5
	VAMP7
	VCL
	VDAC1
	VPS4B
	WARS1
	WASF2
	WDFY1
	XPNPEP1
	XPO1
	YWHAB
	YWHAE
	YWHAQ
	YWHAZ
	ZIK1
	ZNF185

Supplementary Table 2: Proteins dissociated from or recruited to ERα upon E2 treatment of endothelial cells compared to ERα interacting proteins in MCF-7 cells.

Dissociated Proteins Shared with MCF-7 Cells	Recruited Proteins Shared with MCF-7 Cells
ABCD3	ACTB
ALDH18A1	ACTC1
ASPH	ACTN1
ATP13A1	ACTN4
CCDC137	ACTR2
CLTC	ANP32A
CTNNB1	ANXA2
DECR2	AP2A2
DHX29	APOH
DSP	ARPC1B
ESYT1	ARPC2
ESYT2	ARPC5
EXD2	ATP6V1B2
FKBP8	BTF3
HADHB	CACYBP
HSP90AA1	CAND1
HSPA9	CAPZB
IGLL5	CD59
IMMT	CDC37
JUP	CFL1
KIF13A	CLIC1
KRT78	CORO1C
METAP1	COX4I1
MYH9	DBNL
MYO1C	DNAJB11
OSBPL8	EEF1G
PRPF6	EEF2
PTCD3	EIF2AK2
RAD23B	EIF2S1
RBM39	EIF2S2
RMDN3	EIF5
RPL34	FASN
RPL36A	G3BP1
RRP12	GAPDH
SGPL1	GORASP2
SSRP1	GSN
STAU1	GSTM3
STT3B	HNRNPA1
STX18	HNRNPAB
TECR	HSP90AB1
TMX2	HSPA4

WDR18	HSPB1
	HSPH1
	KPNA3
	LAMP2
	MIF
	MTDH
	NACA
	NDUFB9
	NSF
	OTUB1
	PA2G4
	PDIA3
	PDIA6
	PHB2
	PLS3
	PPIB
	PRDX3
	PRDX6
	PRKAR1A
	PRKCSH
	PTGES3
	PXN
	RAC1
	RAC2
	RHOA
	RNH1
	RPS12
	RPS21
	S100A10
	SDCBP
	SERBP1
	SERPINF1
	SLC3A2
	SRPK1
	TFRC
	TMED10
	TMED2
	TMOD3
	TPM1
	TUBB
	TUBB3
	TUBB6
	TWF1
	UQCRC1
	UQCRC2
	WASF2
	YWHAB

	YWHAE
	YWHAQ
	YWHAZ

Supplementary Table 3: Mouse genes demonstrating downregulated and upregulated translation with E2 treatment by TRAP-Seq, and those with human homologs.

TRAPSEQ downregulated_mouse genes	Downregulated mouse genes with human homologs	TRAPSEQ upregulated mouse genes	Upregulated mouse genes with human homologs
Cdkn1c	CDKN1C	Gm12505	ADARB2
Sema3g	SEMA3G	4931440P22Rik	UBD
Rnase1	RNASE1	B430319G15Rik	CMAHP
Casp9	CASP9	Adarb2	CPM
Cpa1	CPA1	Ubd	EIF3I
Fnip2	FNIP2	E030024N20Rik	TCEAL5
Eif2s3y	HOXA7	Cmah	RPL28
Hoxa7	PTMS	Cpm	AURKAIP1
Ptms	FAM43A	Eif3i	XIST
Fam43a	CPB1	Tceal5	ELP5
Cpb1	TSEN34	Gm17821	TGM4
Tsen34	FAM212A	Rpl28	TMEM158
Fam212a	SAFB	Aurkaip1	SEMA4B
Safb	HIP1R	Xist	H3-3A
Hip1r	PHF14	6230400D17Rik	URGCP
Phf14	PRSS1	C330021F23Rik	MROH8
Prss2	NDUFAF4	D730005E14Rik	MRRF
Ndufaf4	CYB5B	Gm4944	RPL27
Cyb5b	CLTA	9130221H12Rik	RPS26
Clta	SDC4	AV051173	C3
Sdc4	CXCL12	A530088E08Rik	TVP23A
Cxcl12	PDGFA	Elp5	RMND5B
Pdgfa	CIDEA	4933408J17Rik	SNX5
Cidea	RPLP2	Gm5148	TADA1
Rplp2	SF3B2	4930414L22Rik	RPL6
Sf3b2	PNLIP	Tgm4	MICAL2
Pnlip	ACADSB	Tmem158	GMPPB
Acadsb	MRPL15	Sema4b	MYC
Mrpl15	HNRNPUL2	H3f3b	RPS15A
Hnrnpul2	C1orf115	Urgcp	CCDC106

C130074G19Rik	SLFN12	Mroh8	RPS7
Slfn3	TTLL7	4931431C16Rik	S100A4
Ttll7	COL4A2	Mrrf	ARHGAP26
Diap2	SMAP1	Rpl27	HOXA11-AS
Col4a2	HNRNPH3	Rps26	RPS6
Smap1	TLK2	C3	SLC4A4
Hnrnph3	PNLIPRP1	Tomm6os	MROH2A
Tlk2	PALMD	Tvp23a	MED16
Pnliprp1	PGAM2	Rmnd5b	GTF2H5
Palmd	PKP4	Snx5	LIMD2
Pgam2	CWC25	Tada1	PKD2L2
Pkp4	HNRNPU	Rpl6	UNC5C
Cwc25	FAM50A	Mical2	DUT
Hnrnpu	GRK1	Rbm3os	ENDOG
Fam50a	ENAH	Gmppb	UQCRC2
Grk1	SUB1	Myc	C22orf39
Enah	CAMK2N1	6330410L21Rik	IL7
Sub1	CCDC85A	Rps15a	PPP2R5D
Camk2n1	BBX	Ccdc106	FNDC9
Ccdc85a	SMOX	Rps7	ACTA1
Bbx	DROSHA	S100a4	RPL18
Smox	MCC	Arhgap26	ANKS3
Drosha	ARHGEF5	Hoxa11os	WDR70
Mcc	OSBPL1A	Rps6	HDAC3
Arhgef5	CHTOP	5031425E22Rik	RPL8
Osbpl1a	RAMP1	Slc4a4	SLFN11
Chtop	LPAR1	Mroh2a	NRG4
Gm7120	CBX1	Med16	GPER1
Ramp1	FYTTD1	Gtf2h5	GABPA
Lpar1	QKI	Limd2	TIMM17B
Cbx1	ALKBH5	Pkd2l2	ADIG
Fyttd1	ITGB1BP1	Gm10094	POMGNT2
Qk	TCF15	Gm3230	ATP5PF
Alkbh5	PABIR1	Tmem246	MAST1

Itgb1bp1	RASSF3	Unc5c	MTX2
Tcf15	STYXL2	Dut	BRWD3
Fam122a	VPS72	Endog	MAN2A2
Rassf3	STIM2	Uqcrc2	GLT8D1
Dusp27	YLPM1	2510002D24Rik	FBXO31
Vps72	ARL2BP	Il7	AMIGO3
Stim2	HCLS1	Ppp2r5d	OGN
Ylpm1	SNRNP27	Fndc9	GPAM
Arl2bp	R3HCC1	Acta1	HTRA1
Hcls1	UBLCP1	Rpl18	SRR
Snrnp27	LPL	Anks3	PRNP
R3hcc1	KPNA4	Wdr70	TCTN1
Ublcp1	PABPN1	Hdac3	GLT8D2
Lpl	TRIM72	Rpl8	NAIF1
Kpna4	PREX2	Slfn8	PARP9
Pabpn1	MBD3	Gm10007	IL31RA
Trim72	CHMP3	Nrg4	MZB1
Prex2	MYO1E	Gper1	RPL37
Mbd3	MYO10	Gabpa	TMEM175
Chmp3	KIF16B	2210039B01Rik	ZSCAN2
Myo1e	PNISR	Timm17b	CCDC122
Myo10	YBX1	Adig	HEATR5B
Kif16b	TMSB4X	Pomgnt2	DENND4C
Pnisr	PDS5B	Atp5j	NFKBIB
Ybx1	KIN	Mast1	CHKB
Tmsb4x	TNMD	Mtx2	ADI1
Pds5b	PIK3C3	Brwd3	P2RY2
Kin	CYP27A1	Man2a2	ALOX12
Tnmd	COL4A1	Glt8d1	GPR39
Pik3c3	FBXO32	Fbxo31	ANGEL2
Cyp27a1	TBC1D5	Mir6357	PRSS57
Col4a1	TMEM245	Amigo3	EME1
Fbxo32	MARCKS	Ogn	RMI1
Tbc1d5	EFNB2	Gpam	RAD18

Tmem245	OBSCN	9530080O11Rik	RPS4X
Marcks	SPCS3	Htra1	RPS29
Efnb2	RMND5A	Srr	DCTN6
Obscn	BFAR	Prnp	TNK1
Spcs3	MUM1	Tctn1	EML6
Rmnd5a	TAF3	2310068J16Rik	NPDC1
Bfar	DMBT1	Glt8d2	TBC1D8
Mum1	LBH	Naif1	STARD7
Taf3	AKAP6	Parp9	STBD1
Dmbt1	COL23A1	1110057K04Rik	SAP18
Lbh	CROCC	Il31ra	PRSS53
Akap6	SOX13	Mzb1	MBNL1
Col23a1	BMS1	Igj	TNNC2
Crocc	HAX1	Rpl37	CD79A
Sox13	SEPT8	Tmem175	TEF
Bms1	AHCTF1	Zscan2	RPS13
Hax1	SRSF11	Ccdc122	CFB
Sept8	BDP1	Heatr5b	SLC5A2
Ahctf1	CCDC88C	Dennd4c	C1orf112
Srsf11	VASP	4430402I18Rik	ZFPM2
Bdp1	GPBP1L1	Nfkbib	ERMARD
Ccdc88c	WRNIP1	Chkb	RYBP
Vasp	PRKRIR	Adi1	RPL9
Gpbp1l1	CA2	Gm10638	VASN
Wrnip1	PHLDA1	1700018L02Rik	GP1BA
Prkrir	TMEM38B	Mir5128	NRARP
Car2	DHX8	P2ry2	ALG2
Phlda1	NUCB1	Alox12	FANCA
Tmem38b	CKAP5	Gpr39	ADAMTSL4
Dhx8	NPR3	Angel2	PLCXD1
Nucb1	C19orf43	Prss57	CNBD2
Ckap5	GSE1	A430090L17Rik	TSPAN4
Npr3	PEX14	Eme1	PLEKHM3
2310036O22Rik	ENG	Rmi1	RAC2

Gse1	EIF6	Rad18	NDUFA7
Pex14	PPP1R12A	Gm15612	NT5M
Eng	CPEB4	Rps4x	CASP2
Eif6	COL6A6	4933416M07Rik	RPL36A
Ppp1r12a	YAF2	Rps29	BROX
Cpeb4	CAPN2	Dctn6	KIF11
Col6a6	MLLT4	Tnk1	ZNF689
Yaf2	CREBZF	4933421O10Rik	CHMP1A
Capn2	YWHAH	Eml6	CD151
Mllt4	RND3	Npdc1	GAPDH
Crebzf	DUSP8	Tbc1d8	NUSAP1
Ywhah	PRR24	Stard7	ZNF593OS
Rnd3	FGD5	Stbd1	SMIM13
Dusp8	DNAL4	8030423F21Rik	EXOSC9
Prr24	PTPRG	Sap18	SMPD5
Fgd5	SSFA2	Prss53	NXT1
Dnal4	DKK3	Mbnl1	UXT
Ptprg	CCDC9	Tnnc2	RASSF4
Ssfa2	DHDH	Cd79a	RFXANK
Dkk3	FZD7	Tef	EIF2B2
Ccdc9	TUSC5	Rps13	ARL1
Dhdh	CHD3	Cfb	PBLD
Fzd7	ASB2	4930474M22Rik	PIGF
Tusc5	CDK19	Slc5a2	PIH1D1
Chd3	SMARCC2	BC055324	TMPRSS11A
Asb2	LOH12CR1	Zfpm2	UBXN6
Cdk19	PET100	Gm3435	SNRPA
Smarcc2	HDGF	Rybp	MAPK1
Loh12cr1	PPP3R1	Rpl9	NFYC
Pet100	PPP1R12B	Vasn	ITGBL1
Hdgf	UBE2F	Gp1ba	MRPS7
Ppp3r1	DACH1	Ctage5	ALG1
Ppp1r12b	PHRF1	Nrarp	ASB11
Ube2f	STAC3	Alg2	COX6A1

Dach1	BOLA3	Fanca	MRPL48
Phrf1	ERBB2IP	A230020J21Rik	COX6C
Stac3	SFSWAP	Gm13031	ZNHIT1
Bola3	ARAP3	Adamtsl4	CDC42SE1
Erbb2ip	KPNA3	Plcxd1	NIT1
Sfswap	NRBP1	Cnbd2	TTL
Arap3	SUCO	Tspan4	ALDH16A1
Kpna3	SURF6	Plekhm3	IQCD
Nrbp1	RAB8A	Rac2	IQCB1
Suco	ZEB1	Ndufa7	DNAJB7
Surf6	TJP2	Nt5m	MZF1
Rab8a	ITGB1BP2	Casp2	C1orf21
Zeb1	MYH8	1700073E17Rik	ZDHHC7
Tjp2	FTSJ2	Rpl36al	PRKCZ
Itgb1bp2	RAI2	Brox	LCTL
Myh8	VASH1	Kif11	DHX58
Ftsj2	SEPT2	Hfe2	PIBF1
Rai2	SVIP	Zfp689	CLHC1
Vash1	FABP4	Chmp1a	MET
Sept2	CDR2	E230016M11Rik	TBC1D21
Svip	PHACTR1	Cd151	DBP
Fabp4	SRRT	Gapdh	SAMD8
Cdr2	NCOR2	Nusap1	PHKG2
Phactr1	ZCRB1	BC021785	SFRP4
Srrt	LBX1	Lphn2	SUSD1
Ncor2	ZBTB7A	E130218I03Rik	LY6E
Zcrb1	RBM24	1700109K24Rik	CLDN20
Lbx1	KIAA0754	Smim13	PLEK2
Zbtb7a	PCBP4	Exosc9	OVOL3
Rbm24	XPOT	Smpd5	METTL17
D830031N03Rik	CANX	Gm2027	TSPYL4
Pcbp4	SYNM	Nxt1	NOXO1
Xpot	COL22A1	Uxt	TRNT1
Canx	FAM117A	Gm10684	MMS19

Synm	ECM2	Rassf4	NDUFA4L2
Col22a1	TMEM194A	Gnb2l1	ATP6V1C2
Fam117a	UBOX5	4933439C10Rik	DXO
Ecm2	TM2D1	Rfxank	NOP2
Tmem194	CYTH1	Hils1	RDH5
Ubox5	GPRASP1	Eif2b2	MEA1
Tm2d1	NES	8430408G22Rik	EXOG
Cyth1	STARD10	Arl1	PARP12
Gprasp1	DHX16	Pbld1	PRIMPOL
Nes	NUDC	Pigf	VSIG4
Stard10	PTTG1IP	Thap6	ZC3H3
Dhx16	C11orf58	Pih1d1	NUP62
Nudc	AFAP1L2	Fam219aos	PLLP
Pttg1ip	SNX2	Tmprss11a	RPL37A
1110004F10Rik	SH3BP5L	Ubxn6	SIGLEC1
Afap1l2	SEC63	Snrpa	TBP
Snx2	EPS15	Mapk1	FARSB
Sh3bp5l	PRPF38B	Nfyc	CD248
Sec63	COL16A1	Itgbl1	ZMYM6
Eps15	PLIN1	Mrps7	COX8BP
Prpf38b	IL11RA	Alg1	TEX12
Col16a1	PDZRN3	Asb11	DSTYK
Plin1	SIPA1	Cox6a1	CMSS1
Il11ra1	ARHGEF28	Mrpl48	PDE8A
Pdzrn3	PPP2CB	Cox6c	TUSC1
Sipa1	GID4	Znhit1	RPS2
Arhgef28	HUWE1	Cdc42se1	KLHDC9
Ppp2cb	CARD10	Nit1	COMP
Gid4	HLA-A	Ttl	TMEM55B
Huwe1	PSMD2	Aldh16a1	ALB
Card10	HAPSTR1	Iqcd	CNRIP1
H2-D1	MSI2	Iqcb1	COMMD5
Psmd2	LLPH	Dnajb7	NME3
1810013L24Rik	DST	Nup62-il4i1	GFPT2

Msi2	ZBTB43	Mzf1	CMKLR1
Llph	TNKS1BP1	1700025G04Rik	TMEM167A
Dst	MMRN2	Zdhhc7	RCE1
Zbtb43	CD93	Prkcz	PGK1
Tnks1bp1	MTHFD1	Lctl	RPL10
Mmrn2	BCL2L1	Dhx58	NRP1
Cd93	DDX24	Mir1932	SLC3A2
Mthfd1	GDI1	Pibf1	WBSCR22
Bcl2l1	CEP131	Clhc1	CAMK4
Ddx24	NUMA1	Met	MAVS
Gdi1	FAM131A	Tbc1d21	PEBP1
Cep131	DDIT4L	Dbp	MEG8
Numa1	NFATC1	Gm16796	SCN4A
Fam131a	ACIN1	Samd8	ING4
Ddit4l	BRAP	G630025P09Rik	SIK1
Nfatc1	PLEC	Mir1957b	STX18
Acin1	TMOD2	Phkg2	RRAGD
Brap	MRPS33	Sfrp4	PSMA5
Plec	RNF150	Susd1	METTL9
Tmod2	CACNB1	Ly6e	SLC1A7
Mrps33	AMD1	9330159M07Rik	GPX3
Rnf150	AMD1	Cldn20	SLC6A8
Cacnb1	LRRC8C	Fam103a1	C3orf38
Amd2	SAMD1	Mir6925	FAT1
Amd1	CASD1	Plek2	MED1
Lrrc8c	TAX1BP1	Ovol3	SLC7A10
Samd1	GPD1	D2Wsu81e	MTFR1L
Casd1	CLPTM1	Gm5607	DAK
Tax1bp1	MAP7D1	Mettl17	IFI16
Gpd1	MICU1	Tspyl4	CYP2E1
Clptm1	BLOC1S5	Noxo1	SRSF9
Map7d1	CLTB	Trnt1	NEURL1
Micu1	SCAF11	Mms19	RBM22
Bloc1s5	RNF40	Ndufa4l2	ZNF296

Cltb	ARHGAP31	Scarna2	PHYHD1
Scaf11	YTHDC1	Atp6v1c2	CRLS1
Rnf40	PTMA	C030034I22Rik	FUT2
Arhgap31	RBM39	Dxo	MMP2
Ythdc1	LRRFIP1	Nop2	RPL21
Ptma	TRIM8	Rdh5	C14orf119
Rbm39	SLU7	Mea1	A4GALT
Lrrfip1	NOC3L	Exog	WDR12
Trim8	LRRC2	Parp12	CD72
Slu7	CHD7	Primpol	ERLIN1
Noc3l	PSD3	Vsig4	MGAT4B
Lrrc2	MSRB2	Zc3h3	SURF1
Chd7	MYOZ3	Nup62	RPLP1
Psd3	MCM3AP	Pllp	DCPS
Msrb2	DNAJC17	Rpl37a	MED26
Myoz3	TIRAP	Siglec1	RFFL
Mcm3ap	DICER1	Tbp	LNX1
Dnajc17	MLLT6	Farsb	RPL30
Tirap	COL6A1	Inadl	PRPSAP2
Dicer1	CCDC174	Cd248	UCHL3
Mllt6	ANKRD11	Zmym6	TYMP
Col6a1	C9orf41	9630013A20Rik	RFX5
Ccdc174	EDF1	Cox8b	GTPBP3
Ankrd11	TEK	Tex12	THBD
2410127L17Rik	RTF1	Dstyk	NLN
Edf1	PRRC2B	Cmss1	HTRA2
Tek	PTPRB	Mterfd3	MPC1
Rtf1	BACH1	Pde8a	FAM169A
Prrc2b	REXO4	Tusc1	PLEKHH3
Ptprb	CTNNA1	Rps2	TRAPPC1
Bach1	MNAT1	Klhdc9	HSPB1
Rexo4	MAP2K7	Comp	C19orf54
Ctnna1	ANKRD28	Tmem55b	CCDC176
Mnat1	ACBD3	Alb	PKNOX2

Map2k7	DPP8	Cnrip1	GYG1
Ankrd28	MAT2B	Commd5	MYO19
Acbd3	PROB1	Nme3	XPO5
Dpp8	QRICH1	Gfpt2	ILK
Mat2b	PDE12	Cmklr1	SETD6
Prob1	SDCCAG8	Tmem167	PKD1L3
Qrich1	ZNF451	Rce1	CTF2P
Pde12	EWSR1	Pgk1	EDARADD
Sdccag8	MAZ	Rpl10	PRAP1
Zfp451	NUCB2	Nrp	SSR3
Ewsr1	HMHA1	4930599N23Rik	RPS3
Maz	HNRNPA2B1	9130024F11Rik	ATP5SL
Nucb2	DOCK4	Slc3a2	SPNS2
Hmha1	HDGFRP2	1700001G11Rik	NCBP2AS2
Hnrnpa2b1	ZBTB21	Wbscr22	ARRDC5
Dock4	OTUD7B	Camk4	LDHAL6A
Hdgfrp2	TRA2B	Mavs	NPTXR
Zbtb21	SEPT4	Pebp1	SMPD3
Otud7b	SIK3	Rian	WIF1
Tra2b	TRIM26	Scn4a	OMG
Sept4	ADPRHL1	Ing4	GOT1
Sik3	IGF2R	Sik1	EIF1
Trim26	NOC2L	Stx18	BCDIN3D
Adprhl1	NEXN	Rragd	COLGALT2
Igf2r	DNAJC11	B230312C02Rik	CHRD
Noc2l	MEG3	Psma5	IRF4
Nexn	HLA-A	1110008L16Rik	DNAJC27
Dnajc11	KRT13	Mettl9	TBC1D1
Meg3	SAFB2	Slc1a7	GSTP1
H2-Q10	DNAJC21	Gpx3	KLK8
Krt13	MYOZ1	Slc6a8	MUS81
Safb2	ITSN2	4930453N24Rik	SEMA3F
Dnajc21	NUDT9	Fat1	TIAM2
Myoz1	ZNF507	Med1	EPDR1

Itsn2	TNNT2	E030018B13Rik	HIST3H2A
Nudt9	MORF4L2	Zfp459	DDN
Zfp507	LIMCH1	Slc7a10	GAS5
Tnnt2	FAM20B	Mtfr1l	ZEB1-AS1
Morf4l2	MYLK	Dak	H2AFX
Limch1	G3BP1	Gm4262	ID2
Fam20b	SRSF5	Ifi205	IGHMBP2
Mylk	PACSIN3	Cyp2e1	KLHL17
G3bp1	RBM12	Srsf9	RAMP3
Srsf5	CCDC149	Neurl1a	SLC29A2
Pacsin3	AAGAB	Rbm22	TMEM117
Rbm12	CCDC32	Zfp296	ZBTB2
Ccdc149	NCALD	Phyhd1	PSTPIP1
Aagab	CEL	Crls1	MCM2
Ccdc32	ERP44	Fut2	MCM3
Ncald	EVPL	Mmp2	OSTC
Cel	PTCD2	Rpl21	P2RX7
Erp44	SCMH1	Gm13446	POLD1
Evpl	SIX2	1700123O20Rik	SLIT1
Ptcd2	TIMM10	A4galt	BBS5
Scmh1	KIF1B	Wdr12	BTBD8
Six2	CC2D2A	Cd72	HRH4
Timm10	UBE2H	Erlin1	PROM1
Kif1b	SDE2	Mgat4b	PRPS1L1
Cc2d2a	POSTN	Surf1	TLR4
Ube2h	SMU1	Rplp1	TSKU
Sde2	JPH1	Dcps	UQCR11
Postn	GPR116	Med26	ZNF605
Smu1	HMBOX1	2410076I21Rik	SHMT1
Jph1	MYH3	Rffl	ASB6
Gpr116	ERC1	Lnx1	CFP
Hmbox1	ELN	Rpl30	EGR3
Myh3	LSM6	Gm5088	FGF16
Erc1	RELL1	Prpsap2	GPRIN1

Eln	PYGL	Uchl4	MIR29B2
Lsm6	ANP32E	Tymp	PDLIM2
Rell1	CEP250	Rfx5	SLFN12
Pygl	NIPBL	Gm6307	SPG21
Anp32e	SEC11C	Gtpbp3	TRPC5
Cep250	ZC3H12C	Thbd	TYROBP
Nipbl	CTNNB1	A330032B11Rik	VTA1
Sec11c	MAF	Nln	ATG4D
Zc3h12c	PHF2	Htra2	ANKRD13B
Ctnnb1	HLA-A	Mpc1	CES1
Maf	CEP85	Fam169a	DCLRE1A
Phf2	DCAF12	Plekhh3	DNAL1
H2-K1	USP53	Trappc1	FAM102A
Cep85	SREK1	Hspb1	FOPNL
Dcaf12	CLEC2A	2310001K24Rik	GPD1L
Usp53	HTATSF1	BC024978	HNMT
Srek1	IGBP1	1700120C14Rik	JAGN1
Clec2d	CIB2	Ccdc176	LEPROTL1
Htatsf1	SORT1	Gm12191	MPC2
Igbp1	ZNF516	Pknox2	PLK3
Cib2	GOLIM4	Gyg	RBM41
Sort1	CHD1	Myo19	SMOC1
Zfp516	SHROOM4	Xpo5	SRGN
Golim4	PCNT	Ilk	TNNI1
Chd1	CSDE1	Setd6	KLF2
Shroom4	N4BP2	Pkd1l3	C12orf57
Pcnt	GNAS	Ctf2	RANBP6
Csde1	OCRL	Edaradd	MPZL2
N4bp2	FERMT2	Mir1258	BLOC1S3
Gnas	OPTN	Prap1	FAM166B
Ocrl	EHBP1L1	Ssr3	ADAM21
Fermt2	ITPKB	Rps3	WDR20
Optn	DCUN1D5	Atp5sl	FAM179A
Ehbp1l1	KAT2B	Spns2	UBE2F

Itpkb	SENP6	0610012G03Rik	HDDC3
Dcun1d5	ZNF764	Arrdc5	HSPA1A
Kat2b	CADM1	D630041G03Rik	MOGAT2
Senp6	ETS1	Ldhal6b	NDUFA3
Zfp764	CHD1L	Nptxr	NOD2
Cadm1	KIF21A	Smpd3	PSMB8
Ets1	ZBED4	Wif1	RAB4A
Chd1l	ZNF23	Omg	REEP6
Kif21a	SAMD4A	Got1	SLC12A8
Zbed4	C3orf18	Eif1	SNRNP25
Zfp612	SNRNP200	1700001K23Rik	ST7
Samd4	CDC5L	Bcdin3d	DNASE1L1
6430571L13Rik	CYTH2	Gm12070	MCRS1
Snrnp200	MOAP1	Mir8114	CAMTA2
Cdc5l	MTMR3	Colgalt2	CXCL10
Cyth2	SNRNP48	Chrd	OSGEPL1
4932702P03Rik	SELK	1700110K17Rik	ZNF750
Moap1	ARID5B	Irf4	SRA1
Gm20604	RAB3IL1	Dnajc27	ALG9
Mtmr3	SNRNP70	Tbc1d1	PADI2
Snrnp48	TNRC18	Gstp2	TUBGCP6
Selk	FLI1	Klk8	ARCN1
Arid5b	UBN2	Mus81	ACTG1
Rab3il1	RPL36	Sema3f	VPS28
Snrnp70	RORA	Tiam2	PER3
Tnrc18	DDX3Y	Epdr1	HOXD3
Fli1	KLC2	Hist3h2a	PAM16
Ubn2	TAF8	A630066F11Rik	SLC25A12
Rpl36	SMTNL1	B020004J07Rik	GREM2
Rora	KDR	Ddn	IFI27
Ddx3y	ZRANB2	Gas5	BRPF1
Klc2	TFAP2A	Gm10125	ZNF784
Taf8	ZBTB7B	Gm14092	MBD4
Smtnl1	DCAF6	H2afx	ITPKC

Kdr	ZNF652	Id2	FAM150B
Zranb2	GOPC	Ighmbp2	CISD1
Tfap2a	MRPL50	Klhl17	RPL12
Zbtb7b	DMTN	Ramp3	RHBDD3
Dcaf6	ENPEP	Slc29a2	SYNGR1
Zfp652	PLEKHG2	Tmem117	CCT3
Gopc	POLR1A	Zbtb2	S100A13
Mrpl50	RNF8	R74862	LDB1
Dmtn	LMO7	Pstpip1	CD38
Enpep	SVIL	4930467K11Rik	FDPS
Plekhg2	SALL2	Mcm2	ATP5F1,
Polr1a	GUCD1	Mcm3	RBM18
Rnf8	GOLGA2	Mir467c	RCHY1
Lmo7	SOGA1	Ostc	TPI1
Svil	MRPS15	P2rx7	UCKL1
Sall2	KIAA0247	Pold1	RPL13
Gucd1	TRIM41	Slit1	RPS10
Golga2	PDK1	2310001H17Rik	FANCI
Soga1	PARD3	2700070H01Rik	ERO1LB
Mrps15	DNMT3A	Asb17os	ARMCX3
4933426M11Rik	CD2BP2	Bbs5	AGFG2
Trim41	ZC3H4	Btbd8	PCNA
Pdk1	ZEB2	D030028A08Rik	TRMT10B
Pard3	C9orf78	Hrh4	GRN
Dnmt3a	SMC6	Prom1	MTRR
Cd2bp2	SEMA5A	Prps1l1	SKP2
Zc3h4	UPF3B	Tlr4	MCMBP
Zeb2	BASP1	Tsku	STAR
BC005624	AGTPBP1	Uqcr11	EXOC5
Smc6	SMARCE1	Zfp605	TOMM40L
Sema5a	PDCL3	Shmt1	SYN3
Upf3b	UTP3	9530051G07Rik	HLA-DRB1
Basp1	PRICKLE2	Asb6	STX5
Agtpbp1	RASD2	Cfp	IMMP1L

Smarce1	ZNF143	Egr3	CALCRL
Pdcl3	KCNK7	Fgf16	HSD17B7
Utp3	MDM1	Gprin1	COX5B
Prickle2	PRR14L	Lipo1	MAEA
Rasd2	TSG101	Mir29b-2	FABP3
Zfp143	ABHD16A	Pdlim2	SLC27A1
Kcnk7	HSD17B11	Slfn4	GNAL
Mdm1	TSPY26P	Spg21	AMPD1
Prr14l	SASH1	Trpc5	KXD1
Tsg101	FKBP4	Tyrobp	ANKRD61
Abhd16a	USP16	Vta1	CNPY4
Hsd17b11	ARMCX1	Atg4d	CTSA
Tspyl3	GIMAP1-GIMAP5	2610037D02Rik	SCAND1
AW112010	SLC7A8	4833418N02Rik	DOC2A
Sash1	DYX1C1	Ankrd13b	ST6GALNAC4
Fkbp4	GTF2F1	Ces1f	TMEM134
Usp16	OTUD4	Dclre1a	SDHB
Gm14207	NONO	Dnal1	THOC6
Armcx1	SLTM	Fam102a	RPL7
Gimap5	SAR1B	Fopnl	CMTR1
Slc7a8	PHEX	Gpd1l	HDGFRP3
Dyx1c1	WDR46	Hnmt	TRO
Gtf2f1	PHC1	Jagn1	STOML2
Otud4	UPF3A	Leprotl1	GPR146
Nono	ZBTB41	Mpc2	SPHK2
Sltm	TERF2IP	Plk3	GLCCI1
Sar1b	TAF12	Rbm41	RPS8
Phex	DHX38	Smoc1	RAD1
Wdr46	GGTA1	Srgn	GNG3
Phc1	USF2	Tnni1	CDC34
Upf3a	MVP	Klf2	CSRNP2
Zbtb41	AMOTL2	Grcc10	UBE2D1
Terf2ip	RAC1	4930404N11Rik	PLIN4
Taf12	ASPH	Ranbp6	SAMHD1

Dhx38	KIAA1671	AU021063	RPL11
Ggta1	NSA2	Mpzl2	MUC5B
Usf2	SLC9A3R2	Bloc1s3	TIGD5
Mvp	ANP32A	Fam166b	COCH
Amotl2	CEBPA	4930480K15Rik	CHID1
Rac1	EPT1	4933415F23Rik	KIF24
Asph	ANXA3	A530054K11Rik	GTF2I
2900026A02Rik	NELFCD	Adam21	CAMK2G
Nsa2	ZNF438	Dmr	MLXIP
Slc9a3r2	ANTXR2	Fam179a	ARPC1A
Anp32a	LUC7L2	Gm5434	TOM1L1
Cebpa	FER	Gm8439	CHAD
Ept1	PHACTR4	Hddc3	THUMPD3-AS1
Anxa3	SLC35B2	Hspa1b	INCA1
Nelfcd	FUT10	Mir7225	BMP2K
Zfp438	CDKN2AIP	Mogat2	FBXL4
Antxr2	TMEM170B	Ndufa3	LCMT2
Luc7l2	AGPAT2	Nod2	TEX10
Fert2	CALR	Psmb8	MTERFD2
Phactr4	AMOTL1	Rab4a	PLEKHB2
Slc35b2	ROCK2	Reep6	TM9SF1
Fut10	NSRP1	Slc12a8	NECAB1
Cdkn2aip	SGSM3	Snrnp25	PCIF1
Tmem170b	MYO18A	St7	ZNF385C
Agpat2	STK11	Dnase1l1	PDF
Calr	FOXN3	1700086O06Rik	STXBP2
Amotl1	STX12	Mcrs1	FAM63A
Rock2	PPM1G	Camta2	CHCHD1
Ccdc55	PPFIBP1	Cxcl10	DGAT2
Sgsm3	CCDC43	Osgepl1	RPL17
Myo18a	SRRM1	Zfp750	EIF2S3
Stk11	ERP27	Sra1	DUSP1
Foxn3	TCEAL8	Alg9	CA14
Stx12	ALDH1A2	Padi2	EEF1A1

Ppm1g	PSTPIP2	Tubgcp6	ATCAY
Ppfibp1	SNCAIP	Arcn1	TYMS
Ccdc43	AP3B1	Actg1	TEP1
Srrm1	POLR2D	Vps28	RPS18
Erp27	MDP1	Per3	PRDX1
Gm2518	CPLX2	Hoxd3	SLC25A5
Tceal8	AEBP2	Pam16	TARS2
Aldh1a2	CBX3	Slc25a12	YBEY
Pstpip2	MSL2	Grem2	PHC2
Sncaip	MPP6	Ifi27	RSPH3
Ap3b1	SPTB	Brpf1	SCO2
Polr2d	SCAF1	Zfp784	NUDT19
Mdp1	NF2	Mbd4	ZDHHC17
Cplx2	SPRYD3	Itpkc	HLF
Aebp2	SSR1	4930429B21Rik	NPR1
Cbx3	MSH2	Fam150b	LSM14B
Msl2	HOXC6	Cisd1	LIX1
Mpp6	STK11IP	Rpl12	PCDHGB7
Sptb	FILIP1	A930015D03Rik	MIR762
Scaf1	UBXN4	Rhbdd3	PCDHGA5
Nf2	RAB3GAP1	Syngr1	PPP6C
Spryd3	SLC38A10	Cct3	DAND5
Ssr1	DDX23	S100a13	RPS23
Msh2	SUPT16H	Ldb1	CENPJ
Hoxc6	DMPK	Cd38	KCNRG
Stk11ip	SPAG9	AY074887	MKRN2
Filip1	MTX3	Fdps	AK2
Ubxn4	TPM2	Atp5f1	FAM219B
Rab3gap1	NOLC1	Rbm18	NME6
Slc38a10	ARIH1	Rchy1	WDR48
Ddx23	MAFB	Tpi1	COMT
Supt16	DYNLL2	Uckl1	CAV2
Dmpk	PHLDB3	Rpl13	TTC7A
Spag9	OXSR1	Rps10	

Mtx3	YWHAE	Fanci	
Tpm2	CLSTN1	Ero1lb	
Nolc1	EEF1B2	Armcx3	
Arih1	ZNF326	Agfg2	
Mafb	PALM	Pcna	
Dynll2	AGO3	Trmt10b	
Phldb3	EIF1AX	Grn	
Oxsr1	COPB2	Mtrr	
Ywhae	AATF	Skp2	
Clstn1	RCSD1	Mcmbp	
Eef1b2	CDK11A	Star	
Zfp326	ZNF322	Exoc5	
Palm	EPB41L2	Tomm40l	
Ago3	ZC3H18	Syn3	
Eif1a	PPP1CC	H2-Eb1	
Copb2	NDRG2	Stx5a	
Aatf	TCOF1	Immp1l	
Rcsd1	KHSRP	Calcrl	
Cdk11b	SPECC1	Hsd17b7	
Zfp322a	EPHB4	Cox5b	
Epb4.1l2	ZFP91-CNTF	Maea	
Zc3h18	ISY1	Fabp3	
Ppp1cc	PRPF31	4930413G21Rik	
Ndrg2	CCDC85B	Slc27a1	
Tcof1	AKIRIN2	Gnal	
Khsrp	TNIP2	Ampd1	
Specc1	KLC1	Kxd1	
Ephb4	ATXN1L	Ankrd61	
Zfp91Cntf	UTRN	Cnpy4	
Isy1	ZMAT2	A230103J11Rik	
Prpf31	HOMER1	Ctsa	
Ccdc85b	PTK2	Scand1	
Akirin2	GTPBP4	Doc2a	
Tnip2	TMEM209	St6galnac4	

Klc1	CDC73	Tmem134	
Atxn1l	ATF1	Sdhb	
Utrn	CAMLG	Thoc6	
Zmat2	ACSL3	Rpl7	
Homer1	APP	Cmtr1	
Ptk2	RNF103	Hdgfrp3	
Gtpbp4	CCDC136	Tro	
Tmem209	NSUN2	Stoml2	
Cdc73	NAP1L1	Gpr146	
Atf1	ZFR	Sphk2	
Caml	MAP1LC3A	Glcci1	
Acsl3	C2orf68	Rps8	
App	PPP1R3E	Rad1	
Rnf103	CAPRIN1	Gng3	
Ccdc136	CCDC97	Cdc34	
Nsun2	RBBP6	Gm16712	
Nap1l1	TAF13	Csrnp2	
Zfr	PARK7	Gm19395	
Map1lc3a	SLX4	Ube2d1	
0610030E20Rik	MPHOSPH8	Plin4	
Ppp1r3e	NSMAF	Samhd1	
Caprin1	PSME1	Rpl11	
Ccdc97	TNNT3	Muc5b	
Rbbp6	CHMP6	Tigd5	
Taf13	SGK1	Coch	
Park7	AGTRAP	Chid1	
Slx4	KIAA1715	Kif24	
Mphosph8	SH3BP5	Gtf2i	
Nsmaf	STAG2	Camk2g	
Psme1	GAS8	Mlxip	
Tnnt3	KDM2A	2310061J03Rik	
Chmp6	VEGFA	Arpc1a	
Sgk1	UBR2	Tom1l1	
Agtrap	KLF6	4930526I15Rik	

Lnp	RHOJ	Chad	
Sh3bp5	POLR3GL	Gt(ROSA)26Sor	
Stag2	SPEN	Inca1	
Gas8	STOM	Bmp2k	
Kdm2a	EPC2	Fbxl4	
Vegfa	HNRNPD	Lcmt2	
Ubr2	ST3GAL5	Tex10	
Klf6	AKAP8	Mterfd2	
Rhoj	TMX4	Plekhb2	
Polr3gl	KIAA2013	Tm9sf1	
Spen	CDCA7	Necab1	
Stom	TRIM21	Pcif1	
Epc2	GNA11	Zfp385c	
Hnrnpd	TBKBP1	Pdf	
St3gal5	CCDC124	4930455D15Rik	
Akap8	COL6A5	Stxbp2	
Tmx4	IL1RL1	Fam63a	
2510039O18Rik	MITF	Chchd1	
Cdca7	GCOM1	Dgat2	
Trim21	NDUFAF5	9530091C08Rik	
Gna11	PERP	Man2c1os	
Tbkbp1	PPWD1	Rpl17	
Ccdc124	TOM1	Eif2s3x	
Col6a5	WDR41	G530011O06Rik	
Il1rl1	ZC3H13	F930015N05Rik	
Mitf	MBD2	Dusp1	
Myzap	HYPK	2900076A07Rik	
Ndufaf5	ABCE1	Car14	
Perp	KLHL20	Eef1a1	
Ppwd1	DUSP26	Atcay	
Tom1	AGRN	Tyms	
Wdr41	SLC15A3	Tep1	
Zc3h13	PIGK	Rps18	
Mbd2	UBA6	Prdx1	

Hypk	ZBED3	Slc25a5	
Abce1	PTPN13	Tars2	
Klhl20	LYVE1	Ybey	
Dusp26	ZNF117	Phc2	
Agrn	GRIK5	Rsph3a	
Slc15a3	EEF2K	Sco2	
Pigk	PCDH7	Nudt19	
Uba6	LAMA4	Gm16793	
Zbed3	RASAL2	Zdhhc17	
Ptpn13	CALML3	Hlf	
Lyve1	VAPB	Npr1	
Zfp866	DCUN1D1	Lsm14b	
Grik5	DSP	Lix1	
Eef2k	ZBTB12	Pcdhgb7	
Pcdh7	AGAP3	Mir762	
Lama4	CRCP	Pcdhga5	
Rasal2	TENM4	Ppp6c	
Calml3	DDX27	Dand5	
Vapb	SIPA1L2	Rps23	
Dcun1d1	CAV1	Cenpj	
Dsp	STX2	Kcnrg	
Zbtb12	EDC4	Mkrn2	
Agap3	PTPRM	Gm15417	
Crcp	ZNF706	Ak2	
Tenm4	ZBTB47	Fam219b	
Ddx27	MSS51	Nme6	
Sipa1l2	ATG101	Wdr48	
Cav1	NTHL1	Comt	
Stx2	THRAP3	Cav2	
Edc4	EXOC1	Ttc7	
Ptprm	LORICRIN		
Zfp706	CHAC2		
Zfp651	EXT1		
Mss51	ADAM9		

Atg101	PPIA		
Nthl1	NIPAL3		
Thrap3	AHDC1		
Exoc1	POLA1		
Lor	DLL1		
Chac2	ALPK3		
Ext1	CFL2		
Adam9	SNHG11		
Ppia	TOB2		
Nipal3	IGF1R		
Ahdc1	GPATCH11		
Pola1	PLEKHA1		
Dll1	ARHGAP17		
Alpk3	KALRN		
Cfl2	CFDP1		
AY512931	CUX1		
Snhg11	XDH		
Tob2	CITED2		
Igf1r	SKI		
Gpatch11	SMEK1		
Plekha1	PEA15		
5930403L14Rik	ADCK1		
Arhgap17	ARHGAP35		
Kalrn	IFIT2		
Cfdp1	INTS7		
Cux1	EIF3E		
Xdh	PPP2R2A		
Cited2	MORC3		
Ski	SFR1		
Smek1	VLDLR		
Pea15a	DRAP1		
Adck1	GOLGA4		
Arhgap35	PPIG		
Ifit2	BCLAF1		

Ints7	RBM34		
Eif3e	TTC9		
Ppp2r2a	FBN1		
Morc3	SMG1		
Sfr1	CLASP2		
Vldlr	VTI1B		
Drap1	KCTD12		
Golga4	ENSA		
Ppig	SPARCL1		
Bclaf1	SWAP70		
Rbm34	ARID4A		
Ttc9	PHLDB1		
4930521E06Rik	MCF2L		
Fbn1	GPS2		
Smg1	CREBRF		
Clasp2	VPS53		
Vti1b	SESN1		
Kctd12	DNMBP		
Ensa	YIPF4		
Sparcl1	YWHAG		
Swap70	ARHGAP21		
Arid4a	CRIP2		
Phldb1	EIF3C		
Mcf2l	KRCC1		
Gps2	RAB13		
Crebrf	FBXO11		
Vps53	RBMX2		
Sesn1	SLC25A26		
Dnmbp	RBM27		
Yipf4	IFNGR1		
Ywhag	EPHA4		
Arhgap21	SMARCD2		
Crip2	POLE3		
Eif3c	ARHGEF26		

Krcc1	RBM25		
Rab13	PRKAR2B		
Fbxo11	NUPR1		
Rbmx2	GLYR1		
Slc25a26	WBP5		
Rbm27	NDUFB7		
Ifngr1	ZC3H15		
Epha4	PPP1R9B		
Smarcd2	MAPK6		
Pole3	MMP15		
Arhgef26	RNF41		
Rbm25	MYO6		
Prkar2b	ADCY9		
Nupr1	TRIM54		
Glyr1	C1QTNF9		
Wbp5	AES		
Ndufb7	UBB		
Zc3h15	MANBAL		
Ppp1r9b	LRMP		
Mapk6	TPM1		
Mmp15	CD36		
Rnf41	FRMD6		
Myo6	MPEG1		
Adcy9	FOXJ2		
Trim54	WDFY4		
C1qtnf9	ING1		
Aes	BCR		
Ubb	MID1IP1		
Manbal	MYBPC2		
Lrmp	AHCYL1		
Tpm1	CBFA2T3		
Cd36	COL4A3BP		
Frmd6	SIRT1		
Mpeg1	CDV3		

Foxj2	SOS1		
Wdfy4	CDC42BPG		
1700095B10Rik	PIP5K1C		
Ing1	CACNA2D1		
Bcr	CHD6		
Mid1ip1	ANP32B		
Mybpc2	CDC42		
Ahcyl1	DDX46		
Cbfa2t3	RHNO1		
Col4a3bp	HSP90AA1		
Sirt1	OCIAD2		
Cdv3	GNG12		
Sos1	SLC35D2		
Cdc42bpg	FNTA		
Pip5k1c	ABCA8		
Cacna2d1	GIMAP1		
Chd6	ETS2		
Anp32b	SPARC		
Cdc42	CMTM4		
Ddx46	MXI1		
Rhno1	ING2		
Hsp90aa1	GPATCH1		
Ociad2	CTNNBL1		
Gng12	CDR2L		
Slc35d2	AXIN2		
Fnta	MICAL3		
Abca8a	MYH11		
Gimap1	CALM1		
Ets2	MEF2A		
Sparc	SMAD3		
Cmtm4	OCIAD1		
Mxi1	ANAPC7		
Ing2	RSBN1L		
Gpatch1	SETD1B		

Ctnnbl1	HIVEP2		
Cdr2l	COL11A1		
Axin2	GADL1		
Mical3	DYNC2LI1		
Myh11	SDPR		
Calm1	ORMDL3		
Mef2a	PRPF6		
Smad3	GKAP1		
Ociad1	HOXC10		
Anapc7	CHMP2B		
Rsbn1l	CCDC3		
Setd1b	BCL6B		
Hivep2	DYRK2		
Col11a1	LRRC45		
Gadl1	PIGT		
Dync2li1	PRSS48		
Sdpr	SLC22A15		
Ormdl3	TRAM1		
Prpf6	WBSCR27		
Gkap1	WDR45B		
Hoxc10	ARAF		
Chmp2b	CDH19		
Ccdc3	DPP6		
Bcl6b	KCNN3		
Dyrk2	MAP4K1		
Lrrc45	MRPL22		
Pigt	PDE6A		
Prss48	REXO1		
Slc22a15	TMEM186		
Tram1	XK		
Wbscr27	ZDHHC6		
Wdr45b	BANP		
Araf	CHD5		
Cdh19	KIAA2012		

Dpp6	MAMSTR		
Kcnn3	MKX		
Map4k1	PRDX5		
Mrpl22	PRKD1		
Pde6a	RPLP2		
Rexo1	SLC35G1		
Tmem186	WDR55		
Xk	WDR89		
Zdhhc6	DHX15		
2900056M20Rik	LOX		
Banp	RNF149		
Chd5	SCRIB		
Gm973	UBFD1		
Mamstr	SLC39A1		
Mkx	JUN		
Prdx5	NR1H2		
Prkd1	DES		
Rplp2-ps1	LUC7L3		
Slc35g1	SHANK3		
Wdr55	ARHGEF6		
Wdr89	APLP2		
Dhx15	RAB11FIP3		
Lox	SH3PXD2A		
Rnf149	LYAR		
Scrib	GEMIN7		
2610001J05Rik	SAMM50		
Ubfd1	EXOC6B		
Slc39a1	KIAA1549L		
Jun	SLC38A2		
Nr1h2	CCDC6		
Des	ROCK1		
Luc7l3	FAM199X		
Shank3	CASQ1		
Arhgef6	USP7		

Aplp2	TANC1		
Rab11fip3	CYB5R3		
Sh3pxd2a	KANK4		
Lyar	KRT4		
Gemin7	UNK		
Samm50	MAD2L1BP		
Exoc6b	BMPR2		
D430041D05Rik	ZFHX3		
Slc38a2	KIF13B		
Ccdc6	VPS36		
Rock1	TLN1		
Fam199x	KIAA1430		
Casq1	SART1		
Usp7	CSNK1A1		
Tanc1	MPHOSPH10		
Cyb5r3	MVB12A		
Kank4	MB21D2		
Krt4	SLC35A3		
Unk	SMAD7		
Mad2l1bp	ZBTB18		
Bmpr2	FAM114A1		
Zfhx3	CCDC15		
Kif13b	NAALAD2		
Vps36	FGF6		
Tln1	TEX261		
4933411K20Rik	PA2G4		
Sart1	SOX4		
Csnk1a1	HEATR2		
Mphosph10	GNAI1		
Mvb12a	KCNA2		
Mb21d2	HMGCR		
Slc35a3	ASB13		
Smad7	ATP6V1D		
Zbtb18	RNF144A		

Fam114a1	JSRP1		
Ccdc15	MYO18B		
Naalad2	RALBP1		
Fgf6	POGK		
Tex261	TAOK2		
Pa2g4	EIF3A		
Sox4	TXNDC16		
Heatr2	XPO1		
Gnai1	ZNF839		
Kcna2	ARHGEF1		
Hmgcr	UBE2N		
Asb13	FBXW11		
Atp6v1d	U2AF2		
Rnf144a	DDX50		
Jsrp1	PARP14		
Myo18b	RAD21		
Ralbp1	PAQR3		
Pogk	AP1S1		
Taok2	LMAN2L		
Eif3a	AGPAT5		
Txndc16	COL5A1		
Xpo1	KAT6B		
Zfp839	WNT5A		
Arhgef1	KLHL30		
Ube2n	TRAPPC10		
Fbxw11	UPF1		
U2af2	AAMDC		
Ddx50	ADAM10		
Parp14	C7orf60		
Rad21	NDE1		
Paqr3	PDE9A		
Ap1s1	KDM5D		
Lman2l	TFE3		
Agpat5	AKAP5		

Col5a1	AKAP9		
Kat6b	PLCE1		
Wnt5a	RCAN1		
Klhl30	RYR1		
Trappc10	CACYBP		
Upf1	UACA		
Aamdc	ZNF131		
Adam10	MXRA7		
B630005N14Rik	BAP1		
Mir466i	MAGI1		
2310020H05Rik	COX10		
Nde1	MTSS1L		
Pde9a	TARS1		
Kdm5d	KTN1		
Tfe3	JUP		
Akap5	ZNF830		
Akap9	SLK		
Plce1	COL5A2		
Rcan1	PVR		
Ryr1	TIFA		
Cacybp	POLK		
Uaca	DCTN1		
Zfp131	TM9SF3		
Mxra7	C18orf25		
Bap1	RANBP2		
Magi1	PHC3		
Cox10	ENPP2		
Mtss1l	HIC2		
Tars	MAB21L2		
Ktn1	TIMP2		
Jup	IRF1		
Zfp830	ZHX3		
Slk	SMARCA2		
Col5a2	SLC12A2		

Pvr	SSPN		
Tifa	MALAT1		
Polk	PALM2		
Dctn1	RBM45		
Tm9sf3	RPAP1		
8030462N17Rik	ANKRD50		
Ranbp2	MME		
Phc3	CASQ2		
Enpp2	PLEKHF2		
Hic2	FAM193B		
Mab21l2	NET1		
Timp2	DPF1		
Irf1	WDR74		
Zhx3	TARS3		
Smarca2	ARNT2		
Slc12a2	CHRM3		
Sspn	DENND3		
Malat1	ALKBH1		
Palm2	BDH1		
Rbm45	CBX4		
Rpap1	MYH4		
Ankrd50	TBX18		
Mme	RRP15		
Casq2	RNF152		
Plekhf2	SLMAP		
Fam193b	AFAP1		
Net1	CLN3		
Dpf1	MSANTD4		
Wdr74	TMEM57		
Tarsl2	PPP1CB		
Arnt2	ABL2		
Chrm3	YBX3		
Dennd3	MYH2		
Alkbh1	NID1		

Bdh1	AEBP1		
Cbx4	MAPKAPK2		
Myh4	EP300		
Tbx18	STRN3		
Rrp15	WIPF1		
Rnf152	UBAP2		
Slmap	ZNF292		
Afap1	MACROD1		
Cln3	VCP		
Msantd4	NRP1		
Tmem57	SLC39A7		
Ppp1cb	FRYL		
Abl2	CHPT1		
Ybx3	PLEKHG5		
Myh2	SPRY1		
Nid1	CENPB		
Aebp1	TNS1		
Mapkapk2	PXDN		
Ep300	AIRN		
Strn3	ATP10A		
Wipf1	LRP4		
Ubap2	SFMBT1		
Zfp292	MYD88		
Macrod1	UHRF2		
Vcp	LMBRD1		
Nrp1	PVRL2		
Slc39a7	HNRNPR		
Fryl	USP8		
Chpt1	STT3B		
Plekhg5	CHD4		
Spry1	THBS1		
Cenpb	RDH14		
Tns1	SATB1		
Pxdn	VWA1		

Airn	GPR82		
Atp10a	HIPK2		
Lrp4	CCDC12		
Sfmbt1	TSPAN15		
Gm15055	RPS25		
Myd88	AKAP1		
Uhrf2	RIOK3		
Lmbrd1	KHDRBS3		
Pvrl2	MYH9		
Hnrnpr	CAP2		
Usp8	L3MBTL3		
Stt3b	MIB1		
Chd4	DCAF13		
Thbs1	DCLK1		
Rdh14	HAT1		
Satb1	CPEB2		
Vwa1	SOX17		
Gpr82	RSU1		
Gm10336	IMMT		
Hipk2	FUS		
Ccdc12	COL3A1		
Tspan15	PTK7		
Rps25	MIPEP		
Akap1	ODF2L		
Riok3	TOP2B		
Khdrbs3	TACC2		
Myh9	HP1BP3		
Cap2	DNAJC2		
L3mbtl3	UPF2		
Mib1	PLCB4		
Dcaf13	PBXIP1		
Dclk1	CMYA5		
Hat1	CNST		
Cpeb2	RAI14		

Sox17	ZNF667		
Rsu1	KIAA1462		
Immt	C11orf83		
Fus	KCNJ15		
Col3a1	BRD7		
Ptk7	KPNA6		
Mipep	ZZEF1		
Odf2l	HMGN5		
Top2b	DBNL		
Tacc2	IGFBP5		
Hp1bp3	FGD4		
Dnajc2	ELK4		
Upf2	UBL3		
Plcb4	RPS17		
Pbxip1	EVL		
Cmya5	UIMC1		
Cnst	GXYLT1		
Rai14	BLOC1S1		
Zfp667	IFT46		
9430020K01Rik	MRPL18		
AI462493	PHF3		
Kcnj15	CWC27		
Brd7	MPZ		
Kpna6	NISCH		
Zzef1	ACTR8		
Hmgn5	ANKRD37		
Dbnl	TMEM39A		
Igfbp5	RBM5		
Fgd4	POLR2E		
Elk4	RAPGEF3		
Ubl3	C1RL		
Rps17	PDAP1		
Evl	CNOT7		
Uimc1	SMC1A		

Gxylt1	MRPS5		
Bloc1s1	PFDN1		
Ift46	PRR12		
Mrpl18	SYNPO2		
Phf3	ARHGEF25		
Cwc27	RRP1		
Mpz	TEAD1		
Nisch	BPTF		
Actr8	RSRC2		
Ankrd37	EBAG9		
Tmem39a	UBR1		
Rbm5	PLCL1		
Polr2e	ZFHX4		
Rapgef3	ICAM2		
C1rl	CCDC112		
2210010C04Rik	AP1S3		
Pdap1	STIM1		
Cnot7	ERICH1		
Smc1a	COQ2		
Mrps5	COL6A3		
Pfdn1	FZD6		
Prr12	GLE1		
Synpo2	LRRCC1		
Arhgef25	TXLNB		
Rrp1	GNB2		
Tead1	TMEM17		
Bptf	ATP11B		
Rsrc2	EIF4B		
Ebag9	GKN1		
Ubr1	TRAPPC3		
Plcl1	SEMA3C		
Zfhx4			
Icam2			
Ccdc112			

Ap1s3			
Stim1			
Erich1			
Coq2			
Col6a3			
Fzd6			
Gle1			
Lrrcc1			
Txlnb			
Gnb2			
Tmem17			
Atp11b			
Eif4b			
Gkn1			
Trappc3			
Sema3c			

Supplementary Table 4: Quantitative RT-PCR TaqMan Resources

Gene Name	ThermoFisher TaqMan Assay ID
Human SNX5	Hs00752139_s1
Human HPRT	Hs02800695_m1
Mouse ESR1	Mm00433149_m1
Mouse SNX5	Mm00445850_m1
Mouse MyoG	Mm00446194_m1
mouse MyoD	Mm00440387_m1
mouse alpha SMA	Mm00725412_s1
Mouse ROBO4	Mm00452963_m1
Mouse VEcadherin	Mm00486938_m1
Mouse IL6	Mm00446190_m1
Mouse PECAM-1	Mm01242576_m1
Mouse CD11	Mm00434455_m1
Mouse Flk1	Mm01222421_m1
Mouse TNFalpha	Mm00443258_m1
Mouse F4/80	Mm00802529_m1
Mouse HPRT	Mm00446968_m1

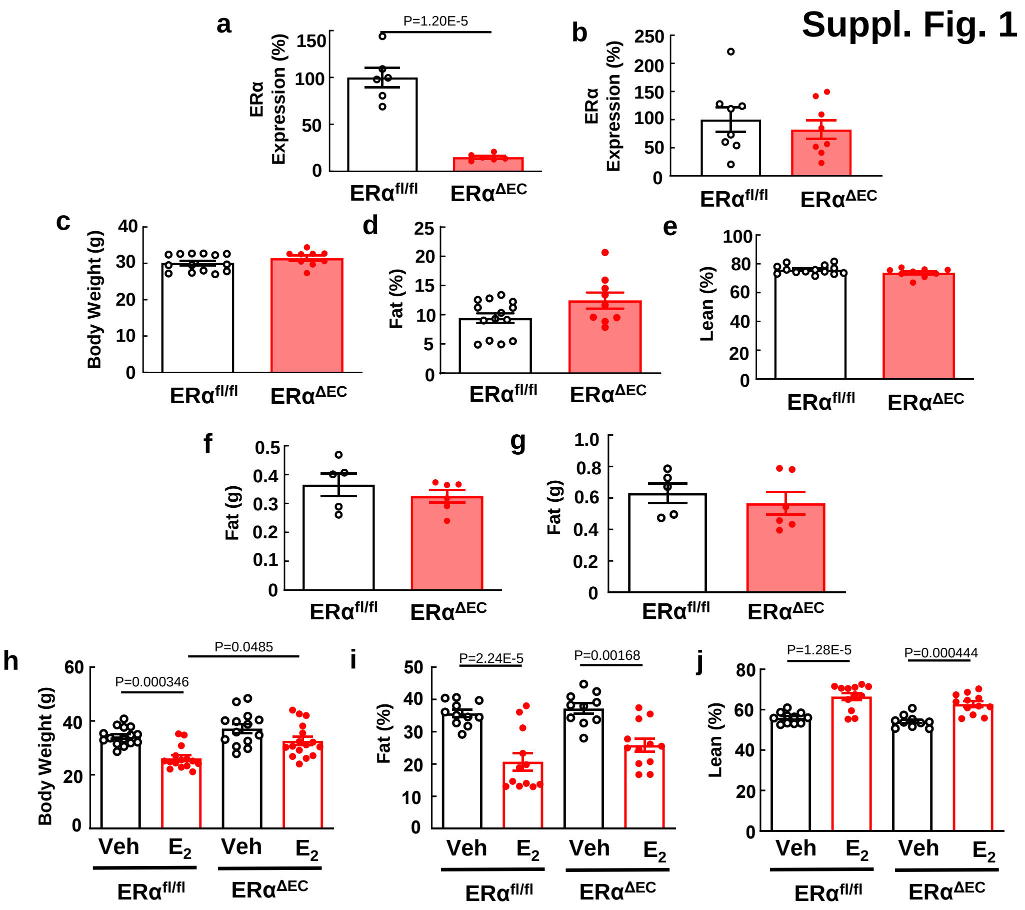

Supplementary Figure 1. Deletion of endothelial ERα does not influence adiposity in mice. **a, b.** ERα mRNA expression was evaluated in primary aortic endothelial cells (**a**) and myeloid lineage cells (**b**) from ERα$^{fl/fl}$ and ERα$^{\Delta EC}$ mice. N=6 mice per group and 8 mice per group in **a** and **b**. **c-e**. Body weight (**c**), fat mass (**d**, % of body weight) and lean body mass (**e**, % of body weight) were assessed in standard chow-fed male ERα$^{fl/fl}$ and ERα$^{\Delta EC}$ mice at 17 weeks of age. N=14 mice for ERα$^{fl/fl}$ and 9 mice for ERα$^{\Delta EC}$ groups. **f, g**. Subcutaneous (**f**, inguinal) and visceral (**g**, gonadal) WAT depot sizes were also compared, from 5 mice and 6 mice in ERα$^{fl/fl}$ and ERα$^{\Delta EC}$ groups, respectively. **h-j**. Body weight (**h**), fat mass (**i**) and lean body mass (**j**) were assessed in ovariectomized, high fat diet-fed ERα$^{fl/fl}$ and ERα$^{\Delta EC}$ female mice administered vehicle or E2 for 12 weeks. In **h**, n=15, 15, 14 and 16 mice per group, and in **i** and **j**, n=11, 12,

10 and 12 mice per group. Data are mean±SEM. Statistical analysis was by two-sided Student's t test (**a,b, d-g**), Mann Whitney (**c**), or Kruskal-Wallis with Dunn's post-hoc testing (**h-j**).

Suppl. Fig. 2

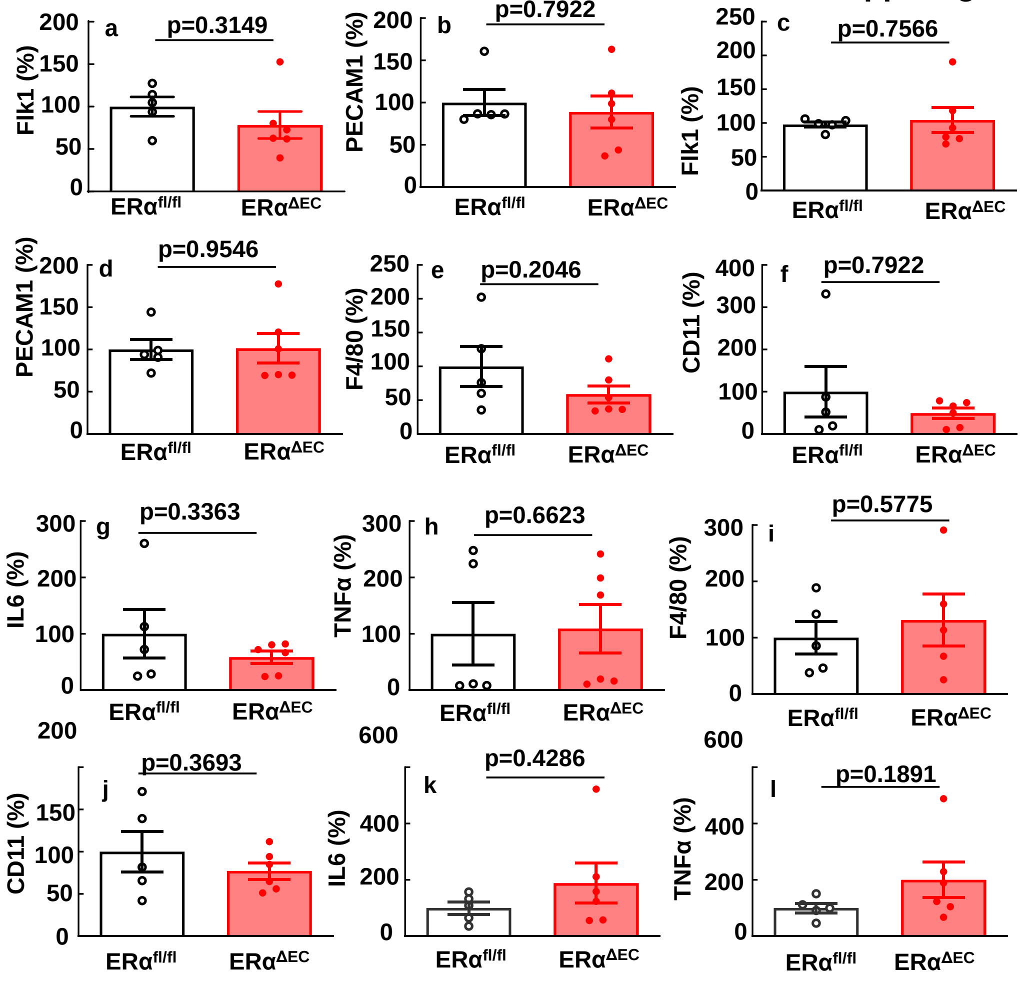

Supplementary Figure 2. Endothelial ERα does not impact adipose tissue vascularization or inflammation. In standard chow-fed male ERα$^{fl/fl}$ and ERα$^{\Delta EC}$ mice at 17 weeks of age, subcutaneous (**a**,**b**, inguinal) and visceral (**c**,**d**, gonadal) fat pads were harvested, and Flk1 (**a**,**c**) and PECAM-1 (**b**,**d**) transcript abundance was measured by Q-PCR. **e-l**. In the same subcutaneous (**e-h**) and visceral fat pads (**i-l**) transcript abundance was evaluated for F4/80 (**e**,**i**), CD11 (**f**,**j**), IL-6 (**g**,**k**) and TNFα (**h**,**l**). Data are mean±SEM, n=5 mice and 6 mice in ERα$^{fl/fl}$ and ERα$^{\Delta EC}$ groups, respectively, in all panels except **i**, in which n=5 mice per group. Statistical analysis was by two-sided Student's t test (**a**,**c**,**d**,**e**,**g**,**i**,**j**,**l**) or Mann Whitney (**b**,**f**,**h**,**k**).

Suppl. Fig. 3

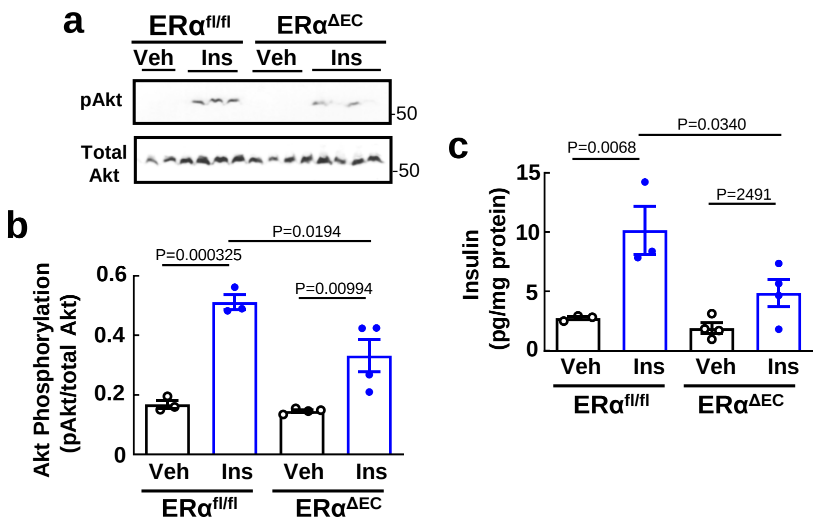

Supplementary Figure 3. Endothelial ERα promotes insulin action in skeletal muscle by enhancing insulin delivery. **a-c.**. In a cohort of mice independent of those reported in Figure 1, standard chow-fed males were intravenously injected with vehicle (Veh, saline) or insulin (ins, 1 unit/kg body wt), 5 min later skeletal muscle was isolated, and lysates were prepared for immunoblotting to detect phosphorylated Akt at Ser473 (pAkt) and total Akt. In **a**, example findings for 2 to 3 samples per group are shown, and quantification for n=3, 3, 4 and 4 mice is in **b**. **c**. In muscle samples from mice studied as in **a,b**, insulin content was quantified by ELISA (n=3, 3, 4 and 4 mice). Data are mean±SEM, and p values for one-way ANOVA with Tukey's post-hoc testing are shown.

Suppl. Fig. 4

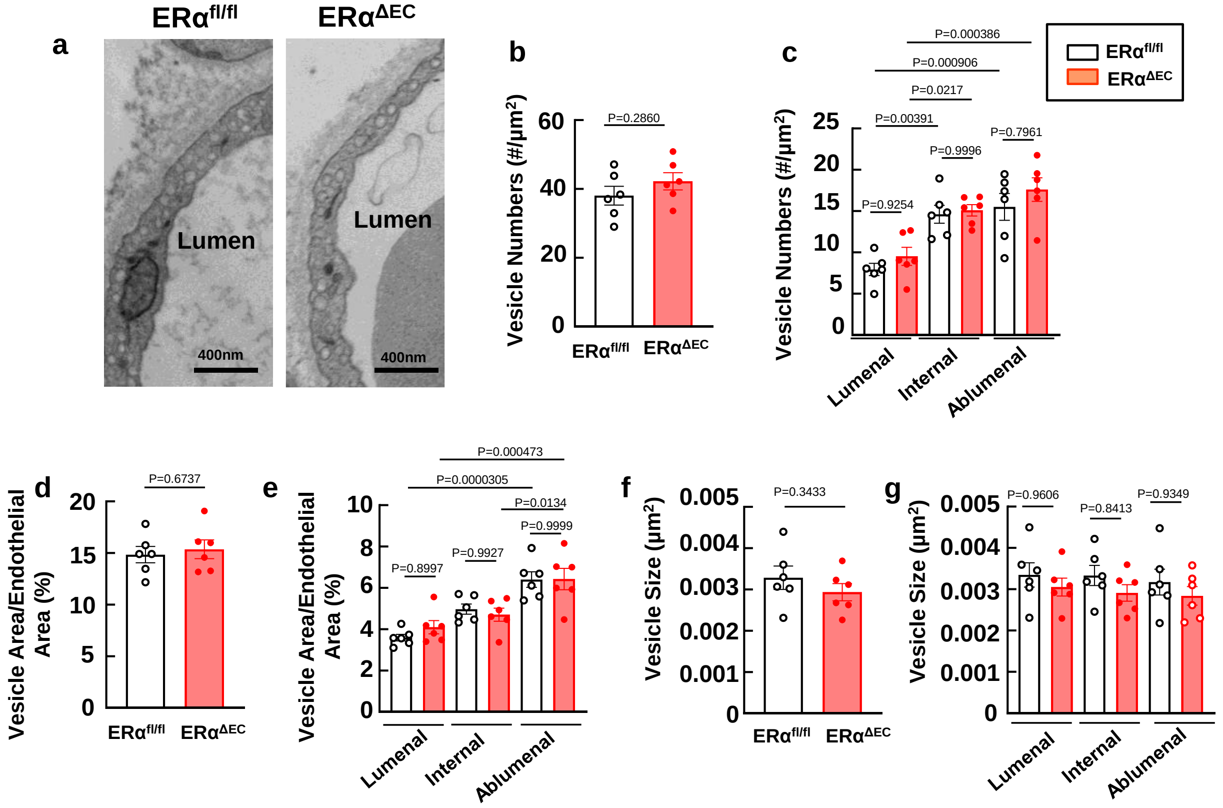

Supplementary Figure 4. Electron microscopy of skeletal muscle capillary endothelium. **a**. Images of capillary endothelium in gastrocnemius from male ERα$^{fl/fl}$ and ERα$^{\Delta EC}$ mice at 17 weeks of age following standard chow feeding since weaning. **b,c.** Vesicle number per unit endothelial area for all vesicles (**b**) and separately for luminal, abluminal and intracellular vesicles (**c**). **d,e.** Vesicle area per unit endothelial area for all vesicles (**d**) and separately for luminal, abluminal and intracellular vesicles (**e**). **f,g.** Individual vesicle size for all vesicles (**f**) and separately for luminal, abluminal and intracellular vesicles (**g**). N=6 mice/group, data are mean±SEM. Statistical analysis was by two-sided Student's t test (**b,d,f**) or one-way ANOVA with Tukey's post-hoc testing (**c,e,g**).

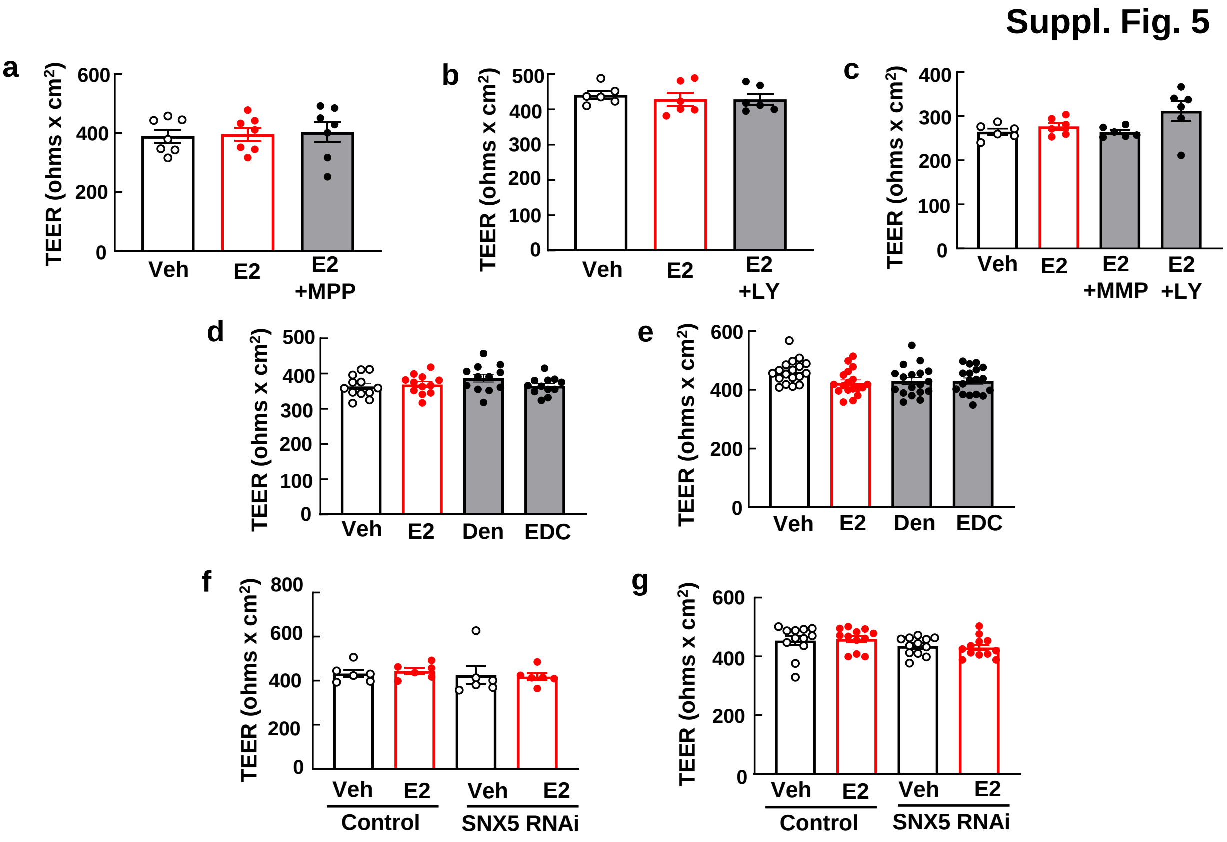

Supplementary Figure 5. In the interrogations of insulin transcytosis, measurements of transendothelial electrical resistance (TEER) on the day of study revealed intact monolayer barriers with similar degrees of integrity in all study groups within an experiment. **a-e**. TEER values are provided for experiments in HAEC (**a-c**) or HSMEC (**d,e**) that did not undergo genetic manipulation. In **a-c**, n=7, 6 or 6 wells of cells per treatment, respectively. In **d,** n=12 wells of cells per treatment, and in **e**, n=13 wells of cells per treatment. **f,g**. HAEC (**f**) and HSMEC (**g**) were transfected with control RNAi or RNAi targeting SNX5, and TEER measurements were done in the context of quantification of insulin transcytosis. N=6 wells per treatment, and n=12 wells of cells per treatment in **f** and **g**, respectively. Data are mean±SEM.

Suppl. Fig. 6

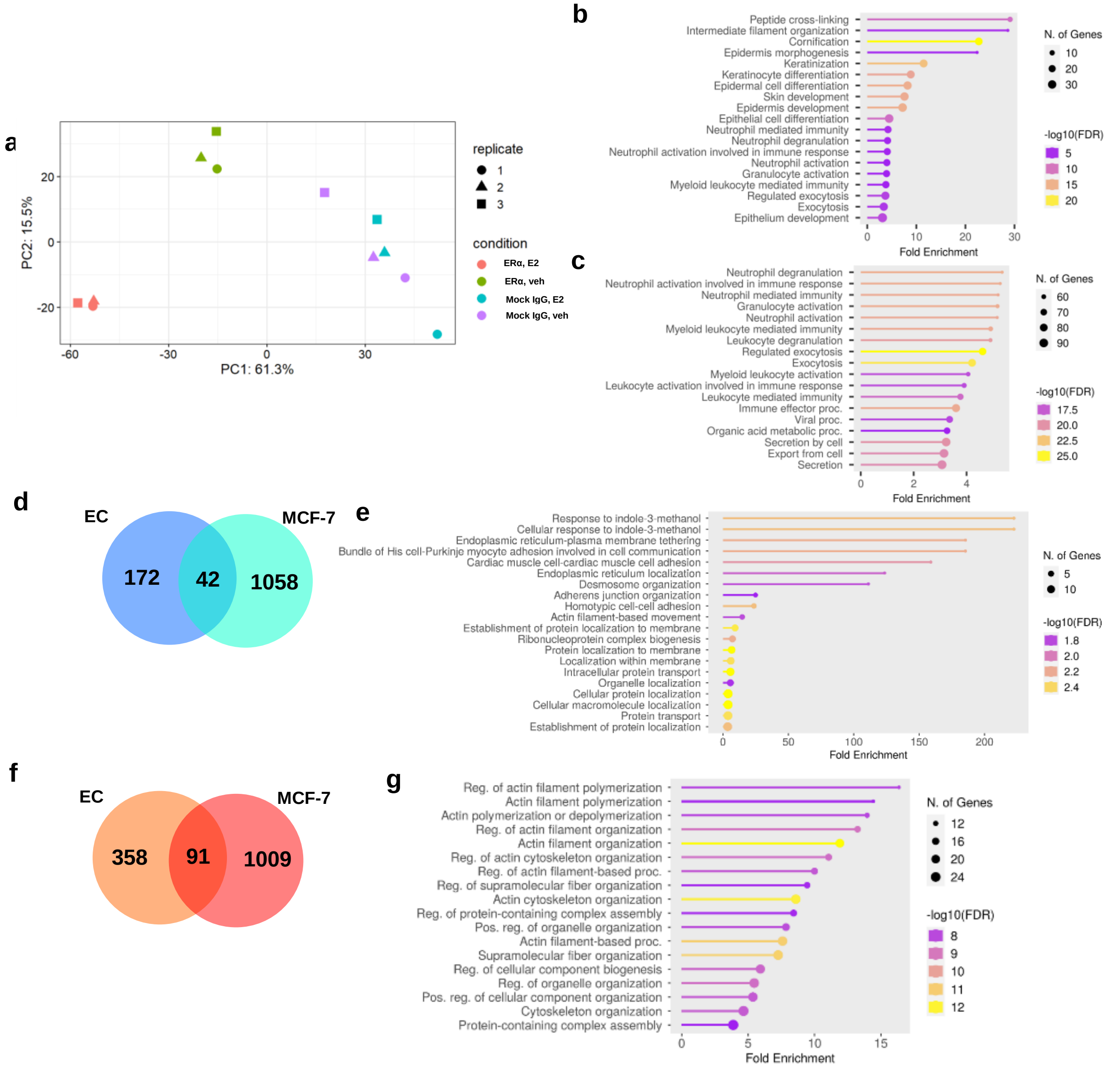

Supplementary Figure 6. The ERα interactome in endothelial cells includes both proteins disassociated from ERα and recruited to the receptor in response to E2. **a**. A PCA plot for the top 500 variable proteins detected in 3 biological replicates for 4 conditions reveals that discrete sets of proteins were identified to interact with ERα compared to mock samples, and that differing interactomes were detected with vehicle versus E2 treatment. **b,c**. Pathway analyses for proteins

disassociated from (**b**) or recruited to ERα (**c**) in response to E2. **d-g.** Venn diagrams and pathway analyses for the intersect of proteins dissociated from ERα in endothelial cells and components of the ERα interactome in MCF-7 cells (**d,e**), or for the intersect of proteins recruited to ERα in endothelial cells and components of the ERα interactome in MCF-7 cells (**f,g**). In **b,c,e,g** gene ontology gene sets are listed and fold enrichment ratios are shown for the top 20 pathways based on the false discovery rate ≤0.05.

Suppl. Fig. 7

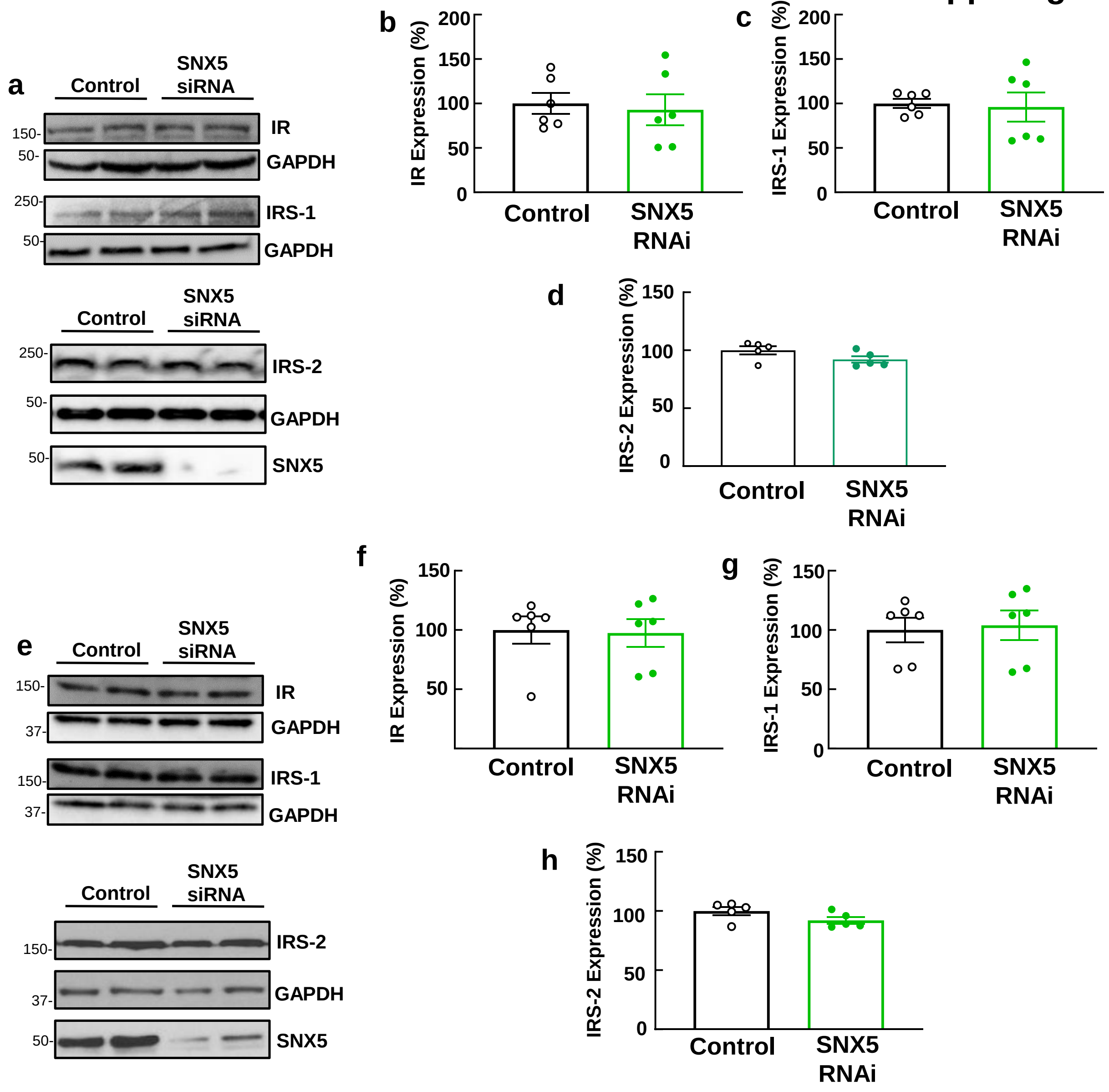

Supplementary Figure 7. Following SNX5 knockdown, IR, IRS-1 and IRS-2 expression are preserved in HAEC (**a-d**) and HSMEC (**e-h**). Representative immunoblots are shown in **a** and **e**, and summary data are provided for the quantification of IR expression (**b,f**), IRS-1 expression (**c,g**), and IRS-2 expression (**d,h**). Data are mean±SEM, and n=6 (**b,c,f,g**) or n=5 (**d,h**) wells of cells per study group.

Suppl. Fig. 3

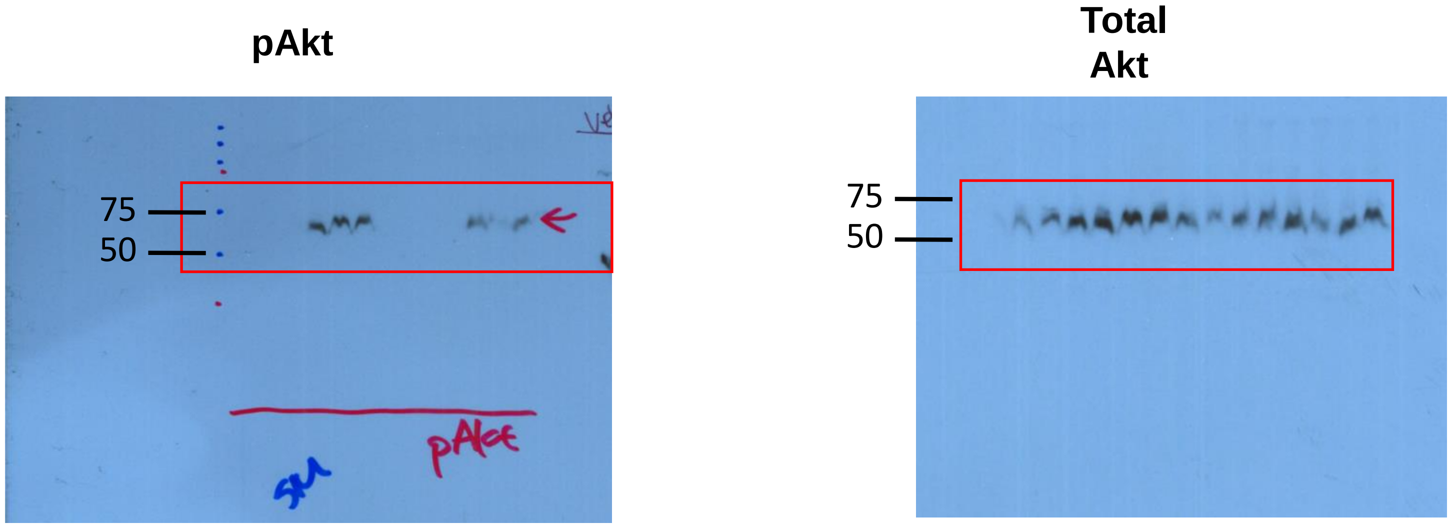

Suppl. Fig. 7a

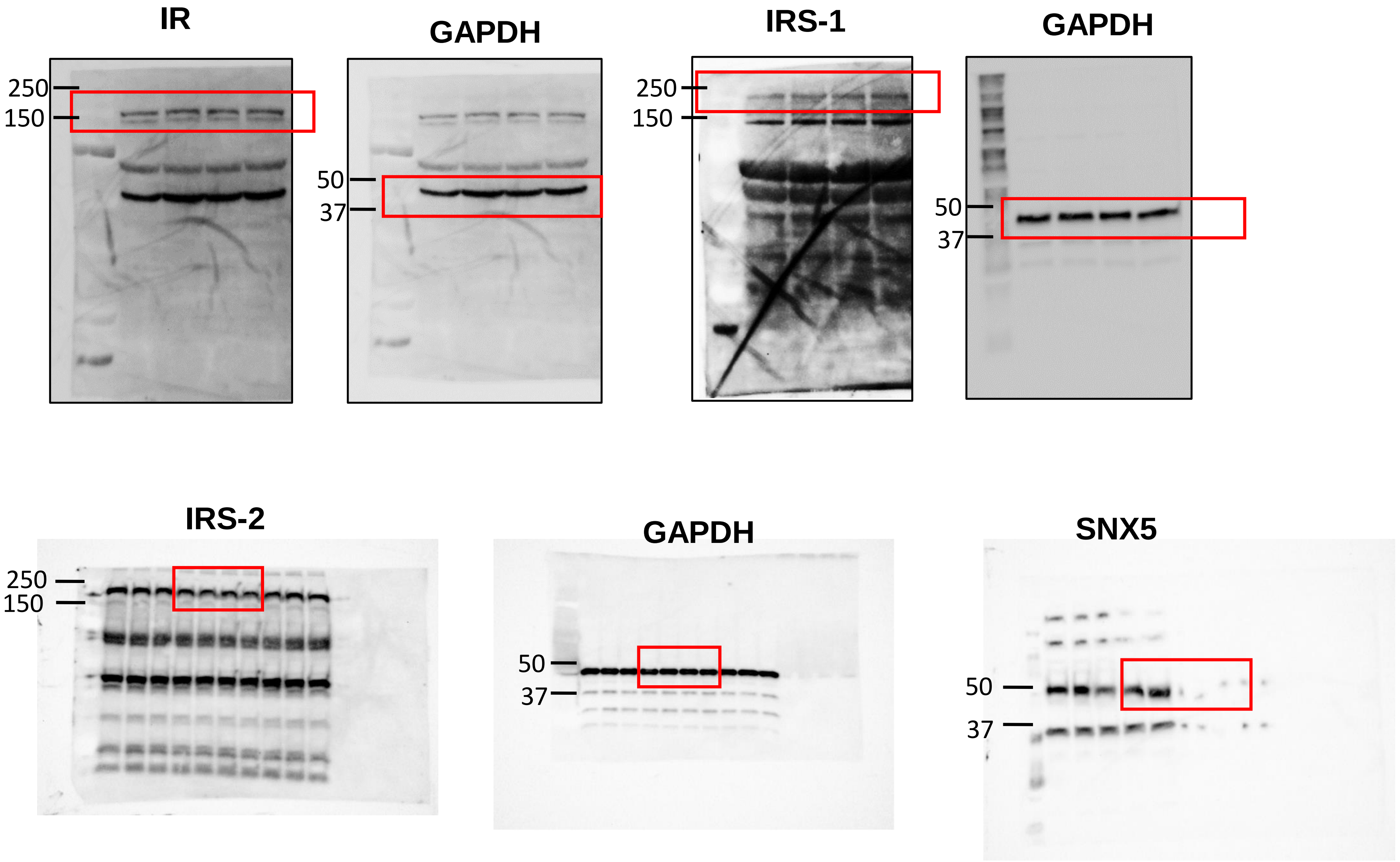
IR
250
150
GAPDH
50
37
IRS-1
250
150
GAPDH
50
37
IRS-2
250
150
GAPDH
50
37
SNX5
50
37

Suppl. Fig. 7e

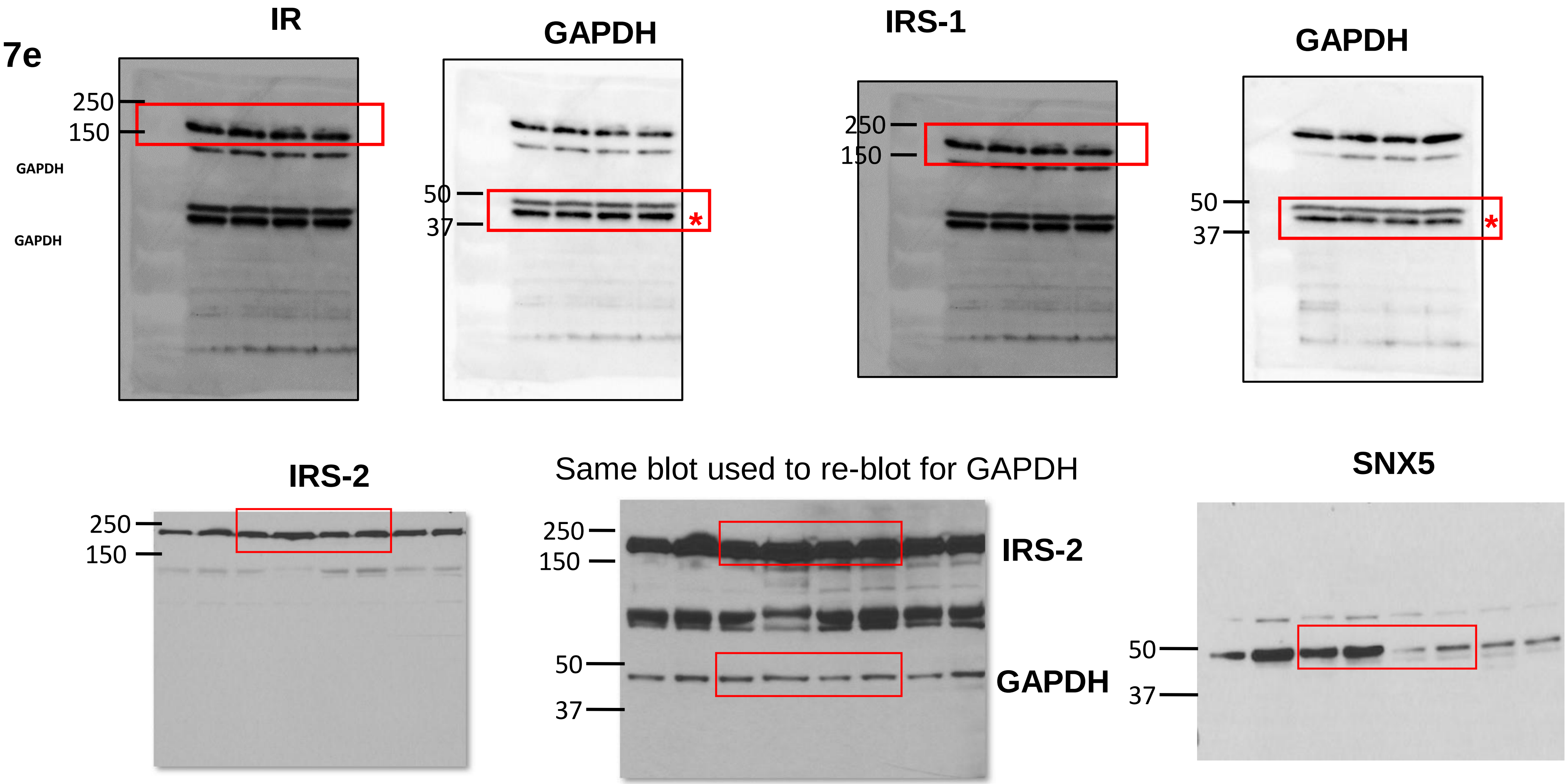

IR
GAPDH
IRS-1
GAPDH
250
150
GAPDH
GAPDH
50
37
*
250
150
50
37
*
IRS-2
Same blot used to re-blot for GAPDH
SNX5
250
150
250
150
IRS-2
50
37
GAPDH
50
37
