# Supplementary material for: Endothelial ERα promotes glucose tolerance by enhancing endothelial insulin transport to skeletal muscle
Source: Nat Commun. 2023 Aug 17;14:4989. doi: 10.1038/s41467-023-40562-w (PMC10435471; doi:10.1038/s41467-023-40562-w)
Supplement: Supplementary file 2 — Description of Additional Supplementary Files [file 41467_2023_40562_MOESM2_ESM.pdf]

Description of Additional Supplementary Materials

Supplementary Movie 1: Contrast-enhanced ultrasound imaging of a lipid shelled microbubble contrast agent in the skeletal muscle microvasculature of a control $ER\alpha^{fl/fl}$ male mouse at baseline.

Supplementary Movie 2: Contrast-enhanced ultrasound imaging of a lipid shelled microbubble contrast agent in the skeletal muscle microvasculature of a control $ER\alpha^{fl/fl}$ male mouse, showing increases in capillary recruitment and blood flow in response to a hyperinsulinemic-euglycemic clamp.
