# Supplementary material for: Endothelial ERα promotes glucose tolerance by enhancing endothelial insulin transport to skeletal muscle
Source: Nat Commun. 2023 Aug 17;14:4989. doi: 10.1038/s41467-023-40562-w (PMC10435471; doi:10.1038/s41467-023-40562-w)
Supplement: Supplementary file 5 — Reporting Summary [file 41467_2023_40562_MOESM5_ESM.pdf]

nature portfolio

Corresponding author(s): Philip W. Shaul and Chieko Mineo

Last updated by author(s): June 30, 2023

Reporting Summary

Nature Portfolio wishes to improve the reproducibility of the work that we publish. This form provides structure for consistency and transparency in reporting. For further information on Nature Portfolio policies, see our Editorial Policies and the Editorial Policy Checklist.

Statistics

For all statistical analyses, confirm that the following items are present in the figure legend, table legend, main text, or Methods section.

n/a	Confirmed	
☐	☒	The exact sample size (*n*) for each experimental group/condition, given as a discrete number and unit of measurement
☒	☐	A statement on whether measurements were taken from distinct samples or whether the same sample was measured repeatedly
☐	☒	The statistical test(s) used AND whether they are one- or two-sided<br>*Only common tests should be described solely by name; describe more complex techniques in the Methods section.*
☒	☐	A description of all covariates tested
☐	☒	A description of any assumptions or corrections, such as tests of normality and adjustment for multiple comparisons
☐	☒	A full description of the statistical parameters including central tendency (e.g. means) or other basic estimates (e.g. regression coefficient) AND variation (e.g. standard deviation) or associated estimates of uncertainty (e.g. confidence intervals)
☐	☒	For null hypothesis testing, the test statistic (e.g. *F*, *t*, *r*) with confidence intervals, effect sizes, degrees of freedom and *P* value noted<br>*Give P values as exact values whenever suitable.*
☒	☐	For Bayesian analysis, information on the choice of priors and Markov chain Monte Carlo settings
☒	☐	For hierarchical and complex designs, identification of the appropriate level for tests and full reporting of outcomes
☒	☐	Estimates of effect sizes (e.g. Cohen's *d*, Pearson's *r*), indicating how they were calculated

Our web collection on statistics for biologists contains articles on many of the points above.

Software and code

Policy information about availability of computer code

Data collection

Contrast-enhanced ultrasound data were recorded and processed using MATLAB software (MathWorks, version 2020b).

Data analysis

Raw mass spectrometry (MS) data files were analyzed using Proteome Discoverer v2.4 SPl (Thermo), with peptide identification performed using Sequest HT searching against the human reviewed protein database from UniProt (downloaded April 8, 2022, 20361 entries). Fragment and precursor tolerances of 10 ppm and 0.6 Da were specified, and three missed cleavages were allowed. Carbamidomethylation of Cys was set as a fixed modification, with oxidation of Met set as a variable modification. The false-discovery rate (FDR) cutoff was 1% for all peptides.

The statistical analysis of the MS data was performed using the Differential Enrichment analysis of Proteomics data (DEP)52 analysis workflow package (version 1.12.0), R version 4.0.2. Proteins found in 2 out of 3 replicates per condition were filtered for further downstream analysis. Variance Stabilizing Normalization was performed to normalize the intensities of the filtered proteins followed by "Min Prob" based missing value imputation. Differential expression testing of conditions was performed using limma empirical Bayes statistics by constructing linear models for proteins. FDR values were higher than expected, primarily due to variability between samples. Therefore, for significance (P < 0.05) in proteomics results we employed non-adjusted P values. To further identify the proteins of interest, we only filtered proteins significantly changed with anti-ER + E2 treatment compared to anti-ER + vehicle treatment but not significantly changed in the comparison between Mock IgG + E2 treatment vs Mock IgG + vehicle treatment.

In the RNAseq performed in TRAPseq, image intensities were processed using NextSeq 500 Control Software (Illumina) with default settings. Raw data were de-multiplexed and converted to fastq files using bcl2fastq (v2.17). The fastq files were checked for quality using fastqc (v0.11.2) (http://www.bioinformatics.babraham.ac.uk/projects/fastqc) and fastq_screen (v0.4.4) (http://www.bioinformatics.babraham.ac.uk/projects/fastq_screen). Sequencing reads were mapped to mml0 reference genome (from igenomes) using STAR, counted using featureCounts and normalized using trimmed mean of M values (TMM) methods. Differential expression analysis was performed using edgeR

(version 3.12).

Functional pathway analysis was performed using the ShinyGo application at ShinyGO 0.77 (sdstate.edu) with biological process terms from Biological Processes of Gene Ontology. For the overlap with proteomics data with TRAPseq data, we first utilized the databases of Homologene (https://www.ncbi.nlm.nih .gov/homologene) and MGI homology (http://www.informatics.jax.org/homology.shtml) to convert the TRAPSeq mouse data to human homologs. Venn diagrams were generated for the overlapping genes between LC-MS/MS (FDR<0.01) and human homologs of the TRAPseq differentially expressed genes (p<0.05) and log2 (counts per million) >0) (http://bioinformatics.psb.ugent.be/webtools/Venn/).

For manuscripts utilizing custom algorithms or software that are central to the research but not yet described in published literature, software must be made available to editors and reviewers. We strongly encourage code deposition in a community repository (e.g. GitHub). See the Nature Portfolio guidelines for submitting code & software for further information.

Data

Policy information about availability of data

All manuscripts must include a data availability statement. This statement should provide the following information, where applicable:

- Accession codes, unique identifiers, or web links for publicly available datasets
- A description of any restrictions on data availability
- For clinical datasets or third party data, please ensure that the statement adheres to our policy

Data Availability Statement: The raw LC/MS-MS data in this study has been uploaded to the MassIVE data repository with accession number MSV000091095 [https://massive.ucsd.edu/ProteoSAFe/dataset.jsp?task=2e6879ce383a4eb68b7b2e7ca57bb1b8]. The raw TRAPseq data in this study were deposited in GEO under record GSE179737 [https://www.ncbi.nlm.nih.gov/geo/query/acc.cgi?acc=GSE179737]. There are no restrictions on data availability. All differential proteomics data and integrated LC/ MS-MS and TRAPSeq analysis results are included in the Tables provided. Source data are provided with this paper.

Research involving human participants, their data, or biological material

Policy information about studies with human participants or human data. See also policy information about sex, gender (identity/presentation), and sexual orientation and race, ethnicity and racism.

Reporting on sex and gender	No studies were performed in humans or samples from humans.
Reporting on race, ethnicity, or other socially relevant groupings	No studies were performed in humans or samples from humans.
Population characteristics	No studies were performed in humans or samples from humans.
Recruitment	No studies were performed in humans or samples from humans.
Ethics oversight	No studies were performed in humans or samples from humans.

Note that full information on the approval of the study protocol must also be provided in the manuscript.

Field-specific reporting

Please select the one below that is the best fit for your research. If you are not sure, read the appropriate sections before making your selection.

☒ Life sciences ☐ Behavioural & social sciences ☐ Ecological, evolutionary & environmental sciences

For a reference copy of the document with all sections, see nature.com/documents/nr-reporting-summary-flat.pdf

Life sciences study design

All studies must disclose on these points even when the disclosure is negative.

Sample size	Regarding the in vivo studies of glucose homeostasis, in glucose tolerance tests (GTT) assessing mechanisms related to estrogen receptor biology, we previously observed differences in serum glucose at 45 min of 100 mg/dL and typical standard deviations of 66. Using these ctions and a Type I error of 5% and a Type II error of 10%, it was expected that 9 animals will be required for each group for GTT and related studies. No power calculations were performed for the other types of experiments pursued. Sample sizes were chosen based on standard practice for that type of experiment. For studies performed in mice, the number of independent mice employed is provided in the figure legend. In experiments in cell culture power analysis was not performed, and sample sizes were chosen based on experience with previous studies which indicated that n=6 per group is most often most sufficient to discern differences in endpoints between study groups (PMID: 29202472).
Data exclusions	No data were excluded from analysis.
Replication	Findings in in vivo mouse studies were replicated in 2-3 separate cohorts. Findings from cell culture studies were replicated at least 2 independent experiments.

Randomization	Within all genotype groups, age- and sex-matched animals were randomly assigned to the experimental groups.
Blinding	The investigators were not blinded to experimental group assignment in the mouse experiments because the group differences were genetically-based and not within the control of the investigator.

Reporting for specific materials, systems and methods

We require information from authors about some types of materials, experimental systems and methods used in many studies. Here, indicate whether each material, system or method listed is relevant to your study. If you are not sure if a list item applies to your research, read the appropriate section before selecting a response.

Materials & experimental systems

n/a	Involved in the study
☐	☒ Antibodies
☐	☒ Eukaryotic cell lines
☒	☐ Palaeontology and archaeology
☐	☒ Animals and other organisms
☒	☐ Clinical data
☒	☐ Dual use research of concern
☒	☐ Plants

Methods

n/a	Involved in the study
☒	☐ ChIP-seq
☒	☐ Flow cytometry
☒	☐ MRI-based neuroimaging

Antibodies

Antibodies used	Anti-Phospho-Akt (Ser473) Antibody (Cell Signaling, Cat #9271), anti-Akt (Cell Signaling, Cat #9272), rat monoclonal anti-mouse CD31 (Thermo Fisher Scientific, Cat#RM5200; RRID: AB_10376006), anti-mouse Mac-1 (BD Biosciences, Cat #557395), anti-ERalpha (Santa Cruz Biotech, Cat #sc-8002), anti-SNX5 (Abeam, Cat #ab5983), anti-GAPDH (Santa Cruz Biotech, Cat #sc-47724), anti-Calnexin (Santa Cruz Biotech, Cat #sc-23954), anti-VE-cadherin (Santa Cruz Biotech, Cat# sc-9989), anti-insulin receptor (Cell Signaling, Cat #234135), anti-insulin receptor substrate-1 (Cell Signaling, Cat #3407T), anti-insulin receptor substrate-2 (Cell Signaling, Cat #4502), mouse anti-rabbit (Santa Cruz Biotech, Cat #sc-2357), goat anti-mouse (lnvitrogen, Cat #31430), and mouse anti-goat (Santa Cruz Biotech, Cat #sc-2354).
Validation	In the project the following antibodies were validated by western blotting, by the following means: 1) knockdown: ERalpha and SNX5, 2) detection in different subcellular fractions: GAPDH, Calnexin, VE-cadherin, 3) stimulation of phosphorylation: phospho-Akt, and 3) confirmation of cells obtained using the antibody for cell isolation: CD31. For other antibodies the validation was done by query of the Research Resource Identification Portal, or RRID (https://scicrunch.org/resources).The RRID citations are as follows: Anti-Phospho-Akt (Ser473): RRID:AB_329825, anti-Akt: RRID:AB_329827, rat monoclonal anti-mouse CD31: RRID: AB_10376006, anti-mouse Mac-1: RRID:AB_2296385, anti-ERalpha: RRID:AB_627558, anti-SNX5: RRID:AB_305219, anti-GAPDH: RRID:AB_627678, anti-Calnexin: RRID:AB_626783, anti-VE-cadherin: RRID:AB_2077957, anti-insulin receptor: RRID:AB_2924796, anti-insulin receptor substrate-1: RRID:AB_2127860, anti-insulin receptor substrate-2: RRID:AB_2125774.

Eukaryotic cell lines

Policy information about cell lines and Sex and Gender in Research

Cell line source(s)	Primary human aortic endothelial cells (HAEC) were purchased from Lonza (cat#: CC-2535), and human skeletal muscle microvascular endothelial cells (HSMEC) were purchased from Cell Biologics (cat#: H-6220). The cells were from female donors. Studies were additionally performed in primary skeletal muscle endothelial cells isolated from male mice with normal versus deficient expression of estrogen receptor alpha in endothelial cells.
Authentication	The human cells were authenticated to be endothelial cells by confirming the expression of the endothelial markers CD31/105 and van Williebrand Factor VIII.
Mycoplasma contamination	The cells were tested negative for mycoplasma, bacteria, yeast, and fungi.
Commonly misidentified lines (See ICLAC register)	None used.

Animals and other research organisms

Policy information about studies involving animals; ARRIVE guidelines recommended for reporting animal research, and Sex and Gender in Research

Laboratory animals	Mice with or without ERalpha expression in endothelial cells (generated by breeding ERalpha floxed mice and VECad-Cre mice) were placed on an standard chow (males) or in a high fat diet for 12. To isolate ribosomes from skeletal muscle endothelial cells, Rosa26

	fsTRAP mice were bred with VECad-Cre mice, and studies were performed in females. In male mice studies began at 5 weeks of age, and in female mice they began at 6 weeks of age.
Wild animals	Wild animals were not used.
Reporting on sex	Experiments were performed in both male and female mice, whose sex was determined by physical examination. The study designs differed in male and female mice such that the effects of administered estrogen could be evaluated in females. The data have not been combined. The overall number of mice studied was not collected.
Field-collected samples	Field-collected samples were not used.
Ethics oversight	All animals were treated and cared for in accordance with the Guide for the Care and Use of Laboratory Animals [National Institutes of Health (NIH), Revised 2011], and the Institutional Animal Care and Use Committee of the University ofTexas Southwestern Medical Center approved all experiments.

Note that full information on the approval of the study protocol must also be provided in the manuscript.
