# Supplementary material for: Endothelial ERα promotes glucose tolerance by enhancing endothelial insulin transport to skeletal muscle
Source: Nat Commun. 2023 Aug 17;14:4989. doi: 10.1038/s41467-023-40562-w (PMC10435471; doi:10.1038/s41467-023-40562-w)
Supplement: Supplementary file 6 — Source Data [file 41467_2023_40562_MOESM6_ESM.zip › EC ER IR.NAT COMMUN.OrigMainBlots.draft1.062623.pptx]

## Slide 1
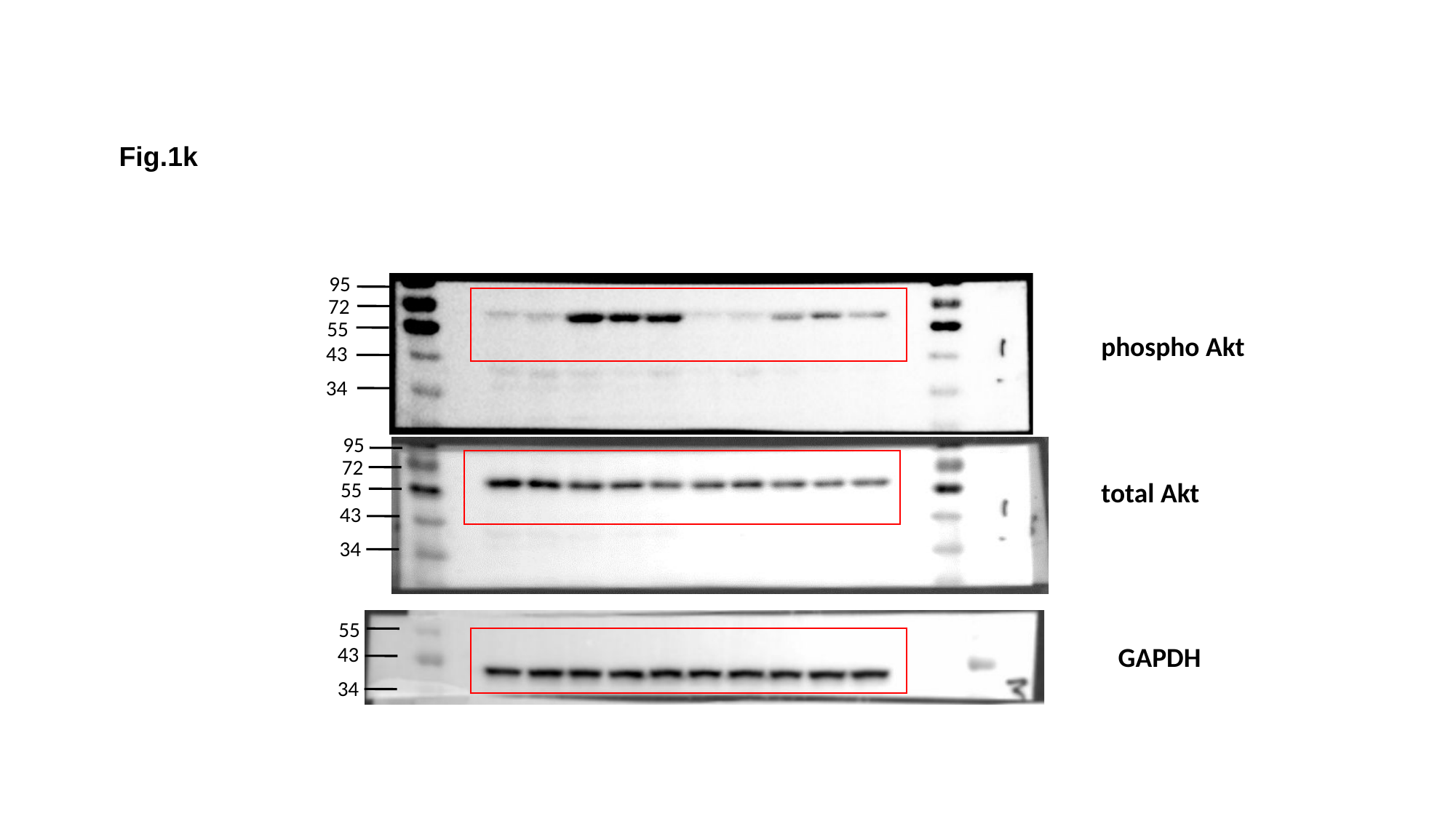

Fig.1k
95
72
55
phospho Akt
43
34
95
72
total Akt
55
43
34
55
GAPDH
43
34

## Slide 2
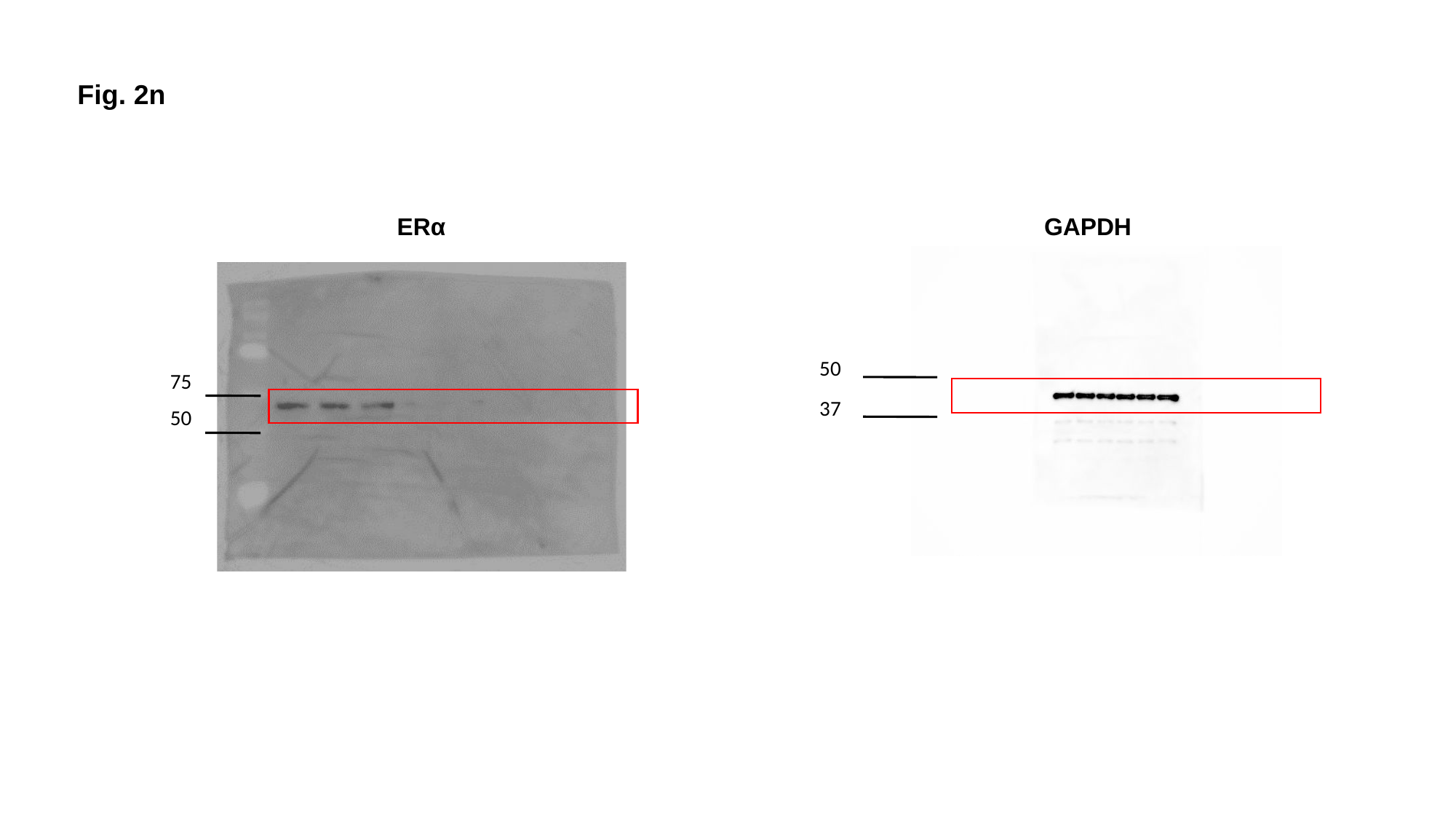

Fig. 2n
ERα
75
50
GAPDH
50
37

## Slide 3
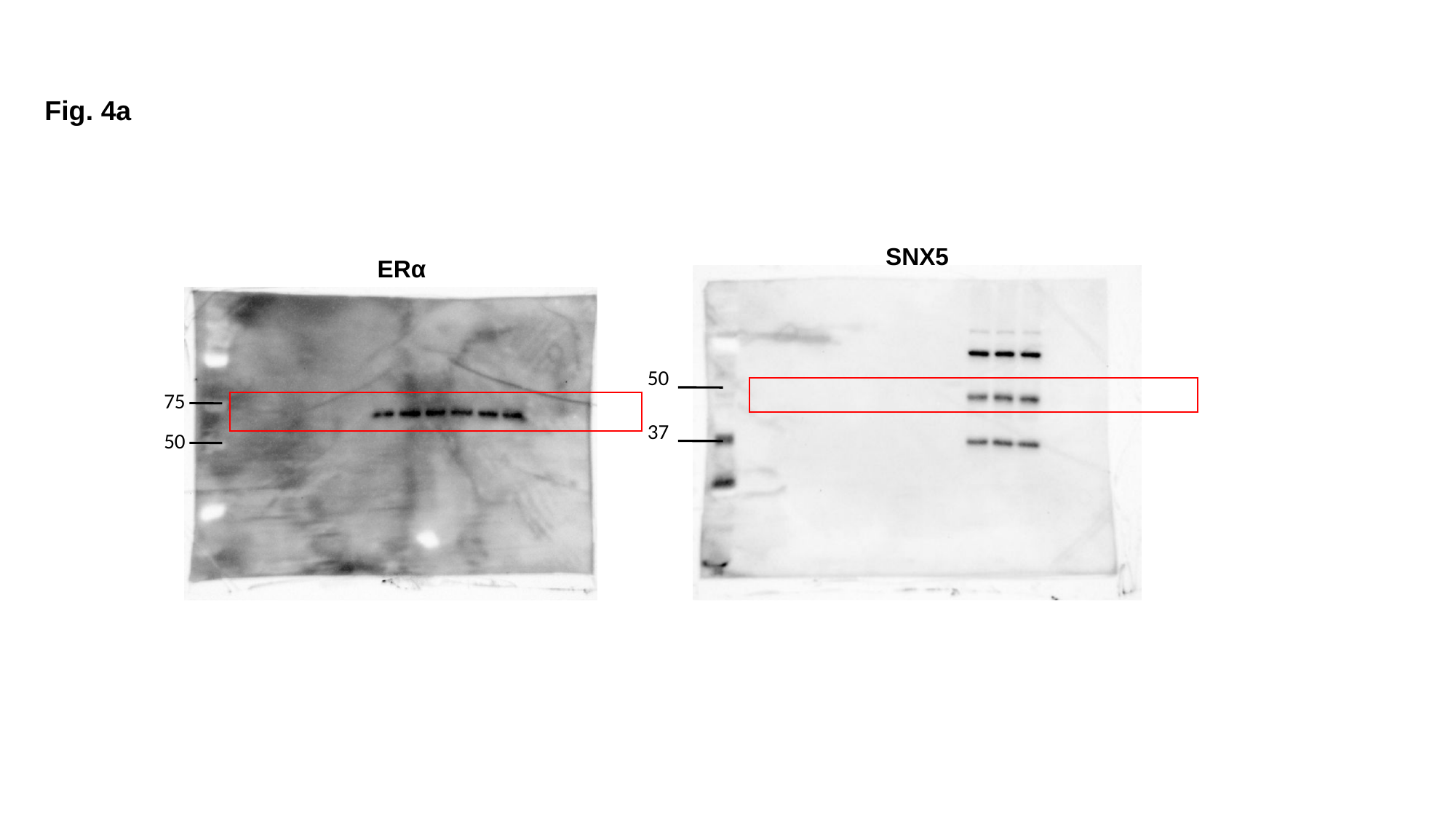

Fig. 4a
SNX5
ERα
50
37
75
50

## Slide 4
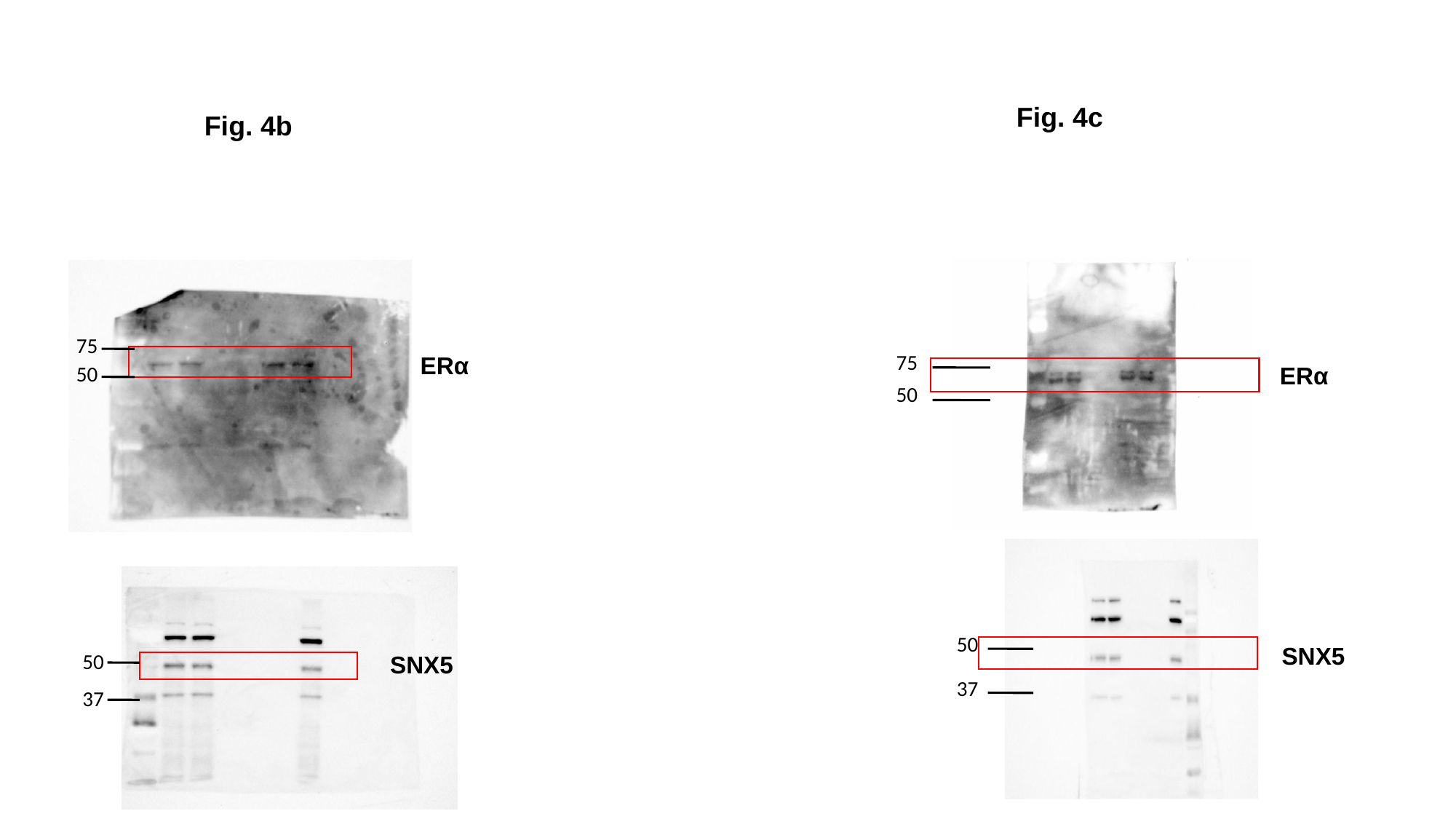

Fig. 4c
Fig. 4b
75
ERα
50
75
ERα
50
50
SNX5
37
50
SNX5
37

## Slide 5
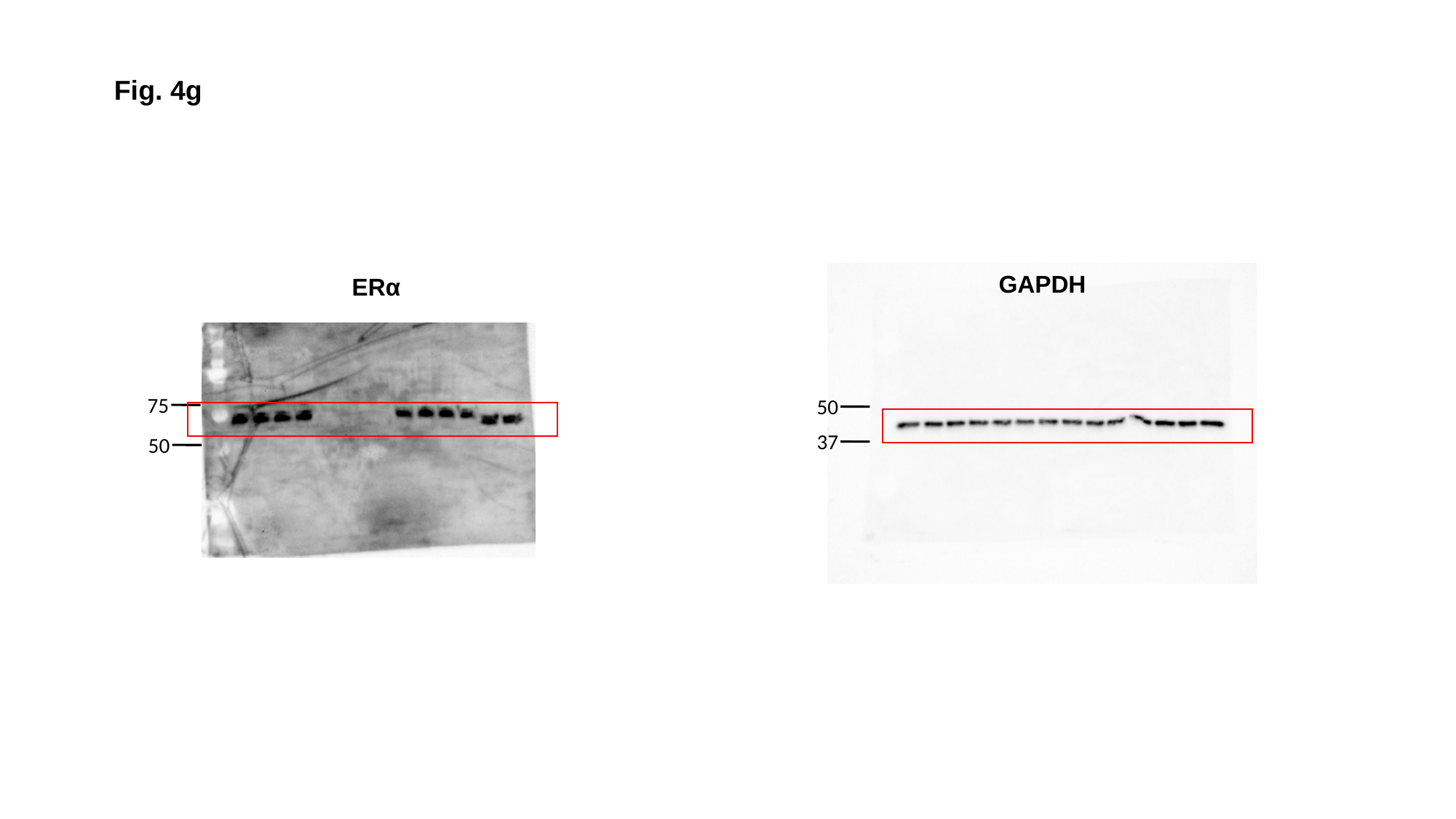

Fig. 4g
GAPDH
ERα
75
50
37
50

## Slide 6
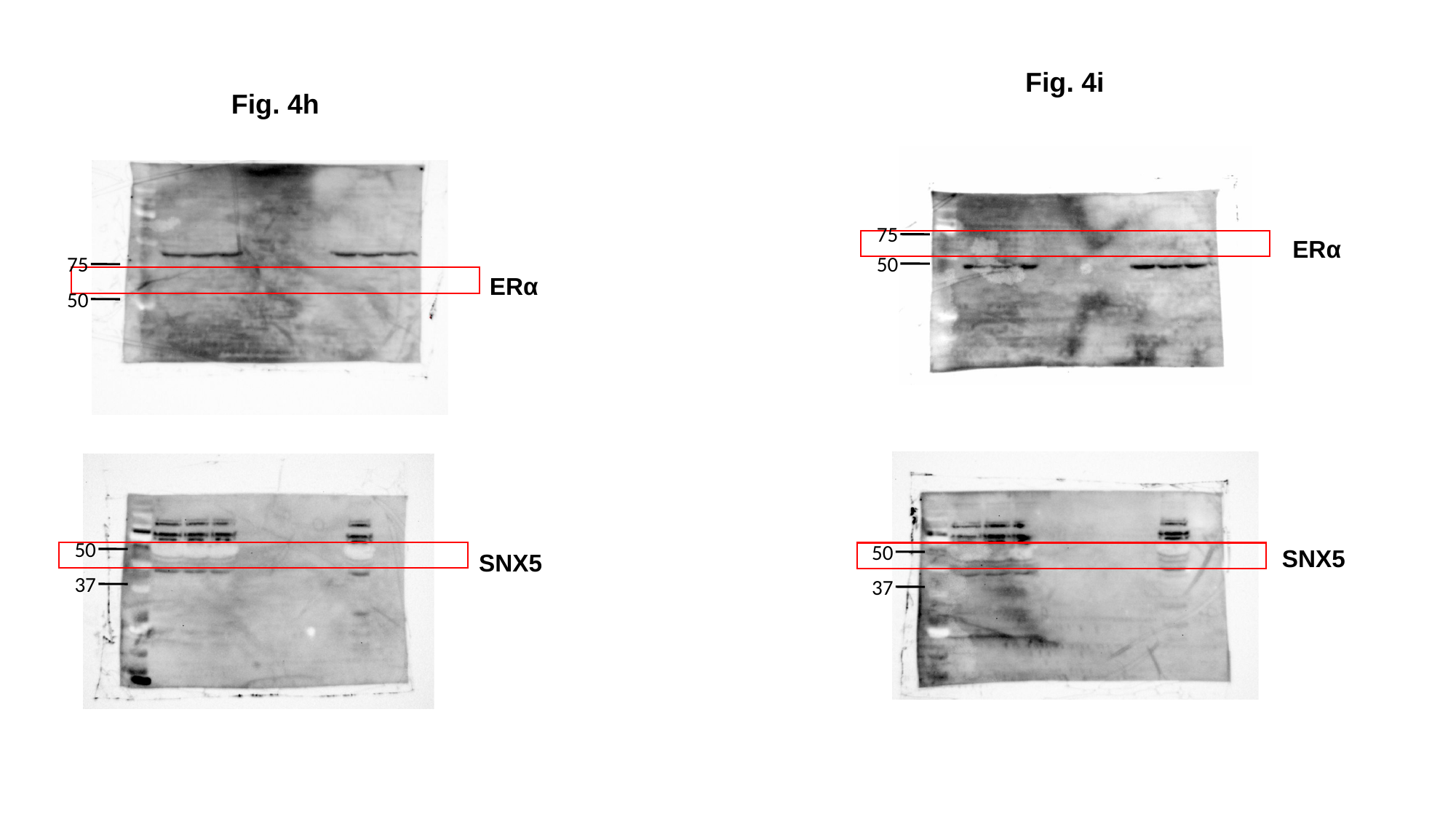

Fig. 4i
Fig. 4h
75
ERα
50
75
ERα
50
50
50
SNX5
SNX5
37
37

## Slide 7
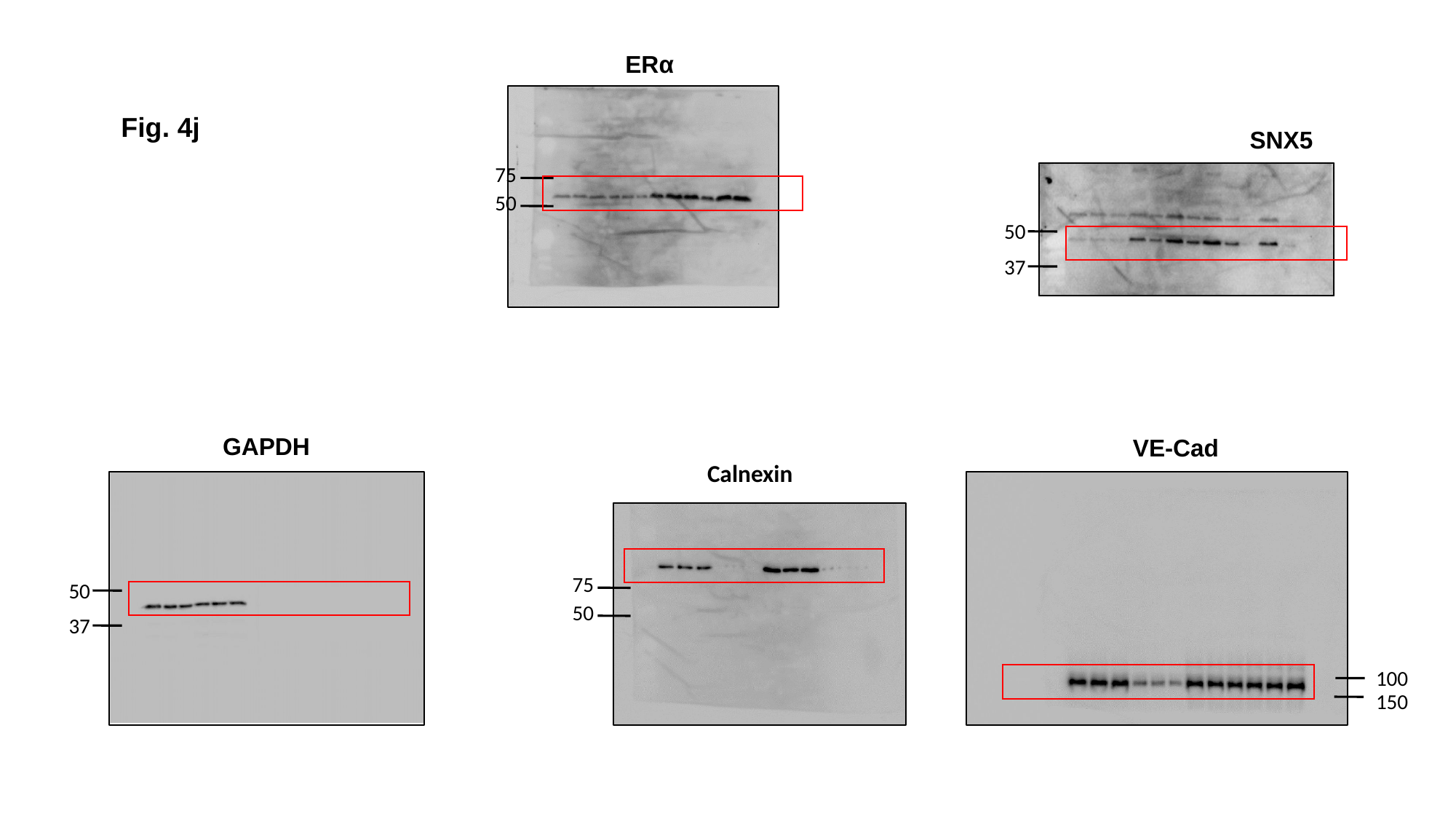

ERα
Fig. 4j
SNX5
50
37
75
50
GAPDH
50
37
VE-Cad
Calnexin
75
50
100
150

## Slide 8
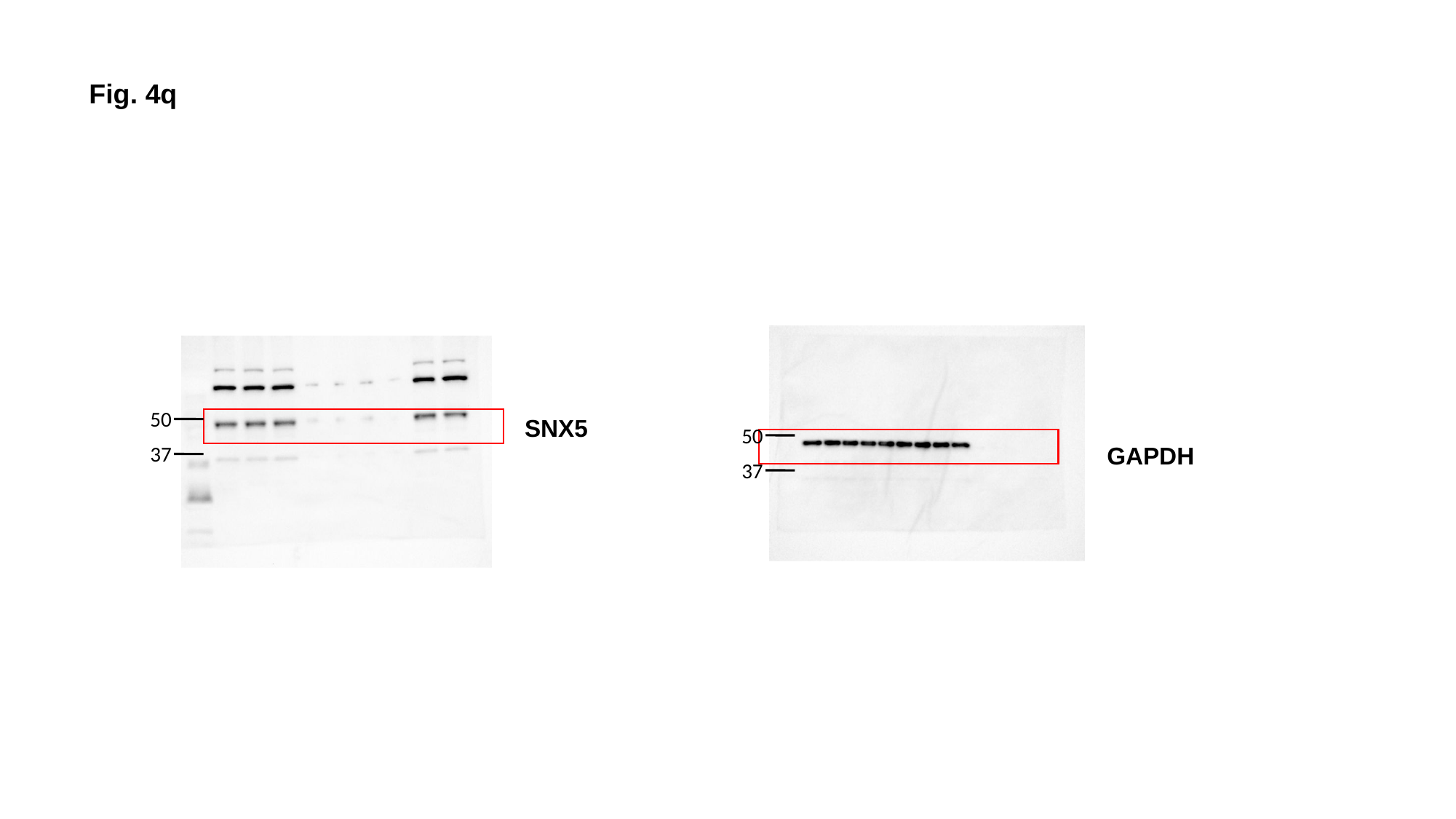

Fig. 4q
50
SNX5
50
GAPDH
37
37
